# Reduced WNT4 expression in normal skin fibroblasts leads to ‘Dupuytren-like’ changes in the transcriptome

**DOI:** 10.1016/j.heliyon.2024.e38016

**Published:** 2024-09-17

**Authors:** Aoife O'Brien, Andrew Stevenson, Lucy Barrett, Nicholas B. Lawler, Nicole Hortin, Zhenjun Deng, Amira Allahham, Fabio Quondamatteo, Nicole Smith, K. Swaminathan Iyer, Fiona M. Wood, Mark W. Fear

**Affiliations:** a Department of Anatomy and Regenerative Medicine, Royal College of Surgeons, Ireland; b Burn Injury Research Unit (BIRU), School of Biomedical Sciences, University of Western Australia, Australia; c School of Molecular Sciences, The University of Western Australia, Perth, WA, 6009, Australia; d Burns Service of Western Australia, WA Department of Health, Perth, WA, Australia; e Fiona Wood Foundation, Murdoch, WA, Australia

## Abstract

**Background:**

Dupuytren's disease (DD) is a fibro-proliferative disorder of unknown aetiology. Previous studies have implicated multiple WNT signalling genes/proteins in Dupuytren pathology, including WNT4. However, it is not yet clear whether WNT signalling dysregulation plays an important role in the initiation of the disease or progression. The aim of this study was to determine if loss of WNT4 expression triggered ‘Dupuytren-like’ changes in the transcriptome of healthy skin fibroblasts.

**Methods:**

Fibroblasts were isolated from the wrists of healthy adult males and from the wrists and disease cord tissue from males in a family positive for Dupuytren's disease. Normal skin fibroblasts from healthy controls were treated with WNT4 siRNA and scrambled controls. RNASeq was used to analyse the transcriptomes of disease and non-disease fibroblasts from patients with Dupuytren's as well as in siRNA treated and non-treated control fibroblasts.

**Results:**

Analysis of the transcriptomes from DD patient and normal skin fibroblasts showed significant differences, including in WNT4 expression. Downregulation of WNT4 in normal skin fibroblasts using siRNA led to ‘DD-like’ changes in the transcriptome.

**Conclusion:**

In people susceptible to DD WNT4 is downregulated even in non-fibrotic fibroblasts. Knockdown of WNT4 in normal fibroblasts led to changes that made cells ‘DD-like’. This study shows that WNT4 is down regulated in ‘non-disease’ cells, and that downregulating WNT4 in normal skin fibroblasts leads to widespread ‘DD like’ changes in the transcriptome, suggesting WNT4 downregulation is a key driver of DD.

## 1 Introduction

Dupuytren's disease (DD) is a localised fibrotic disease of the hand and has a significant genetic component, with automosomal dominant inheritance patterns with incomplete penetrance common in families [1]. As in all fibrotic diseases fibroblasts are the key cell type involved in the accumulation of the excess extracellular matrix, predominantly collagen I, that underpins the progression of DD [[1], [2], [3]]. However, despite a clear understanding of the pathophysiology that underpins DD disease progression, less is known about the critical triggers that initiate the disease [3,4].

The WNT signalling pathway has previously been identified as being associated with DD, through both Genome Wide Association Studies (GWAS) and a meta-analysis of these GWAS studies [[5], [6], [7]]. These studies identified WNT2, WNT4, WNT7B and SFRP4 amongst critical WNT signalling pathway genes in loci linked to Dupuytren's disease. Similarly, in a systematic review of 2373 studies, WNT signalling has been linked to both pulmonary and musculoskeletal (MSK)fibrosis, with the majority overlap between pulmonary fibrosis and DD pathologic states [8]. Other studies focused on active disease tissue have also implicated changes to key WNT signalling proteins in the progression of the disease [9,10]. However, the triggers for initiation of DD and the potential role of WNT signalling in this process remain unclear. The aim of this study was to investigate the role of WNT signalling in fibroblasts using RNASeq to examine if changes in WNT signalling may be involved in triggering the disease.

## 2 Methods

### 2.1 Ethical considerations

This study complied with the National Health and Medical Research Council statement on ethical conduct of human studies and were approved by the Notre Dame Human Research Ethics Committee (011002F**)** and by the South Metropolitan Health Service Human Ethics Committee (RGS00000099). All participants received a patient information sheet and had any questions answered prior to providing voluntary informed consent and the skin biopsy being taken using a 3 mm punch biopsy.

### 2.2 Cell isolation

3 mm tissue biopsies were taken and the explant method used to obtain fibroblast cells in culture. Briefly – 3 mm pieces of skin were placed dermis side down on the bottom of a T-25 flask, and incubated at 37 °C for 1 h. DMEM-Glutamax™ (Invitrogen Gibco) with 10 % FBS (Invitrogen Gibco), 1 % pen/strep (Invitrogen Gibco), 1 % kanamycin (Invitrogen Gibco) and 1 % amphotericin B (Invitrogen Gibco) was then added, ensuring the skin did not detach from the flasks. These were then cultured until dermal fibroblasts migrated from the explants, with media changed every 3 days. Three different locations were used for cell collection; 1. Disease cord from patients with dupuytren disease. 2. Wrist skin from the same patients with dupuytren disease (Dupuytren control) and 3. Healthy wrist skin from participants with no history or current dupuytren disease (normal skin controls).

### 2.3 WNT4 siRNA transfection

WNT4 siRNA (Cat. No. 1027416, Qiagen) was transfected into fibroblasts from normal skin wrist controls. Prior to transfection, 2.4 × 10^5^ cells were seeded in each well of a 6 well plate, in 2.2 ml of DMEM-Glutamax™+10 % FBS and 1 % penicilling/streptomycin. For 3 h prior to transfection, cells were incubated under normal growth conditions (37 °C 5 % CO_2_). 300 ng of WNT4 knockdown siRNA and control siRNA (AllStars Hs Cell Death control, Cat. No. 1027298 and Allstars Negative Control siRNA, Cat. No. 1027280, Qiagen) was diluted in 200 μL serum-free culture medium. 12 μL HiPerFect Transfection Reagent was added to the diluted siRNA and vortexed. Samples were then incubated for 5–10min at room temperature (15–25 °C) then added dropwise to the fibroblasts. The plate was gently swirled to promote uniform distribution of the transfection complexes. The cells were incubated under normal growth conditions and analysed 72 h after transfection.

### 2.4 RNA preparation and quality control (QC)

Cells were isolated using 0.05 % trypsin and washed in Phosphate Buffered Saline (PBS), before RNA extraction using the RNEasy mini kit as per manufacturer's instructions (Cat. No. 74104, Qiagen). Samples were quantified using a NanoDrop™ 1000 Spectrophotometer and RNA quality measured using an Agilent RNA 6000. Samples were sequenced at the Australian Genome Research Facility (AGRF). Illumina NovaSeq Control Software (v1.6.0) for image analysis and Real Time Analysis (v3.4.4) for real-time base calling was used, producing a 100 bp single end run.

### 2.5 RNAseq data analysis

Processing and analysis was done following our standard pipeline as previously reported [11]. Briefly, QC of raw reads was performed using FastQC (Version 0.11.3) and reads of poor quality trimmed using fastp software [12]. Raw RNA sequences were aligned to the Human GRCh38.p102 reference genome [13] using STAR read aligner (Version 2.7) [14]. BAM files representing the aligned sequences were generated using SAMtools (Version 1.9) [15].

Principal Component Analysis (PCA) and hierarchical clustering methods were performed to explore sample similarity using the rlog transformed raw read counts.

Differential gene expression analysis was performed using DESeq2 (Version 1.24.0) [16] on R (Version 3.6.1). Thresholds of adjusted p-value <0.01 and Log_2_fold-change of 1.5 were used, using the Wald test for significance with Benjamini-Hochberg correction for multiple testing.

Visualization of DEG results was conducted using the pheatmap package in R. For analysis of pathways, overrepresentation analysis on gene ontology (GO) terms and Kyoto Encyclopaedia of Genes and Genomes (KEGG) pathway analysis was run using ClusterProfiler (Version 3.12.0) [17] with a p value of <0.05 as the cut-off for significance.

## 3 Results

### 3.1 Dupuytren disease fibroblasts have a significantly different transcriptome compared to paired skin fibroblasts

**Fig. 1:**
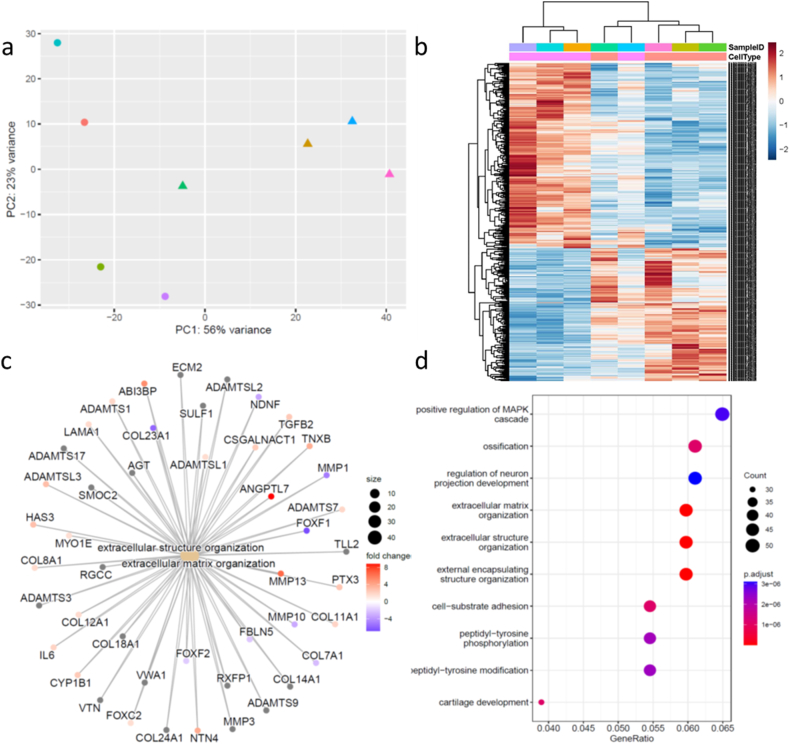
Differentially expressed genes identified in Dupuytren disease cord fibroblasts compared to paired normal skin wrist fibroblasts. (A) PCA of DD and DDC fibroblasts (triangle is DD, circle DDC). (B) Heatmap showing significant DEGs in DD (pink) vs DDC (cream) fibroblasts. (C) Concept network plot representing the expression of genes from functional categories associated with extracellular matrix. (D). The top 10 significantly enriched GO terms in the biological pathway analysis include extracellular matrix and structure organization. DD is disease cord fibroblasts, DDC are matched wrist skin fibroblasts (Dupuytren Disease Control)(For interpretation of the references to colour in this figure legend, the reader is referred to the Web version of this article.)

Initially RNASeq was used to compare the transcriptome of the DD cord fibroblast samples to their matched normal skin (wrist) isolated fibroblasts (Fig, 1, Supp Table S1). PCA analysis demonstrated separation of the two groups (Fig. 1a and b). A total of 1025 significantly differentially expressed genes were identified using a paired comparison. These genes were important in pathways including extracellular matrix organization and structure identified using GO analysis (Fig. 1c and d), with dysregulation of a number of matrix metalloproteinases, TGFβ and collagen expression. These results are aligned with the expected changes in fibroblasts isolated from fibrotic tissue compared to normal skin fibroblasts from the same individuals.

### 3.2 Fibroblasts from normal skin of DD positive participants have a significantly different transcriptome compared to fibroblasts from normal skin tissue from DD negative tissue

**Fig. 2:**
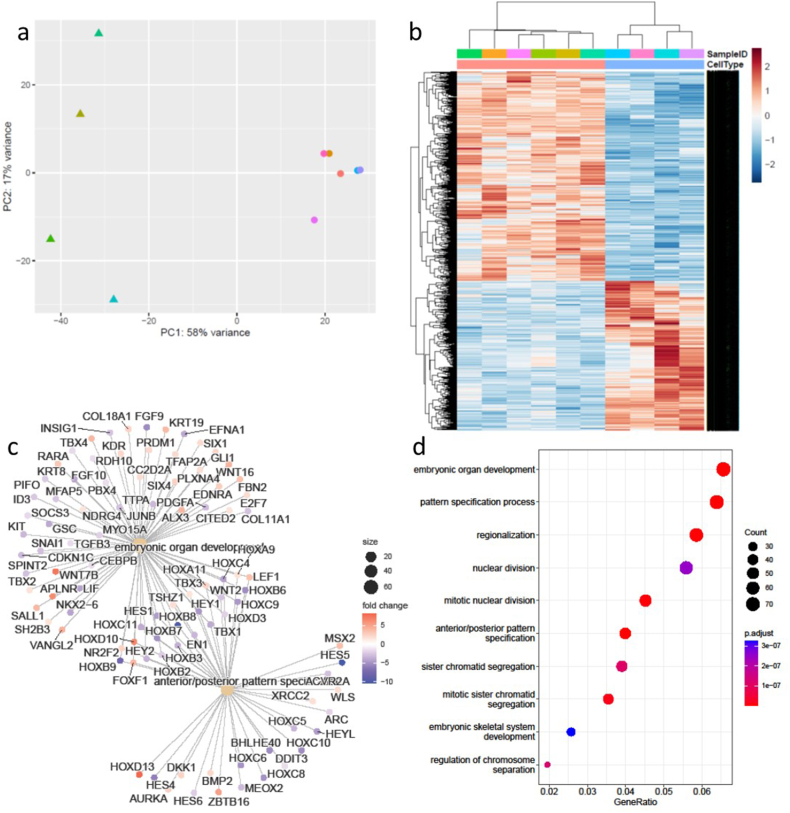
Differentially expressed genes identified in Dupuytren patient wrist fibroblasts (DDC) compared to normal skin wrist fibroblasts (NS). (A) PCA of DDC compared to NS fibroblasts (triangle is DDC, circle NS). (B) Heatmap of significantly differentially expressed genes in DDC (blue) vs NS fibroblasts (cream). (C) Concept network plot representing the expression of genes from top two functional categories (D) The top 10 significantly enriched GO terms in the biological pathway category include embryonic organ development and pattern specification. (For interpretation of the references to colour in this figure legend, the reader is referred to the Web version of this article.)

In light of the genetic predisposition to DD in the family being studied, we hypothesised that there may be significant changes even in the phenotypically normal fibroblasts isolated from the wrists of these participants that is indicative of a predisposition to develop DD compared to those from healthy non-DD participants. We therefore next compared the transcriptomes of these DD controls (DDC) samples to samples isolated from the wrists of normal skin from healthy males (NS (Fig. 2)). The DDC and NS fibroblast samples segregated in the PCA analysis (Fig. 2a). We identified 1997 differentially expressed genes between the DDC and NS fibroblast samples (Fig. 2b–Supp Table 2). Included in these differentially expressed genes were WNT2, WNT7B and WNT4, all of which have been previously implicated in Dupuytren's disease through GWAS studies [[5], [6], [7],^10^]. Other members of the WNT signalling pathway were also differentially expressed between the DDC and NS samples including WNT16, DKK and SFRP4. SFRP4 has also previously been associated with Dupuytren's disease via GWAS analysis [[5], [6], [7]]. Both WNT4 and SFRP4 were downregulated in the DC samples compared to NS fibroblasts, whilst WNT2 and WNT7b were both upregulated (Supp Table 2). The significant dysregulation in the fibroblasts isolated from the wrist of participants with Dupuytren's disease suggest that these changes may be important in the susceptibility to subsequent development of Dupuytren's disease.

### 3.3 WNT4 knockdown in NS fibroblasts leads to a ‘DD-like’ change in the transcriptome

**Fig. 3:**
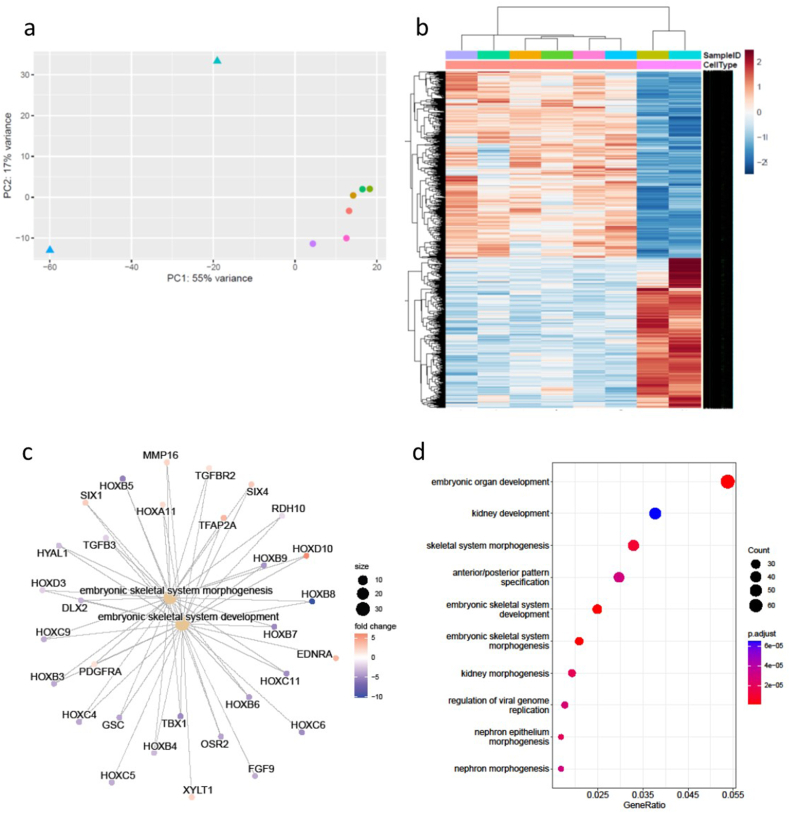
Differentially expressed genes identified in WNT4siRNA fibroblasts compared to normal skin wrist fibroblasts. (A) PCA of WNT4siRNA and NS fibroblasts (triangle is WNT4siRNA, circle NS). (B) Heatmap of significantly differentially expressed genes in WNT4siRNA (pink) vs NS fibroblasts (cream). (C) Concept network plot representing the expression of genes from functional categories associated with extracellular matrix. (D) The top 10 significantly enriched GO terms in the biological pathway category include embryonic skeletal system development. (For interpretation of the references to colour in this figure legend, the reader is referred to the Web version of this article.)

To further investigate the impact of WNT dysregulation on the fibroblast transcriptome, we decided to investigate whether the reduction in WNT4 expression observed in DDC fibroblasts was linked to the changes in the fibroblast transcriptome. We used an siRNA approach to reduce WNT4 expression in two normal skin fibroblast samples from the original NS cohort. We then performed RNASeq analysis to compare the profile of the WNT4siRNA samples to NS fibroblast samples only. The WNT4siRNA samples had 2209 significantly differentially expressed genes when compared to the NS samples (Fig. 3, Supp Table 3), similar to the number of genes significantly differentially expressed in the DDC samples when compared to NS samples (Supp Table 2). Within these differentially expressed genes WNT16, WNT7B and DKK1 were all significantly upregulated in WNT4siRNA fibroblasts compared to NS fibroblasts, as was observed in the DDC fibroblasts. SFRP4 was also significantly decreased in expression in the WNT4siRNA samples, again as observed in the DDC samples, whilst WNT4 was decreased (log_2_fold change 3.35). Other genes previously linked to DD were also significantly altered in expression, including AURKC (Supp Table 3), whilst genes previously linked to fibrosis, including TGFBR2, TGFβ3 and SNAI1 were also dysregulated after WNT4 siRNA treatment.

**Fig. 4:**
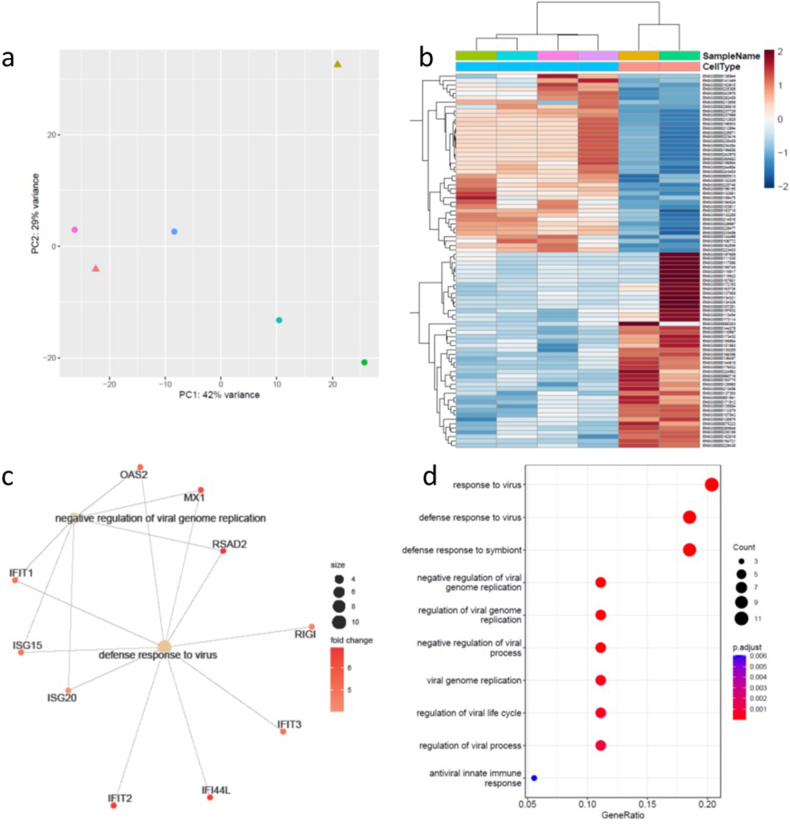
Differentially expressed genes identified in WNT4siRNA fibroblasts compared to DDC fibroblasts. (A) PCA of DDC and WNT4siRNA fibroblasts (triangle is WNT4siRNA, circle DDC). (B) Heatmap of significantly differentially expressed genes in DD (blue) vs WNT4siRNA (cream) fibroblasts. (C) Concept network plot representing the expression of genes from functional categories associated with extracellular matrix. The selected genes were identified based on GO analysis. (D) The top 10 significantly enriched GO terms in the biological pathway category include defense response to virus and negative regulation of viral gene replication. (For interpretation of the references to colour in this figure legend, the reader is referred to the Web version of this article.)

Given the apparent similarity of a range of changes observed in the WNT4siRNA samples to those observed in DDC fibroblasts, we next compared the WNT4siRNA to the DDC fibroblast samples (Fig. 4, Supp Table 4). Only 89 genes were significantly differentially expressed between these two groups, with the PCA analysis showing poor segregation of WNT4siRNA and DDC samples (Fig. 4a). Pathway analysis indicated these differentially expressed genes were associated with the viral response. These genes are commonly upregulated in response to siRNA approaches and therefore this indicates the differences observed between these two groups are likely an artefact of the use of siRNA to diminish WNT4 expression in the NS fibroblasts.

To further assess whether the WNT4siRNA was exerting specific effects, the same analysis was conducted using scrambled control siRNA transfected normal skin fibroblasts compared to DDC fibroblasts (Supp. Fig. 1, Supp. Table 5). 719 genes were significantly differentially expressed, with PCA analysis showing clear segregation of samples, and pathway analysis showing extracellular matrix and collagen related pathways being significantly different between the two groups, in addition to the viral response pathway, demonstrating that the scrambled siRNA had no similar effects on genes relevant to fibrosis.

**Fig. 5:**
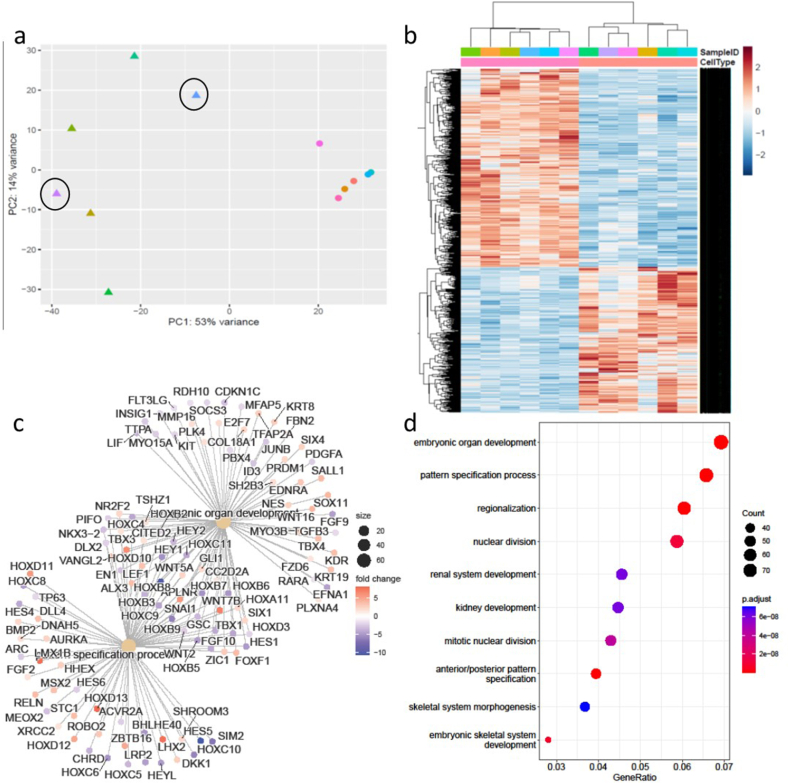
Differentially expressed genes identified in Dupuytren disease cord fibroblasts and WNT4 siRNA treated normal skin fibroblasts compared to paired normal skin wrist fibroblasts. (A) PCA of the DD and DDC fibroblasts (triangle is DD or WNT4siRNA treated NS fibs, circle normal skin fibroblasts). (B) Heatmap of significantly differentially expressed genes in DD/WNT4 siRNA vs NS fibroblasts. (C) Concept network plot representing the expression of genes from functional categories associated with organ development. (D) GO analysis identified over-representative GO terms for the DEGs in treated fibroblasts. The top 10 significantly enriched GO terms in the biological pathway category include embryonic organ development.

Finally, we compared the DDC and WNT4siRNA samples to NS fibroblasts in a single analysis to observe similarity of the two groups when compared to NS fibroblasts (Fig. 5, Supp. Table 6). The WNT4siRNA samples clustered with the DDC samples (Fig. 5a) and were significantly different to the normal skin fibroblast controls (Fig. 5b–d). 2250 genes were significantly differentially expressed between the combined DDC/WNT4siRNA group and the NS controls (Supp. Table 6). Over 80 % of the significantly differentially expressed annotated genes in this analysis were identical to those identified in the DDC vs NS only analysis, suggesting the changes after WNT4 siRNA treatment were very similar to those observed in DDC fibroblasts.

## 4 Discussion

In this study, we have compared NS, DDC and DD fibroblast samples using RNASeq. This approach demonstrated significant differences between DD fibroblasts and their matched controls. As expected this highlighted matrix production changes by the fibroblasts as the disease develops. More surprisingly, the apparently normal skin fibroblasts from patients with Dupuytren's disease compared to health control skin fibroblasts demonstrated significant changes, in particular with WNT signalling pathway gene expression. The use of siRNA to downregulate WNT4 in normal skin fibroblasts further demonstrated that WNT signalling dysregulation appeared to be critical to the transition from a ‘normal, healthy’ fibroblast transcriptome to a ‘pre-Dupuytren’ transcriptome.

The WNT signalling pathway has previously been implicated in DD, most definitively through GWAS studies linking a high number of WNT signalling gene loci to the occurrence of the disease [[5], [6], [7]]. However, our understanding of the triggers of the disease remain limited. The work presented here suggests that the genetic predisposition to Dupuytren disease is detectable in significant and wide-ranging changes in the transcriptome of fibroblasts isolated even from phenotypically normal skin. This suggests that at least one of the key factors in the initiation of the disease resides directly in changes in the fibroblasts of affected patients.

The changes observed after treatment of normal skin fibroblasts with WNT4 siRNA also suggests that this change not only affects the transcriptome but directly affects a range of genes previously linked to Dupuytren's disease and/or fibrosis, including AURKC, SFRP4, both linked in previous GWAS studies [18], as well as members of the TGFβ signaling pathway.

The further evidence provided here of WNT pathway dysregulation playing a significant role in the pathophysiology of Dupuytren's disease adds to the increasing weight of evidence from genetic and other study approaches that WNT signalling is critical. Nevertheless there remains the significant question of what the potential ‘second-trigger’ is that initiates the fibrotic progression in the palmar fascia. Whilst this study shows dysregulation of WNT signalling is evident in the fibroblasts, implicating this cell type in the initiation of the disease, given the normal skin phenotype observed in patients with Dupuytren's disease it is clear that this dysregulation alone does not trigger fibrosis. A number of alternatives exist for the development of DD. Under mechanical stress, fibroblasts can undergo a two-stage differentiation process, first into proto-myofibroblasts, which, together with the expression of pro-fibrotic factors and the increase in mechanical tension subsequently differentiate into myofibroblasts [19]. Alternatively, given that DD is strongly associated with diabetes it maybe that the lack of vascular supply and subsequent hypoxia are important in altering the fibroblast phenotype and initiating fibrosis. Studies have clearly shown that vascular changes can influence fibroblast phenotype changes, including in Dupuytren's disease [20,21], with hypoxia leading to a more myofibroblast phenotype. The immune system may also play a role as has been suggested in previous studies, either directly through stimulation of fibroblast activity or it may be involved indirectly through damage to the vascular supply that may in turn trigger fibroblast changes [22,23]. Given the findings presented here, the use of DDC and normal skin fibroblasts in models to mimic hypoxia and/or mechanical stress or other triggers, together with the use of siRNA and RNASeq analysis, may provide insight into how the cells transition from the DDC ‘Pre-Dupuytren’ transcriptome to a DD ‘Dupuytren disease’ transcriptome.

Limitations of this study include the use of a single family to provide the Dupuytren disease tissue and control samples. However, the samples treated with WNT4siRNA were not from this family and therefore the similarity in changes observed suggests it is related to the Dupuytren phenotype rather than familial connection. This study is also limited with small sample numbers overall, although the use of paired samples from control and disease tissue in the family samples provides a close matched control sample for those analyses. Finally, the study only shows differences in the transcriptome and there is not functional data that validates that the changes are linked to fibrosis or Dupuytren's disease. Nevertheless, the changes in the transcriptome may lead to phenotypic changes in the fibroblasts that increase susceptibility to the development of Dupuytren's disease, and the presence of significant changes in expression of a number of genes previously implicated in fibrosis, suggests WNT4 expression is important in DD pathophysiology.

This study demonstrates significant changes in the fibroblast transcriptome exist between apparently phenotypically normal fibroblasts from the wrist of patients with Dupuytren's disease and the wrists of healthy controls. These changes include significant dysregulation of a range of WNT signalling genes previously implicated in Dupuytren's disease through GWAS studies. We also demonstrate that a reduction in WNT4 expression can trigger very similar transcriptome changes in normal skin fibroblasts, suggesting WNT dysregulation can generate a ‘DD-like’ transcriptome in normal fibroblasts. This provides important new evidence for a role for WNT signalling in the initiation of Dupuytren's disease. Further studies to characterise these changes functionally may lead to more insight into the potential therapeutic options for ameliorating this disease.

## Declarations

This study was conducted in compliance with the National Health and Medical Research Council statement on ethical conduct of human studies. The study was approved by the Notre Dame Human Research Ethics Committee (011002F**)** and the South Metropolitan health Service Human Ethics committee (RGS00000099). All participants provided voluntary informed consent.

## Funding

This work was supported by the 10.13039/501100022218Fiona Wood Foundation. MWF is supported by the 10.13039/501100020265Stan Perron Charitable Foundation and the Perth Children's Hospital Foundation.

## Data availability

Bulk RNA-Seq data is deposited at GEO and is accessible at GSE269317.

## CRediT authorship contribution statement

**Aoife O'Brien:** Writing – original draft, Investigation. **Andrew Stevenson:** Methodology, Conceptualization. **Lucy Barrett:** Methodology, Investigation. **Nicholas B. Lawler:** Methodology, Investigation. **Nicole Hortin:** Project administration, Investigation. **Zhenjun Deng:** Visualization, Formal analysis. **Amira Allahham:** Writing – review & editing, Formal analysis. **Fabio Quondamatteo:** Writing – review & editing, Supervision. **Nicole Smith:** Writing – review & editing, Funding acquisition. **K. Swaminathan Iyer:** Writing – review & editing, Funding acquisition. **Fiona M. Wood:** Writing – review & editing, Funding acquisition, Conceptualization. **Mark W. Fear:** Writing – original draft, Funding acquisition, Conceptualization.

## Declaration of competing interest

The authors declare that they have no known competing financial interests or personal relationships that could have appeared to influence the work reported in this paper.

## References

[1] Michou L., Lermusiaux J.L., Teyssedou J.P., Bardin T., Beaudreuil J., Petit-Teixeira E. (2012). Genetics of Dupuytren's disease. Joint Bone Spine.

[2] Sayadi L.R., Alhunayan D., Sarantopoulos N. (2019 Nov). The molecular pathogenesis of dupuytren disease: review of the literature and suggested new approaches to treatment. Ann. Plast. Surg..

[3] Layton T.B., Williams L., Jagdeep N. (2023). Dupuytren's disease: a localised and accessible human fibrotic disorder. Trends Mol. Med..

[4] Rehman S., Goodacre R., Day P.J. (2011). Dupuytren's: a systems biology disease. Arthritis Res. Ther..

[5] Dolmans G.H., Werker P.M., Hennies (2011). Wnt signaling and Dupuytren's disease. N. Engl. J. Med..

[6] Ng M., Thakkar D., Southam L., Werker P. (2017 Sep 7). A genome-wide association study of dupuytren disease reveals 17 additional variants implicated in fibrosis. Am. J. Hum. Genet..

[7] Becker K., Siegert S., Toliat M.R. (2016). Meta-analysis of genome-wide association studies and network analysis-based integration with gene expression data identify new suggestive loci and unravel a Wnt-centric network associated with Dupuytren's disease. PLoS One.

[8] Dagneaux L., Owen A.R. (2020). Human fibrosis: is there evidence for a genetic predisposition in musculoskeletal tissues?. J. Arthroplasty.

[9] Forrester H.B., Temple-Smith P., Ham S., de Kretser D., Southwick G., Sprung C.N. (2013). Genome-wide analysis using exon arrays demonstrates an important role for expression of extra-cellular matrix, fibrotic control and tissue remodelling genes in Dupuytren's disease. PLoS One.

[10] Ten Dam E.J.P.M., van Beuge M.M., Bank R.A., Werker P.M.N. (2016). Further evidence of the involvement of the Wnt signaling pathway in Dupuytren's disease. Journal of Cell Communication and Signaling.

[11] Lewis C.J., Douglas H., Martin L., Deng Z., Melton P., Fear M.W., Wood F.M., Rea S. (2023 Sep). Carbon dioxide laser treatment of burn-related scarring: results of the ELIPSE (Early Laser Intervention Promotes Scar Evolution) prospective randomized controlled trial. J. Plast. Reconstr. Aesthetic Surg..

[12] Chen, S., Zhou, Y., Chen, Y., & Gu, J. Fastp: an Ultra-fast All-In-One FASTQ Preprocessor. (1367-4811 (Electronic)). 10.1093/bioinformatics/bty560 PMC6129281 30423086

[13] Cunningham, F., Achuthan, P., Akanni, W et al. Ensembl 2019. (1362-4962 (Electronic)).

[14] Dobin, A., Davis Ca Fau - Schlesinger, F., Schlesinger F Fau - Drenkow, J., Drenkow J Fau - Zaleski, C., Zaleski C Fau - Jha, S., Jha S Fau - Batut, P.,Gingeras, T. R. Star: Ultrafast Universal RNA-Seq Aligner. (1367-4811 (Electronic)). 10.1093/bioinformatics/bts635 PMC3530905 23104886

[15] Li, H., Handsaker B Fau - Wysoker, A., Wysoker A Fau – Fennell et al. The Sequence Alignment/Map Format and SAMtools. (1367-4811 (Electronic)). 10.1093/bioinformatics/btp352 PMC2723002 19505943

[16] Love M.I., Huber W., Anders S. (2014). Moderated estimation of fold change and dispersion for RNA-seq data with DESeq2. Genome Biol..

[17] Yu, G., Wang Lg Fau - Han, Y., Han Y Fau - He, Q.-Y., & He, Q. Y. clusterProfiler: an R Package for Comparing Biological Themes Among Gene Clusters. (1557-8100 (Electronic)). 10.1089/omi.2011.0118 PMC3339379 22455463

[18] Riesmeijer S.A., Kamali Z., Ng M. (2024). A genome-wide association meta-analysis implicates Hedgehog and Notch signaling in Dupuytren's disease. Nat. Commun..

[19] Tomasek J.J., Gabbiani G., Hinz B., Chaponnier C., Brown R.A. (2002). Myofibroblasts and mechano-regulation of connective tissue remodelling. Nat. Rev. Mol. Cell Biol..

[20] Watson S., Burnside T., Carver W. (1998). Angiotensin II-stimulated collagen gel contraction by heart ﬁbroblasts: role of the AT1 receptor and tyrosine kinase activity. J. Cell. Physiol..

[21] Layton T.B., Williams L., Yang N., Nanchahal J. (2022). A vasculature niche orchestrates stromal cell phenotype through PDGF signaling: importance in human fibrotic disease. Proc. Natl. Acad. Sci. USA.

[22] McCarty S., Syed F., Bayat A. (2010). Role of the HLA system in the pathogenesis of Dupuytren's disease. Hand.

[23] Mayerl C., Del Frari B., Parson W., Boeck G., Piza-Katzer H., Wick G., Wolfram D. (2016). Characterisation of the inflammatory response in Dupuytren's disease. J Plast Surg Hand Surg..

