# Supplementary material for: Reduced WNT4 expression in normal skin fibroblasts leads to ‘Dupuytren-like’ changes in the transcriptome
Source: Heliyon. 2024 Sep 17;10(18):e38016. doi: 10.1016/j.heliyon.2024.e38016 (PMC11458971; doi:10.1016/j.heliyon.2024.e38016)
Supplement: Multimedia component 1 [file mmc1.pdf]

Supp Table 1 DD vs DDC comparison

ENSG	baseMean	log2FoldC	lfcSE	stat	pvalue	padj	symbol	entrez	genename
ENSG0000	1445.535	7.527035	0.527443	14.2708	3.33E-46	6.80E-42	SCRG1	11341	stimulator of chondrogenesis 1
ENSG0000	30165.09	7.060163	0.529774	13.32674	1.62E-40	1.65E-36	CHI3L1	1116	chitinase 3 like 1
ENSG0000	329.011	4.880292	0.380099	12.83953	9.85E-38	6.71E-34	ITGB2	3689	integrin subunit beta 2
ENSG0000	146899.2	1.678717	0.138994	12.07762	1.39E-33	7.08E-30	COL12A1	1303	collagen type XII alpha 1 chain
ENSG0000	237.9376	2.653065	0.221998	11.95086	6.43E-33	2.63E-29	NA	NA	NA
ENSG0000	315.4139	-8.80776	0.776658	-11.3406	8.26E-30	2.81E-26	CHRM2	1129	cholinergic receptor muscarinic 2
ENSG0000	940.4362	4.495589	0.40742	11.03429	2.61E-28	7.62E-25	DAPK1	1612	death associated protein kinase 1
ENSG0000	45420.22	2.560806	0.235585	10.86999	1.60E-27	4.09E-24	ITGA11	22801	integrin subunit alpha 11
ENSG0000	1111.258	3.482956	0.32315	10.77814	4.37E-27	9.91E-24	MEDAG	84935	mesenteric estrogen dependent adipogenesis
ENSG0000	409.9789	8.270147	0.776764	10.64693	1.80E-26	3.68E-23	KRT16	3868	keratin 16
ENSG0000	2295.721	-2.91763	0.274444	-10.6311	2.14E-26	3.94E-23	NES	10763	nestin
ENSG0000	202.962	-7.71313	0.726035	-10.6236	2.31E-26	3.94E-23	IRX2	153572	iroquois homeobox 2
ENSG0000	107937.4	3.397741	0.320114	10.61416	2.56E-26	4.02E-23	CEMIP	57214	cell migration inducing hyaluronidase 1
ENSG0000	112.4467	-7.06601	0.670978	-10.5309	6.22E-26	9.08E-23	NA	NA	NA
ENSG0000	1125.164	7.932971	0.75491	10.50849	7.89E-26	1.08E-22	CSF2RB	1439	colony stimulating factor 2 receptor subunit beta
ENSG0000	172.8414	5.137919	0.493279	10.41586	2.10E-25	2.68E-22	CHRDL1	91851	chordin like 1
ENSG0000	13183.96	2.505823	0.241496	10.37626	3.18E-25	3.82E-22	MFAP5	8076	microfibril associated protein 5
ENSG0000	328.1161	-9.04362	0.872142	-10.3694	3.42E-25	3.88E-22	LHX8	431707	LIM homeobox 8
ENSG0000	13704.49	5.124799	0.496341	10.32515	5.42E-25	5.83E-22	ABI3BP	25890	ABI family member 3 binding protein
ENSG0000	2505.719	4.274287	0.430347	9.93219	3.02E-23	3.08E-20	CILP2	148113	cartilage intermediate layer protein 2
ENSG0000	524.8223	7.181062	0.736171	9.754611	1.76E-22	1.64E-19	PRG4	10216	proteoglycan 4
ENSG0000	543.3207	5.631632	0.584584	9.633569	5.77E-22	5.12E-19	PNMA2	10687	PNMA family member 2
ENSG0000	241.7267	2.972251	0.316612	9.387674	6.13E-21	5.22E-18	CGREF1	10669	cell growth regulator with EF-hand domain 1
ENSG0000	475.4362	2.348225	0.252826	9.287925	1.57E-20	1.29E-17	CSGALNAC	55790	chondroitin sulfate N-acetylgalactosaminyltransferase 1
ENSG0000	448.6361	6.431914	0.698437	9.209005	3.29E-20	2.59E-17	MEOX2	4223	mesenchyme homeobox 2
ENSG0000	4428.194	-4.96976	0.541405	-9.17937	4.34E-20	3.28E-17	MMP1	4312	matrix metallopeptidase 1
ENSG0000	911.8341	2.231888	0.243611	9.161669	5.11E-20	3.73E-17	JAG1	182	jagged canonical Notch ligand 1
ENSG0000	218.2144	-7.94676	0.867985	-9.15541	5.42E-20	3.81E-17	SPHKAP	80309	SPHK1 interactor, AKAP domain containing
ENSG0000	1099.783	5.057988	0.561707	9.004675	2.16E-19	1.47E-16	PAPPA2	60676	pappalysin 2
ENSG0000	5360.999	4.775755	0.530903	8.995539	2.35E-19	1.55E-16	VCAM1	7412	vascular cell adhesion molecule 1
ENSG0000	529.9509	2.15476	0.241353	8.927823	4.34E-19	2.77E-16	NA	NA	NA
ENSG0000	120.0513	-10.0693	1.131351	-8.90028	5.57E-19	3.45E-16	SHOX	6473	short stature homeobox
ENSG0000	2243.905	-6.39659	0.726202	-8.80828	1.27E-18	7.64E-16	SFRP1	6422	secreted frizzled related protein 1
ENSG0000	128.7655	-4.17071	0.486586	-8.57138	1.02E-17	5.97E-15	CES1	1066	carboxylesterase 1
ENSG0000	2228.168	-1.69864	0.198357	-8.56357	1.09E-17	6.21E-15	SCD	6319	stearoyl-CoA desaturase
ENSG0000	329.7563	5.667848	0.666194	8.507798	1.77E-17	9.53E-15	SCN3A	6328	sodium voltage-gated channel alpha subunit 3
ENSG0000	251.7749	4.292915	0.510053	8.416607	3.88E-17	1.98E-14	SIX2	10736	SIX homeobox 2
ENSG0000	292.9428	-1.72861	0.205737	-8.40201	4.39E-17	2.19E-14	CNIH3	149111	cornichon family AMPA receptor auxiliary protein 3
ENSG0000	391.6661	4.461005	0.533867	8.356022	6.49E-17	3.16E-14	PLXDC2	84898	plexin domain containing 2
ENSG0000	1734.51	4.227235	0.50697	8.338228	7.54E-17	3.58E-14	PDE1C	5137	phosphodiesterase 1C
ENSG0000	107.7029	4.014682	0.488419	8.219742	2.04E-16	9.06E-14	PPM1H	57460	protein phosphatase, Mg2+/Mn2+ dependent 1H
ENSG0000	970.2533	6.248413	0.763943	8.179163	2.86E-16	1.24E-13	SORBS2	8470	sorbin and SH3 domain containing 2
ENSG0000	332.147	8.507426	1.042724	8.158849	3.38E-16	1.44E-13	EPGN	255324	epithelial mitogen
ENSG0000	254.5113	-3.68856	0.45621	-8.08522	6.21E-16	2.59E-13	RNF112	7732	ring finger protein 112
ENSG0000	40816.3	2.596123	0.322439	8.051504	8.18E-16	3.34E-13	PTX3	5806	pentraxin 3
ENSG0000	410.6385	1.689942	0.211433	7.992804	1.32E-15	5.28E-13	PTK2B	2185	protein tyrosine kinase 2 beta
ENSG0000	202.7744	3.981305	0.498796	7.981826	1.44E-15	5.66E-13	NA	NA	NA
ENSG0000	35323.77	4.245916	0.532984	7.966316	1.63E-15	6.30E-13	EFEMP1	2202	EGF containing fibulin extracellular matrix protein 1
ENSG0000	30558.26	1.613225	0.203607	7.923228	2.31E-15	8.76E-13	MFGE8	4240	milk fat globule EGF and factor V/VIII domain containing
ENSG0000	251.0476	3.000053	0.379657	7.902018	2.74E-15	1.02E-12	ANKRD6	22881	ankyrin repeat domain 6
ENSG0000	5880.101	2.058177	0.262184	7.85011	4.16E-15	1.52E-12	MEGF6	1953	multiple EGF like domains 6
ENSG0000	147.398	2.932299	0.376369	7.791022	6.65E-15	2.38E-12	HAS3	3038	hyaluronan synthase 3
ENSG0000	3576.68	-5.95101	0.76767	-7.75204	9.04E-15	3.19E-12	FBN2	2201	fibrillin 2
ENSG0000	372.0362	2.229155	0.288623	7.723427	1.13E-14	3.92E-12	CARD10	29775	caspase recruitment domain family member 10
ENSG0000	218.5395	-2.27844	0.295447	-7.71185	1.24E-14	4.22E-12	RNF152	220441	ring finger protein 152
ENSG0000	99.2046	-2.96535	0.388848	-7.62597	2.42E-14	7.98E-12	TRABD2A	129293	TraB domain containing 2A
ENSG0000	1471.349	1.825311	0.239657	7.616342	2.61E-14	8.46E-12	ADAMTS7	11173	ADAM metallopeptidase with thrombospondin type 1 motif 7
ENSG0000	637.7454	-1.51773	0.199393	-7.61172	2.70E-14	8.63E-12	HS3ST3A1	9955	heparan sulfate-glucosamine 3-sulfotransferase 3A1
ENSG0000	2448.528	-2.55743	0.336399	-7.60237	2.91E-14	9.14E-12	COL7A1	1294	collagen type VII alpha 1 chain
ENSG0000	776.0383	1.827134	0.241055	7.579731	3.46E-14	1.07E-11	FZD4	8322	frizzled class receptor 4
ENSG0000	412.1192	2.120792	0.282339	7.511518	5.84E-14	1.76E-11	DCLK1	9201	doublecortin like kinase 1
ENSG0000	15293.91	2.116071	0.283098	7.474702	7.74E-14	2.29E-11	IGFBP5	3488	insulin like growth factor binding protein 5
ENSG0000	57.10524	-5.80131	0.780605	-7.43182	1.07E-13	3.13E-11	COL23A1	91522	collagen type XXIII alpha 1 chain
ENSG0000	7247.117	2.431929	0.327976	7.41495	1.22E-13	3.50E-11	CYP1B1	1545	cytochrome P450 family 1 subfamily B member 1
ENSG0000	95.04179	-4.34854	0.58803	-7.3951	1.41E-13	4.01E-11	FLRT3	23767	fibronectin leucine rich transmembrane protein 3
ENSG0000	154.8379	4.101987	0.55528	7.387247	1.50E-13	4.19E-11	CACNB4	785	calcium voltage-gated channel auxiliary subunit beta 4
ENSG0000	42.26215	5.813064	0.796783	7.295672	2.97E-13	8.14E-11	PKNOX2	63876	PBX/knotted 1 homeobox 2
ENSG0000	143.2805	1.666585	0.228458	7.294929	2.99E-13	8.14E-11	ADAMTS7	400406	ADAMTS7 pseudogene 3
ENSG0000	212.8903	3.406992	0.467811	7.28284	3.27E-13	8.79E-11	CSTA	1475	cystatin A
ENSG0000	1081.757	2.104898	0.290045	7.257132	3.95E-13	1.05E-10	CCPG1	9236	cell cycle progression 1
ENSG0000	916.0438	-2.68217	0.370334	-7.24257	4.40E-13	1.15E-10	SLC9A3R2	9351	SLC9A3 regulator 2
ENSG0000	613.3747	4.465192	0.616919	7.237887	4.56E-13	1.18E-10	SRGN	5552	serglycin
ENSG0000	61.99605	3.04116	0.421401	7.216787	5.32E-13	1.36E-10	CYP1B1-AS	285154	CYP1B1 antisense RNA 1
ENSG0000	52.18038	-8.15146	1.13466	-7.18406	6.77E-13	1.71E-10	NA	NA	NA
ENSG0000	1318.327	2.571951	0.358751	7.169188	7.54E-13	1.88E-10	HAPLN3	145864	hyaluronan and proteoglycan link protein 3
ENSG0000	421.2785	4.27063	0.602808	7.084561	1.39E-12	3.43E-10	NTRK2	4915	neurotrophic receptor tyrosine kinase 2

ENSG0000	127.4728	6.736148	0.952468	7.072306	1.52E-12	3.71E-10	EYA4	2070	EYA transcriptional coactivator and phosphatase 4
ENSG0000	65.06579	-2.79659	0.395638	-7.06856	1.57E-12	3.76E-10	FERMT3	83706	FERM domain containing kindlin 3
ENSG0000	163.3783	2.918004	0.415328	7.025775	2.13E-12	5.02E-10	ZNF704	619279	zinc finger protein 704
ENSG0000	441.8176	4.122703	0.586843	7.025221	2.14E-12	5.02E-10	TPD52L1	7164	TPD52 like 1
ENSG0000	933.3015	-3.11406	0.443867	-7.01574	2.29E-12	5.31E-10	CHN1	1123	chimerin 1
ENSG0000	181.0131	6.849562	0.978066	7.00317	2.50E-12	5.74E-10	EPHA5	2044	EPH receptor A5
ENSG0000	928.0519	-4.18191	0.603386	-6.93074	4.19E-12	9.50E-10	PTN	5764	pleiotrophin
ENSG0000	29.03387	-4.13981	0.601042	-6.88772	5.67E-12	1.27E-09	RIMS2	9699	regulating synaptic membrane exocytosis 2
ENSG0000	660.9701	4.328983	0.628731	6.885268	5.77E-12	1.28E-09	DEPTOR	64798	DEP domain containing MTOR interacting protein
ENSG0000	187.9547	1.60085	0.234747	6.819471	9.14E-12	1.99E-09	LINC01547	84536	long intergenic non-protein coding RNA 1547
ENSG0000	42.83207	-3.72522	0.548456	-6.79219	1.10E-11	2.37E-09	CARD16	114769	caspase recruitment domain family member 16
ENSG0000	3319.275	-3.47838	0.514104	-6.7659	1.32E-11	2.82E-09	TBX3	6926	T-box transcription factor 3
ENSG0000	41.14699	-4.39775	0.651672	-6.7484	1.49E-11	3.15E-09	TRIM55	84675	tripartite motif containing 55
ENSG0000	7271.508	2.679351	0.397402	6.742162	1.56E-11	3.25E-09	IGFBP7	3490	insulin like growth factor binding protein 7
ENSG0000	25776.1	1.725021	0.257955	6.687282	2.27E-11	4.69E-09	TXNRD1	7296	thioredoxin reductase 1
ENSG0000	52.20727	-5.411	0.809477	-6.68457	2.32E-11	4.73E-09	CES1P1	51716	carboxylesterase 1 pseudogene 1
ENSG0000	332.0405	3.476621	0.520615	6.677918	2.42E-11	4.90E-09	SAMD12	401474	sterile alpha motif domain containing 12
ENSG0000	6245.306	1.736451	0.26027	6.671717	2.53E-11	5.06E-09	C1orf198	84886	chromosome 1 open reading frame 198
ENSG0000	1041.364	8.379112	1.268201	6.607084	3.92E-11	7.76E-09	KRT14	3861	keratin 14
ENSG0000	1256.825	3.332784	0.504519	6.605859	3.95E-11	7.76E-09	MAF	4094	MAF bZIP transcription factor
ENSG0000	41.1757	8.027776	1.220513	6.57738	4.79E-11	9.32E-09	IL26	55801	interleukin 26
ENSG0000	1080.699	1.700739	0.259303	6.558894	5.42E-11	1.04E-08	ADM2	79924	adrenomedullin 2
ENSG0000	73.02492	3.169573	0.484766	6.538357	6.22E-11	1.19E-08	HTR2A	3356	5-hydroxytryptamine receptor 2A
ENSG0000	593.969	-2.03176	0.310807	-6.53705	6.27E-11	1.19E-08	SKAP2	8935	src kinase associated phosphoprotein 2
ENSG0000	272.7314	-2.83737	0.434905	-6.52412	6.84E-11	1.28E-08	PRKAR2B	5577	protein kinase cAMP-dependent type II regulatory sub unit beta
ENSG0000	145.8785	-2.86107	0.440084	-6.50119	7.97E-11	1.48E-08	SEMA6D	80031	semaphorin 6D
ENSG0000	873.9143	-3.56973	0.549445	-6.49696	8.20E-11	1.51E-08	TNFRSF21	27242	TNF receptor superfamily member 21
ENSG0000	10160.56	2.215229	0.342544	6.466986	1.00E-10	1.82E-08	CDH13	1012	cadherin 13
ENSG0000	1974.601	-8.32407	1.291773	-6.44391	1.16E-10	2.09E-08	APCDD1	147495	APC down-regulated 1
ENSG0000	66.09372	-5.93499	0.921385	-6.44138	1.18E-10	2.10E-08	GAL	51083	galanin and GMAP prepropeptide
ENSG0000	424.1536	2.544207	0.395246	6.437024	1.22E-10	2.15E-08	LINC01133	1.01E+08	long intergenic non-protein coding RNA 1133
ENSG0000	58.6884	5.559077	0.86499	6.426754	1.30E-10	2.28E-08	KRT31	3881	keratin 31
ENSG0000	273.6245	3.376971	0.526741	6.411065	1.45E-10	2.50E-08	DMD	1756	dystrophin
ENSG0000	1152.495	-2.15135	0.335791	-6.4068	1.49E-10	2.55E-08	TBX5-AS1	255480	TBX5 antisense RNA 1
ENSG0000	35.49623	-3.30352	0.518775	-6.36793	1.92E-10	3.26E-08	LTK	4058	leukocyte receptor tyrosine kinase
ENSG0000	801.6303	2.856495	0.451641	6.324698	2.54E-10	4.21E-08	DEPP1	11067	DEPP1 autophagy regulator
ENSG0000	39.26634	7.307678	1.157831	6.311522	2.76E-10	4.55E-08	PART1	25859	prostate androgen-regulated transcript 1
ENSG0000	170.0363	4.560258	0.723847	6.300028	2.98E-10	4.82E-08	STEAP4	79689	STEAP4 metalloreductase
ENSG0000	790.125	-2.85002	0.455085	-6.26261	3.79E-10	6.06E-08	GALNT6	11226	polypeptide N-acetylgalactosaminyltransferase 6
ENSG0000	39.36002	3.834232	0.612288	6.262141	3.80E-10	6.06E-08	CDH1	999	cadherin 1
ENSG0000	36.69955	4.887848	0.783276	6.240265	4.37E-10	6.92E-08	NA	NA	NA
ENSG0000	2152.671	1.954225	0.31389	6.22583	4.79E-10	7.53E-08	COL11A1	1301	collagen type XI alpha 1 chain
ENSG0000	307.1557	4.11362	0.661069	6.222676	4.89E-10	7.62E-08	H19	283120	H19 imprinted maternally expressed transcript
ENSG0000	277.7374	-3.4415	0.553475	-6.21799	5.04E-10	7.79E-08	DIRAS3	9077	DIRAS family GTPase 3
ENSG0000	33.72545	-7.57511	1.220713	-6.20548	5.45E-10	8.38E-08	NA	NA	NA
ENSG0000	85.41302	-8.20448	1.323459	-6.19927	5.67E-10	8.65E-08	IRX1	79192	iroquois homeobox 1
ENSG0000	57.598	-6.5998	1.065747	-6.19265	5.92E-10	8.95E-08	SOX11	6664	SRY-box transcription factor 11
ENSG0000	38.61588	4.405136	0.712412	6.183409	6.27E-10	9.42E-08	GPR88	54112	G protein-coupled receptor 88
ENSG0000	604.1987	5.591058	0.904585	6.180797	6.38E-10	9.51E-08	CCKAR	886	cholecystokinin A receptor
ENSG0000	59.64424	-3.72909	0.604819	-6.16564	7.02E-10	1.04E-07	HOXA5	3202	homeobox A5
ENSG0000	177.8521	3.19618	0.524481	6.09399	1.10E-09	1.61E-07	NALCN	259232	sodium leak channel, non-selective
ENSG0000	96.87807	4.744715	0.780386	6.079956	1.20E-09	1.74E-07	SCARA5	286133	scavenger receptor class A member 5
ENSG0000	327.9095	-1.78535	0.293988	-6.07285	1.26E-09	1.81E-07	STXBP6	29091	syntaxin binding protein 6
ENSG0000	128.2124	5.258488	0.869067	6.050731	1.44E-09	2.06E-07	SLC16A9	220963	solute carrier family 16 member 9
ENSG0000	56.92481	6.713645	1.110212	6.047171	1.47E-09	2.09E-07	KRT16P3	644945	keratin 16 pseudogene 3
ENSG0000	161.884	2.543268	0.421799	6.029574	1.64E-09	2.28E-07	CYP19A1	1588	cytochrome P450 family 19 subfamily A member 1
ENSG0000	191.9901	4.094366	0.679389	6.026545	1.68E-09	2.31E-07	KCTD16	57528	potassium channel tetramerization domain containing 16
ENSG0000	72.03205	-7.33273	1.217059	-6.02496	1.69E-09	2.32E-07	NA	NA	NA
ENSG0000	2215.245	3.77613	0.627197	6.020642	1.74E-09	2.37E-07	NTN4	59277	netrin 4
ENSG0000	3843.9	8.636338	1.441841	5.989799	2.10E-09	2.82E-07	SFRP4	6424	secreted frizzled related protein 4
ENSG0000	222.0382	-1.87193	0.312583	-5.98858	2.12E-09	2.82E-07	MBNL3	55796	muscleblind like splicing regulator 3
ENSG0000	31.73182	3.433544	0.573415	5.987893	2.13E-09	2.82E-07	TP63	8626	tumor protein p63
ENSG0000	112.0544	5.986293	1.000081	5.98581	2.15E-09	2.84E-07	KRT33B	3884	keratin 33B
ENSG0000	304.2949	-5.34262	0.892761	-5.98438	2.17E-09	2.84E-07	TRMT9B	57604	tRNA methyltransferase 9B (putative)
ENSG0000	165.4752	1.663684	0.278104	5.98223	2.20E-09	2.86E-07	GSAP	54103	gamma-secretase activating protein
ENSG0000	36.67157	3.088749	0.516465	5.980554	2.22E-09	2.86E-07	LINC02015	1.03E+08	long intergenic non-protein coding RNA 2015
ENSG0000	94.32653	2.058512	0.344207	5.98045	2.23E-09	2.86E-07	LGALSL	29094	galectin like
ENSG0000	50.97731	6.202453	1.037499	5.978271	2.26E-09	2.88E-07	CHRDL2	25884	chordin like 2
ENSG0000	23.40712	6.844448	1.149223	5.955718	2.59E-09	3.27E-07	NA	NA	NA
ENSG0000	84.73215	6.969881	1.170844	5.952868	2.63E-09	3.30E-07	CDH18	1016	cadherin 18
ENSG0000	21210.38	1.83594	0.308951	5.942492	2.81E-09	3.50E-07	ADAMTS1	9510	ADAM metallopeptidase with thrombospondin type 1 motif 1
ENSG0000	19.44536	-4.0583	0.686763	-5.90932	3.44E-09	4.23E-07	NA	NA	NA
ENSG0000	1441.356	2.832187	0.480915	5.889161	3.88E-09	4.73E-07	FNDC1	84624	fibronectin type III domain containing 1
ENSG0000	39.64837	3.92049	0.665759	5.888751	3.89E-09	4.73E-07	NA	NA	NA
ENSG0000	1891.802	2.915523	0.495962	5.878522	4.14E-09	5.00E-07	GFRA1	2674	GDNF family receptor alpha 1
ENSG0000	136.0545	2.716025	0.462339	5.874531	4.24E-09	5.07E-07	SLC2A5	6518	solute carrier family 2 member 5
ENSG0000	30.48444	-5.35075	0.914884	-5.84856	4.96E-09	5.89E-07	NA	NA	NA

ENSG0000	76.70697	6.033985	1.033843	5.836464	5.33E-09	6.30E-07	LINC01018	255167	long intergenic non-protein coding RNA 1018
ENSG0000	21.52505	-5.29856	0.909122	-5.82821	5.60E-09	6.58E-07	P2RY1	5028	purinergic receptor P2Y1
ENSG0000	23.67829	4.232144	0.728993	5.80547	6.42E-09	7.45E-07	LINC01750	643355	long intergenic non-protein coding RNA 1750
ENSG0000	74.72837	5.809238	1.000969	5.803617	6.49E-09	7.49E-07	HRH2	3274	histamine receptor H2
ENSG0000	81.27146	1.64718	0.284184	5.796176	6.78E-09	7.79E-07	ZFHX4-AS1	1E+08	ZFHX4 antisense RNA 1
ENSG0000	111.8538	3.267732	0.564669	5.786987	7.17E-09	8.09E-07	CCND2	894	cyclin D2
ENSG0000	1695.706	1.699391	0.2939	5.782204	7.37E-09	8.28E-07	ARL4C	10123	ADP ribosylation factor like GTPase 4C
ENSG0000	304.8865	2.267932	0.393704	5.760506	8.39E-09	9.36E-07	FUCA1	2517	alpha-L-fucosidase 1
ENSG0000	150.8549	3.178299	0.552601	5.751531	8.84E-09	9.82E-07	PCSK1	5122	proprotein convertase subtilisin/kexin type 1
ENSG0000	102.9551	-5.81356	1.013757	-5.73467	9.77E-09	1.07E-06	ANO4	121601	anoctamin 4
ENSG0000	60.32538	4.159232	0.725916	5.729632	1.01E-08	1.09E-06	KLHDC7B	113730	kelch domain containing 7B
ENSG0000	29.73085	4.011383	0.700457	5.726808	1.02E-08	1.11E-06	IGF1	3479	insulin like growth factor 1
ENSG0000	17155.13	1.524845	0.267212	5.706498	1.15E-08	1.24E-06	GJA1	2697	gap junction protein alpha 1
ENSG0000	31.80065	4.103646	0.720936	5.69211	1.25E-08	1.34E-06	CCDC110	256309	coiled-coil domain containing 110
ENSG0000	250.866	-2.94038	0.516937	-5.68809	1.28E-08	1.37E-06	TOX2	84969	TOX high mobility group box family member 2
ENSG0000	140.5113	2.422745	0.426056	5.686444	1.30E-08	1.37E-06	HMGA2-A	1E+08	HMGA2 antisense RNA 1
ENSG0000	390.8244	-3.76617	0.663618	-5.67521	1.39E-08	1.44E-06	CASP1	834	caspase 1
ENSG0000	47.14542	2.116269	0.373065	5.672656	1.41E-08	1.46E-06	ADAMTS7	390660	ADAMTS7 pseudogene 1
ENSG0000	57.29886	4.757984	0.8393	5.66899	1.44E-08	1.47E-06	PADI2	11240	peptidyl arginine deiminase 2
ENSG0000	179.5165	3.525234	0.622214	5.665631	1.46E-08	1.50E-06	NA	NA	NA
ENSG0000	291.2887	2.585143	0.457076	5.655823	1.55E-08	1.57E-06	ASPHD2	57168	aspartate beta-hydroxylase domain containing 2
ENSG0000	46.7031	-4.05582	0.71747	-5.65295	1.58E-08	1.59E-06	APBA2	321	amyloid beta precursor protein binding family A mem ber 2
ENSG0000	70.09104	3.120444	0.552852	5.644269	1.66E-08	1.66E-06	KRTAP1-1	81851	keratin associated protein 1-1
ENSG0000	26.35013	-4.03713	0.717489	-5.62674	1.84E-08	1.82E-06	ST8SIA5	29906	ST8 alpha-N-acetyl-neuraminide alpha-2,8-sialyltransf erase 5
ENSG0000	3294.453	2.110241	0.375997	5.612383	2.00E-08	1.97E-06	GRAMD2B	65983	GRAM domain containing 2B
ENSG0000	869.8808	-2.39121	0.427285	-5.59629	2.19E-08	2.15E-06	GNG2	54331	G protein subunit gamma 2
ENSG0000	43.69225	3.677452	0.657618	5.592081	2.24E-08	2.18E-06	GALNT13	114805	polypeptide N-acetylgalactosaminyltransferase 13
ENSG0000	116.7767	1.549452	0.277638	5.580831	2.39E-08	2.32E-06	RFLNA	144347	refilin A
ENSG0000	79.52821	2.601715	0.466309	5.579374	2.41E-08	2.33E-06	VLDLR-AS1	401491	VLDLR antisense RNA 1
ENSG0000	898.338	1.646423	0.295195	5.577416	2.44E-08	2.34E-06	COBLL1	22837	cordon-bleu WH2 repeat protein like 1
ENSG0000	368.8601	3.246669	0.582535	5.573343	2.50E-08	2.39E-06	GATA6	2627	GATA binding protein 6
ENSG0000	39.42904	-4.90894	0.8813	-5.57011	2.55E-08	2.42E-06	HOXA1	3198	homeobox A1
ENSG0000	62.39832	-3.84566	0.69335	-5.5465	2.91E-08	2.74E-06	WNT7B	7477	Wnt family member 7B
ENSG0000	27.00907	4.223776	0.76167	5.545413	2.93E-08	2.75E-06	APOA1	335	apolipoprotein A1
ENSG0000	664.2147	2.679144	0.483915	5.5364	3.09E-08	2.88E-06	GDF5	8200	growth differentiation factor 5
ENSG0000	50.98487	5.890146	1.06466	5.532419	3.16E-08	2.93E-06	TMEM178	1.01E+08	transmembrane protein 178B
ENSG0000	2156.026	2.47648	0.448355	5.523484	3.32E-08	3.07E-06	LACC1	144811	laccase domain containing 1
ENSG0000	319.7704	2.089107	0.378679	5.516826	3.45E-08	3.16E-06	NHSL1	57224	NHS like 1
ENSG0000	1388.445	-1.70441	0.308975	-5.51633	3.46E-08	3.16E-06	GNG11	2791	G protein subunit gamma 11
ENSG0000	1839.989	3.561075	0.649254	5.484876	4.14E-08	3.73E-06	MEST	4232	mesoderm specific transcript
ENSG0000	448.0038	2.06584	0.378145	5.463091	4.68E-08	4.17E-06	NA	NA	NA
ENSG0000	8364.688	8.644128	1.585059	5.453505	4.94E-08	4.39E-06	ACAN	176	aggrecan
ENSG0000	18012.64	-3.88667	0.714611	-5.43886	5.36E-08	4.74E-06	MXRA5	25878	matrix remodeling associated 5
ENSG0000	264.9256	-8.69776	1.602947	-5.4261	5.76E-08	5.03E-06	GRIK2	2898	glutamate ionotropic receptor kainate type subunit 2
ENSG0000	160.828	-4.53594	0.837032	-5.41907	5.99E-08	5.21E-06	COL6A6	131873	collagen type VI alpha 6 chain
ENSG0000	184.5698	-5.25206	0.972234	-5.40205	6.59E-08	5.67E-06	MAP2K6	5608	mitogen-activated protein kinase kinase 6

Supp Table 2 DDC vs NS comparison

ENSG	baseMean	log2FoldC	lfcSE	stat	pvalue	padj	symbol	entrez	genename
ENSG0000	1751.458	-3.99209	0.182657	-21.8557	6.86E-106	1.80E-101	JAM2	58494	junctional adhesion molecule 2
ENSG0000	8139.154	-2.95224	0.178747	-16.5163	2.80E-61	3.68E-57	JUND	3727	JunD proto-oncogene, AP-1 transcription factor subunit
ENSG0000	4536.474	-3.8835	0.237745	-16.3347	5.59E-60	4.89E-56	CSRNP1	64651	cysteine and serine rich nuclear protein 1
ENSG0000	391.7914	-4.68084	0.288902	-16.2021	4.87E-59	3.20E-55	TNFSF9	8744	TNF superfamily member 9
ENSG0000	13622.91	-4.41895	0.29259	-15.1029	1.55E-51	8.14E-48	GADD45B	4616	growth arrest and DNA damage inducible beta
ENSG0000	424.3872	2.005845	0.133761	14.99568	7.84E-51	3.43E-47	MRTFB	57496	myocardin related transcription factor B
ENSG0000	393.3528	-5.98573	0.399742	-14.974	1.09E-50	4.08E-47	HES4	57801	hes family bHLH transcription factor 4
ENSG0000	13590.97	-5.35951	0.369699	-14.497	1.27E-47	3.70E-44	BHLHE40	8553	basic helix-loop-helix family member e40
ENSG0000	4325.254	-3.94389	0.277538	-14.2103	7.91E-46	2.08E-42	PER1	5187	period circadian regulator 1
ENSG0000	100532.5	-2.10177	0.148745	-14.13	2.48E-45	5.92E-42	ND2	4536	NADH dehydrogenase subunit 2
ENSG0000	2633.898	-3.08842	0.221583	-13.938	3.72E-44	8.15E-41	NA	NA	NA
ENSG0000	922.621	-4.51819	0.324937	-13.9048	5.92E-44	1.20E-40	DAPK1	1612	death associated protein kinase 1
ENSG0000	7550.216	-2.63214	0.196139	-13.4198	4.63E-41	8.69E-38	MIDN	90007	midnolin
ENSG0000	6378.852	-4.49823	0.342968	-13.1156	2.68E-39	4.14E-36	HES1	3280	hes family bHLH transcription factor 1
ENSG0000	1075.316	-1.57721	0.125248	-12.5927	2.32E-36	3.38E-33	SELENOK	58515	selenoprotein K
ENSG0000	146.2949	6.456761	0.517608	12.47423	1.03E-35	1.43E-32	HOXD10	3236	homeobox D10
ENSG0000	1061.686	-6.19903	0.501918	-12.3507	4.83E-35	6.34E-32	C11orf96	387763	chromosome 11 open reading frame 96
ENSG0000	1932.567	1.876146	0.153224	12.2445	1.80E-34	2.25E-31	SH3KBP1	30011	SH3 domain containing kinase binding protein 1
ENSG0000	8527.247	-2.86766	0.235011	-12.2023	3.02E-34	3.61E-31	KLF10	7071	KLF transcription factor 10
ENSG0000	87.96214	5.844008	0.485298	12.0421	2.13E-33	2.44E-30	GALNT18	374378	polypeptide N-acetylgalactosaminyltransferase 18
ENSG0000	294.9143	6.777534	0.564817	11.99953	3.57E-33	3.91E-30	VAT1L	57687	vesicle amine transport 1 like
ENSG0000	267.5419	-4.25597	0.364698	-11.6698	1.82E-31	1.84E-28	LIMS3	96626	LIM zinc finger domain containing 3
ENSG0000	259.4226	-4.25694	0.370333	-11.4949	1.40E-30	1.27E-27	LIMS4	1E+08	LIM zinc finger domain containing 4
ENSG0000	338.8982	-6.79697	0.595277	-11.4182	3.39E-30	2.97E-27	NA	NA	NA
ENSG0000	65.65142	5.880763	0.515235	11.41375	3.57E-30	3.02E-27	HOXD11	3237	homeobox D11
ENSG0000	25511.49	-1.91444	0.16782	-11.4077	3.83E-30	3.14E-27	ND4L	4539	NADH dehydrogenase subunit 4L
ENSG0000	3197.946	1.887737	0.165715	11.39146	4.61E-30	3.60E-27	PTPN13	5783	protein tyrosine phosphatase non-receptor type 13
ENSG0000	46344.4	-2.31991	0.203668	-11.3906	4.66E-30	3.60E-27	MTND2P2	1.01E+08	MT-ND2 pseudogene 28
ENSG0000	2541.964	-2.41436	0.21518	-11.2202	3.24E-29	2.43E-26	MAFF	23764	MAF bZIP transcription factor F
ENSG0000	77.03513	-3.62918	0.324131	-11.1966	4.23E-29	3.09E-26	NA	NA	NA
ENSG0000	1367.199	-1.6113	0.146106	-11.0283	2.79E-28	1.98E-25	NA	NA	NA
ENSG0000	2186.721	1.999115	0.181924	10.98876	4.33E-28	2.99E-25	ITPRID2	6744	ITPR interacting domain containing 2
ENSG0000	100.425	-3.69109	0.336626	-10.965	5.63E-28	3.79E-25	NA	NA	NA
ENSG0000	100.5881	-3.78383	0.351766	-10.7567	5.51E-27	3.53E-24	NA	NA	NA
ENSG0000	2701.762	-1.62242	0.151201	-10.7303	7.34E-27	4.59E-24	DDAH2	23564	dimethylarginine dimethylaminohydrolase 2
ENSG0000	54.7552	-4.75816	0.445771	-10.674	1.35E-26	8.04E-24	HOXC-AS2	1.01E+08	HOXC cluster antisense RNA 2
ENSG0000	228.1435	1.577099	0.148116	10.6477	1.79E-26	1.04E-23	GSPT2	23708	G1 to S phase transition 2
ENSG0000	401.2329	-4.4021	0.414703	-10.6151	2.54E-26	1.45E-23	EML6	400954	EMAP like 6
ENSG0000	359.8155	6.078262	0.576909	10.53591	5.90E-26	3.30E-23	HOXA13	3209	homeobox A13
ENSG0000	1243.021	1.661063	0.157772	10.52824	6.40E-26	3.50E-23	ZNF395	55893	zinc finger protein 395
ENSG0000	596.9496	-1.77546	0.170853	-10.3917	2.70E-25	1.45E-22	ZNF408	79797	zinc finger protein 408
ENSG0000	3129.744	-3.68871	0.360252	-10.2393	1.32E-24	6.81E-22	ZC3H12A	80149	zinc finger CCCH-type containing 12A
ENSG0000	207.4425	-10.9559	1.07403	-10.2008	1.97E-24	9.93E-22	LEP	3952	leptin
ENSG0000	1474.826	-5.21061	0.512295	-10.1711	2.67E-24	1.29E-21	ATF3	467	activating transcription factor 3
ENSG0000	1048.174	1.671962	0.164402	10.16996	2.70E-24	1.29E-21	PTGR3	284273	prostaglandin reductase 3
ENSG0000	99.36353	-5.55874	0.555998	-9.99778	1.56E-23	7.18E-21	NA	NA	NA
ENSG0000	417.2829	-5.58436	0.560241	-9.96778	2.11E-23	9.55E-21	USP2	9099	ubiquitin specific peptidase 2
ENSG0000	1508.093	-4.75232	0.476899	-9.96504	2.17E-23	9.65E-21	LOC10272	1.03E+08	salt inducible kinase 1B (putative)
ENSG0000	1365.287	-4.70383	0.472509	-9.955	2.40E-23	1.05E-20	SIK1	150094	salt inducible kinase 1
ENSG0000	479.169	-2.09912	0.211791	-9.9113	3.72E-23	1.57E-20	TUFT1	7286	tuftelin 1
ENSG0000	477.1729	-1.89239	0.190956	-9.91008	3.76E-23	1.57E-20	H1-2	3006	H1.2 linker histone, cluster member
ENSG0000	7047.515	2.231672	0.226451	9.854998	6.52E-23	2.68E-20	CD109	135228	CD109 molecule
ENSG0000	358.6499	-8.43033	0.855739	-9.85152	6.75E-23	2.73E-20	MYH1	4619	myosin heavy chain 1
ENSG0000	748.7	1.769018	0.179617	9.848814	6.94E-23	2.76E-20	PPARA	5465	peroxisome proliferator activated receptor alpha
ENSG0000	1275.773	-3.14592	0.323036	-9.73861	2.06E-22	8.09E-20	MAP3K8	1326	mitogen-activated protein kinase kinase kinase 8
ENSG0000	191.8913	1.728914	0.177595	9.735126	2.14E-22	8.25E-20	PDP2	57546	pyruvate dehydrogenase phosphatase catalytic subunit 2
ENSG0000	1519.447	-2.31635	0.238009	-9.73221	2.20E-22	8.32E-20	GDPD5	81544	glycerophosphodiester phosphodiesterase domain contain ng 5
ENSG0000	1337.994	-1.98349	0.203827	-9.73124	2.22E-22	8.32E-20	NA	NA	NA
ENSG0000	43192.03	-1.55111	0.159554	-9.72153	2.44E-22	9.03E-20	MTATP6P	1.06E+08	MT-ATP6 pseudogene 1
ENSG0000	116.8718	-10.1288	1.047503	-9.66944	4.07E-22	1.46E-19	HOXB8	3218	homeobox B8
ENSG0000	113.7697	-3.48485	0.360472	-9.66745	4.15E-22	1.47E-19	NA	NA	NA
ENSG0000	102.3169	-3.53384	0.367122	-9.62578	6.22E-22	2.18E-19	NA	NA	NA
ENSG0000	225837.6	-1.70149	0.17703	-9.61128	7.17E-22	2.48E-19	ND4	4538	NADH dehydrogenase subunit 4
ENSG0000	996.5017	-6.06082	0.63105	-9.60435	7.66E-22	2.61E-19	PCSK9	255738	proprotein convertase subtilisin/kexin type 9
ENSG0000	128355.5	-2.14083	0.223384	-9.58363	9.37E-22	3.16E-19	CYTB	4519	cytochrome b
ENSG0000	129.3665	-6.12554	0.641381	-9.55055	1.29E-21	4.18E-19	EXPH5	23086	exophilin 5
ENSG0000	2154.908	-2.73588	0.287122	-9.52863	1.59E-21	5.10E-19	LIMS2	55679	LIM zinc finger domain containing 2
ENSG0000	105.8647	-2.09924	0.220839	-9.50576	1.99E-21	6.21E-19	MMP15	4324	matrix metallopeptidase 15
ENSG0000	153.4485	-1.52603	0.16151	-9.44853	3.44E-21	1.06E-18	NA	NA	NA
ENSG0000	370.6283	-2.76478	0.294297	-9.39452	5.75E-21	1.71E-18	HSD17B14	51171	hydroxysteroid 17-beta dehydrogenase 14
ENSG0000	154.0538	-4.64282	0.494246	-9.39375	5.79E-21	1.71E-18	TRPC6	7225	transient receptor potential cation channel subfamily C m ember 6
ENSG0000	224.9752	1.696886	0.180719	9.389633	6.02E-21	1.76E-18	KBTBD6	89890	kelch repeat and BTB domain containing 6
ENSG0000	614.5936	-3.3338	0.355213	-9.38534	6.27E-21	1.81E-18	KIT	3815	KIT proto-oncogene, receptor tyrosine kinase
ENSG0000	961.1958	3.658463	0.391816	9.337192	9.89E-21	2.82E-18	MATN2	4147	matrilin 2
ENSG0000	700.6564	-2.09847	0.226111	-9.28068	1.68E-20	4.76E-18	DHRS7B	25979	dehydrogenase/reductase 7B
ENSG0000	1429.274	3.513165	0.380688	9.228468	2.75E-20	7.37E-18	CA12	771	carbonic anhydrase 12
ENSG0000	33.91774	-3.41513	0.370711	-9.21239	3.19E-20	8.38E-18	BHLHE40-	1.01E+08	BHLHE40 antisense RNA 1
ENSG0000	938.5063	2.653177	0.288591	9.193552	3.80E-20	9.88E-18	EHD3	30845	EH domain containing 3
ENSG0000	196.1425	-1.7539	0.1914	-9.16352	5.02E-20	1.27E-17	C15orf61	145853	chromosome 15 open reading frame 61
ENSG0000	263.4157	-9.85328	1.080103	-9.12253	7.34E-20	1.82E-17	HOXB-AS3	404266	HOXB cluster antisense RNA 3
ENSG0000	4006.734	-3.2481	0.3561	-9.12132	7.42E-20	1.82E-17	PIM1	5292	Pim-1 proto-oncogene, serine/threonine kinase
ENSG0000	59.53254	3.202532	0.35161	9.108189	8.38E-20	2.04E-17	SOX8	30812	SRY-box transcription factor 8

ENSG0000	50.22782	4.580277	0.503179	9.102678	8.81E-20	2.12E-17	NA	NA	NA
ENSG0000	875.135	-1.63304	0.180501	-9.04729	1.47E-19	3.50E-17	PTS	5805	6-pyruvoyltetrahydropterin synthase
ENSG0000	1832.423	-1.60833	0.178844	-8.99289	2.41E-19	5.60E-17	NEU1	4758	neuraminidase 1
ENSG0000	225.8076	-10.3567	1.1534	-8.97932	2.72E-19	6.22E-17	HES5	388585	hes family bHLH transcription factor 5
ENSG0000	660.8992	2.409133	0.269141	8.951198	3.52E-19	7.96E-17	NHSL2	340527	NHS like 2
ENSG0000	858.7559	-5.7876	0.646999	-8.9453	3.71E-19	8.33E-17	INMT	11185	indolethylamine N-methyltransferase
ENSG0000	2021.079	-3.67837	0.411325	-8.94272	3.80E-19	8.45E-17	TRIB1	10221	tribbles pseudokinase 1
ENSG0000	48.32817	-3.54978	0.397895	-8.92139	4.60E-19	1.02E-16	NA	NA	NA
ENSG0000	73.77881	3.967342	0.446	8.895385	5.82E-19	1.27E-16	ICAM5	7087	intercellular adhesion molecule 5
ENSG0000	434.6675	2.758944	0.312716	8.822534	1.12E-18	2.37E-16	INSYN2B	1E+08	inhibitory synaptic factor family member 2B
ENSG0000	1871.912	-3.49008	0.397987	-8.76934	1.80E-18	3.75E-16	KLF2	10365	KLF transcription factor 2
ENSG0000	7619.416	-2.09029	0.238528	-8.7633	1.90E-18	3.92E-16	PPP1R15A	23645	protein phosphatase 1 regulatory subunit 15A
ENSG0000	21924.35	1.542127	0.176176	8.753347	2.07E-18	4.22E-16	CD44	960	CD44 molecule (Indian blood group)
ENSG0000	2927.289	-12.2192	1.402333	-8.71347	2.95E-18	5.95E-16	SMOC2	64094	SPARC related modular calcium binding 2
ENSG0000	88.26492	8.749609	1.004958	8.706443	3.14E-18	6.29E-16	SHOX	6473	short stature homeobox
ENSG0000	882.9998	-2.09093	0.240558	-8.69202	3.56E-18	6.98E-16	JAG1	182	jagged canonical Notch ligand 1
ENSG0000	366.7137	-3.36008	0.388711	-8.64415	5.42E-18	1.03E-15	FGF14	2259	fibroblast growth factor 14
ENSG0000	108080.9	-1.53005	0.178769	-8.55883	1.14E-17	2.08E-15	ATP6	4508	ATP synthase F0 subunit 6
ENSG0000	267.4607	-1.72938	0.202892	-8.52366	1.55E-17	2.76E-15	NA	NA	NA
ENSG0000	223.3337	-3.44151	0.405006	-8.49745	1.94E-17	3.42E-15	IDI2-AS1	55853	IDI2 antisense RNA 1
ENSG0000	1945.535	1.501736	0.178343	8.420492	3.75E-17	6.43E-15	CNOT1	23019	CCR4-NOT transcription complex subunit 1
ENSG0000	6476.403	-2.75457	0.327263	-8.417	3.86E-17	6.55E-15	INSIG1	3638	insulin induced gene 1
ENSG0000	42.09023	6.435995	0.766282	8.398992	4.50E-17	7.49E-15	COL23A1	91522	collagen type XXIII alpha 1 chain
ENSG0000	477697.2	-1.68063	0.201394	-8.34499	7.12E-17	1.14E-14	COX1	4512	cytochrome c oxidase subunit I
ENSG0000	37.5955	4.499105	0.539517	8.339133	7.48E-17	1.19E-14	SORL1	6653	sortilin related receptor 1
ENSG0000	342.8852	-2.94786	0.353947	-8.32854	8.18E-17	1.29E-14	TMEM171	134285	transmembrane protein 171
ENSG0000	241.654	1.584349	0.191048	8.292934	1.10E-16	1.73E-14	PTER	9317	phosphotriesterase related
ENSG0000	952.0344	-1.50371	0.181399	-8.28951	1.14E-16	1.77E-14	THAP9-AS	1E+08	THAP9 antisense RNA 1
ENSG0000	156.0573	1.662024	0.201085	8.265263	1.39E-16	2.14E-14	ZBTB39	9880	zinc finger and BTB domain containing 39
ENSG0000	274.1051	4.911045	0.594897	8.255291	1.52E-16	2.31E-14	AFF2	2334	ALF transcription elongation factor 2
ENSG0000	948.5801	-1.61091	0.195182	-8.2534	1.54E-16	2.34E-14	SLC25A25	114789	solute carrier family 25 member 25
ENSG0000	249.3369	-1.53809	0.18658	-8.24363	1.67E-16	2.52E-14	FAM89B	23625	family with sequence similarity 89 member B
ENSG0000	454.0147	3.984524	0.483527	8.240535	1.71E-16	2.56E-14	TM4SF1	4071	transmembrane 4 L six family member 1
ENSG0000	207.3308	-1.6521	0.204233	-8.08929	6.00E-16	8.66E-14	REC8	9985	REC8 meiotic recombination protein
ENSG0000	1515.75	1.901517	0.23516	8.086071	6.16E-16	8.84E-14	TSHZ1	10194	teashirt zinc finger homeobox 1
ENSG0000	85.0154	-5.33013	0.662046	-8.051	8.21E-16	1.17E-13	NA	NA	NA
ENSG0000	112785.1	1.901834	0.236397	8.045074	8.62E-16	1.22E-13	COL6A3	1293	collagen type VI alpha 3 chain
ENSG0000	39.30015	-4.18459	0.521303	-8.02716	9.98E-16	1.41E-13	NA	NA	NA
ENSG0000	1195.772	-2.01149	0.25096	-8.01517	1.10E-15	1.54E-13	ANK2	287	ankyrin 2
ENSG0000	1537.87	-1.61978	0.202976	-7.98014	1.46E-15	2.01E-13	JMJD6	23210	jumonji domain containing 6, arginine demethylase and lysine hydroxylase
ENSG0000	859.2714	2.498415	0.313175	7.977696	1.49E-15	2.04E-13	TBX5-AS1	255480	TBX5 antisense RNA 1
ENSG0000	33.6731	4.96772	0.626031	7.93526	2.10E-15	2.81E-13	GALNT14	79623	polypeptide N-acetylgalactosaminyltransferase 14
ENSG0000	20.07205	8.059297	1.01606	7.931912	2.16E-15	2.88E-13	HOXD13	3239	homeobox D13
ENSG0000	204.9394	-4.2363	0.535682	-7.90823	2.61E-15	3.44E-13	B3GALT2	8707	beta-1,3-galactosyltransferase 2
ENSG0000	44.62946	-3.84838	0.486664	-7.90767	2.62E-15	3.44E-13	NR3C2	4306	nuclear receptor subfamily 3 group C member 2
ENSG0000	1840.461	-3.23043	0.408934	-7.89964	2.80E-15	3.65E-13	PLAC9	219348	placenta associated 9
ENSG0000	791.6409	-4.90129	0.62073	-7.89601	2.88E-15	3.74E-13	HEY1	23462	hes related family bHLH transcription factor with YRPW motif 1
ENSG0000	566.1145	-3.78913	0.480399	-7.88746	3.08E-15	3.95E-13	RRAD	6236	RRAD, Ras related glycolysis inhibitor and calcium channel regulator
ENSG0000	184.41	2.498058	0.316967	7.881123	3.24E-15	4.14E-13	RHOU	58480	ras homolog family member U
ENSG0000	1301.936	1.751535	0.222283	7.879756	3.28E-15	4.16E-13	NA	NA	NA
ENSG0000	77.9968	-5.06088	0.643282	-7.86729	3.62E-15	4.58E-13	HOXC-AS1	1.01E+08	HOXC cluster antisense RNA 1
ENSG0000	6384.186	-3.38836	0.431382	-7.85465	4.01E-15	5.04E-13	IER3	8870	immediate early response 3
ENSG0000	4929.199	-3.0648	0.390405	-7.8503	4.15E-15	5.19E-13	LMCD1	29995	LIM and cysteine rich domains 1
ENSG0000	158008.8	-1.70157	0.216835	-7.8473	4.25E-15	5.29E-13	MTCO1P1	1.07E+08	MT-CO1 pseudogene 12
ENSG0000	29.81113	-3.86256	0.492384	-7.84461	4.34E-15	5.36E-13	NA	NA	NA
ENSG0000	108832.8	-1.56035	0.199091	-7.83737	4.60E-15	5.65E-13	ND5	4540	NADH dehydrogenase subunit 5
ENSG0000	24569.97	-1.59629	0.203872	-7.82986	4.88E-15	5.94E-13	CDKN1A	1026	cyclin dependent kinase inhibitor 1A
ENSG0000	359.4522	2.292043	0.293092	7.820214	5.27E-15	6.35E-13	THSD4	79875	thrombospondin type 1 domain containing 4
ENSG0000	944.3016	1.681321	0.215063	7.817824	5.37E-15	6.45E-13	TBL1X	6907	transducin beta like 1 X-linked
ENSG0000	1783.413	2.017244	0.258452	7.80509	5.95E-15	7.10E-13	PCDHGC3	5098	protocadherin gamma subfamily C, 3
ENSG0000	1105.516	-1.77991	0.2281	-7.80319	6.04E-15	7.17E-13	TMSB4XP4	7118	TMSB4X pseudogene 4
ENSG0000	124.7077	3.516472	0.452084	7.778363	7.35E-15	8.65E-13	COL6A6	131873	collagen type VI alpha 6 chain
ENSG0000	139.9359	-1.57526	0.202747	-7.76958	7.87E-15	9.19E-13	ZNF433	163059	zinc finger protein 433
ENSG0000	110.7199	-2.72057	0.350158	-7.76957	7.88E-15	9.19E-13	CD14	929	CD14 molecule
ENSG0000	132.7503	-1.58821	0.204515	-7.76575	8.12E-15	9.43E-13	C4orf48	401115	chromosome 4 open reading frame 48
ENSG0000	1964.48	1.962381	0.252763	7.763717	8.25E-15	9.54E-13	SYNJ2	8871	synaptojanin 2
ENSG0000	573.7673	-3.77358	0.489099	-7.71537	1.21E-14	1.39E-12	LINC02381	400043	long intergenic non-protein coding RNA 2381
ENSG0000	3934.514	3.373478	0.437357	7.713333	1.23E-14	1.41E-12	PTGS1	5742	prostaglandin-endoperoxide synthase 1
ENSG0000	35267.53	-5.3219	0.690138	-7.71136	1.24E-14	1.42E-12	FOSB	2354	FosB proto-oncogene, AP-1 transcription factor subunit
ENSG0000	1452.692	-1.53305	0.198824	-7.71059	1.25E-14	1.42E-12	H1-10	8971	H1.10 linker histone
ENSG0000	83526.78	-2.11803	0.274731	-7.70946	1.26E-14	1.43E-12	ND1	4535	NADH dehydrogenase subunit 1
ENSG0000	429.5929	1.627125	0.211154	7.705866	1.30E-14	1.46E-12	CRYBG3	131544	crystallin beta-gamma domain containing 3
ENSG0000	1271.655	-2.83389	0.368284	-7.69485	1.42E-14	1.58E-12	FIBIN	387758	fin bud initiation factor homolog
ENSG0000	140.4432	6.884147	0.894807	7.693444	1.43E-14	1.59E-12	ZFY	7544	zinc finger protein Y-linked
ENSG0000	13287.86	-1.51692	0.197415	-7.68391	1.54E-14	1.70E-12	NA	NA	NA
ENSG0000	501.3535	-2.44957	0.319019	-7.67843	1.61E-14	1.76E-12	SLC19A2	10560	solute carrier family 19 member 2
ENSG0000	869.4195	-1.64742	0.214928	-7.66501	1.79E-14	1.94E-12	PIK3IP1	113791	phosphoinositide-3-kinase interacting protein 1
ENSG0000	963.796	1.69126	0.220677	7.663965	1.80E-14	1.95E-12	PPP2R3A	5523	protein phosphatase 2 regulatory subunit B''alpha
ENSG0000	1215.393	-1.67809	0.219356	-7.65006	2.01E-14	2.15E-12	CASTOR2	729438	cytosolic arginine sensor for mTORC1 subunit 2
ENSG0000	1108.468	1.735694	0.227144	7.641372	2.15E-14	2.28E-12	NA	NA	NA
ENSG0000	32.36498	-3.29186	0.430807	-7.64115	2.15E-14	2.28E-12	KCP	375616	kielin cysteine rich BMP regulator
ENSG0000	448.8958	-3.12055	0.410578	-7.60039	2.95E-14	3.09E-12	CDKN1C	1028	cyclin dependent kinase inhibitor 1C
ENSG0000	64.0959	-9.26173	1.219109	-7.59713	3.03E-14	3.16E-12	NPNT	255743	nephronectin

ENSG0000	588.4189	-3.39005	0.447058	-7.58301	3.38E-14	3.48E-12	CHRD	8646	chordin
ENSG0000	27.64859	-3.73939	0.493264	-7.5809	3.43E-14	3.51E-12	CCDC148	130940	coiled-coil domain containing 148
ENSG0000	31.65399	-5.19311	0.686068	-7.56938	3.75E-14	3.79E-12	LOC10537	1.05E+08	uncharacterized LOC105370047
ENSG0000	105.4978	-2.23587	0.295906	-7.55602	4.16E-14	4.17E-12	DHRS12	79758	dehydrogenase/reductase 12
ENSG0000	17.9985	4.493609	0.594778	7.555102	4.19E-14	4.18E-12	NA	NA	NA
ENSG0000	199.0755	3.25139	0.430651	7.549936	4.35E-14	4.33E-12	PRKAR2B	5577	protein kinase cAMP-dependent type II regulatory subunit beta
ENSG0000	1495.317	-3.01048	0.3997	-7.53185	5.00E-14	4.92E-12	RNA5-8SN	1.1E+08	RNA, 5.8S ribosomal N3
ENSG0000	1494.572	-3.0097	0.399921	-7.52575	5.24E-14	5.12E-12	RNA5-8SN	1.1E+08	RNA, 5.8S ribosomal N2
ENSG0000	1493.937	-3.01154	0.40078	-7.51419	5.73E-14	5.55E-12	RNA5-8SN	1.07E+08	RNA, 5.8S ribosomal N1
ENSG0000	1493.192	-3.01076	0.401001	-7.50812	6.00E-14	5.75E-12	LOC12490	1.25E+08	5.8S ribosomal RNA
ENSG0000	1493.192	-3.01076	0.401001	-7.50812	6.00E-14	5.75E-12	LOC12490	1.25E+08	5.8S ribosomal RNA
ENSG0000	1493.192	-3.01076	0.401001	-7.50812	6.00E-14	5.75E-12	RNA5-8SP	1.1E+08	RNA, 5.8S ribosomal pseudogene 10
ENSG0000	1492.862	-3.01042	0.401074	-7.5059	6.10E-14	5.81E-12	RNA5-8SN	1E+08	RNA, 5.8S ribosomal N5
ENSG0000	1492.862	-3.01042	0.401074	-7.5059	6.10E-14	5.81E-12	RNA5-8SN	1.1E+08	RNA, 5.8S ribosomal N4
ENSG0000	108.7304	6.770845	0.90346	7.49435	6.66E-14	6.32E-12	MYPN	84665	myopalladin
ENSG0000	82.71256	2.091138	0.279763	7.474688	7.74E-14	7.29E-12	FAR2	55711	fatty acyl-CoA reductase 2
ENSG0000	93.24905	8.831182	1.181701	7.473282	7.82E-14	7.31E-12	PPP2R2B	5521	protein phosphatase 2 regulatory subunit Bbeta
ENSG0000	17596.63	-2.96255	0.396503	-7.4717	7.92E-14	7.37E-12	HMOX1	3162	heme oxygenase 1
ENSG0000	1743.851	-2.68902	0.360086	-7.4677	8.16E-14	7.57E-12	CLCF1	23529	cardiotrophin like cytokine factor 1
ENSG0000	1963.6	-2.23115	0.299032	-7.46126	8.57E-14	7.93E-12	ARL5B	221079	ADP ribosylation factor like GTPase 5B
ENSG0000	11162.55	-2.01725	0.270889	-7.44675	9.57E-14	8.75E-12	MTND1P2	1.01E+08	MT-ND1 pseudogene 23
ENSG0000	281.4301	-5.4297	0.72929	-7.44519	9.68E-14	8.83E-12	FGF18	8817	fibroblast growth factor 18
ENSG0000	76.74286	-2.38917	0.322077	-7.41801	1.19E-13	1.07E-11	NA	NA	NA
ENSG0000	106.8736	2.307133	0.3113	7.411292	1.25E-13	1.11E-11	MIR381HG	378881	MIR381 host gene
ENSG0000	88.49612	-3.66841	0.495851	-7.39821	1.38E-13	1.20E-11	SEMA6A	57556	semaphorin 6A
ENSG0000	432.9565	5.538879	0.748917	7.395855	1.41E-13	1.22E-11	TENT5C	54855	terminal nucleotidyltransferase 5C
ENSG0000	43.10068	-5.41779	0.732917	-7.39209	1.45E-13	1.25E-11	ITIH3	3699	inter-alpha-trypsin inhibitor heavy chain 3
ENSG0000	44.57541	4.218908	0.57102	7.388368	1.49E-13	1.28E-11	FRMD3	257019	FERM domain containing 3
ENSG0000	119.4693	2.360905	0.32096	7.355759	1.90E-13	1.60E-11	PM20D2	135293	peptidase M20 domain containing 2
ENSG0000	237.2115	6.913377	0.941472	7.343157	2.09E-13	1.74E-11	SLC14A1	6563	solute carrier family 14 member 1 (Kidd blood group)
ENSG0000	637.1536	-4.08734	0.557733	-7.32849	2.33E-13	1.94E-11	IGSF10	285313	immunoglobulin superfamily member 10
ENSG0000	356.587	3.574173	0.48779	7.327279	2.35E-13	1.95E-11	UBASH3B	84959	ubiquitin associated and SH3 domain containing B
ENSG0000	153.282	4.630197	0.633003	7.314657	2.58E-13	2.13E-11	CDK15	65061	cyclin dependent kinase 15
ENSG0000	2548.446	5.372185	0.735852	7.300637	2.86E-13	2.35E-11	ENPP1	5167	ectonucleotide pyrophosphatase/phosphodiesterase 1
ENSG0000	26.50738	3.740349	0.512564	7.297333	2.94E-13	2.40E-11	LTK	4058	leukocyte receptor tyrosine kinase

Supp Table 3 WNT4siRNA vs NS

ENSG	baseMean	log2FoldC	lfcSE	stat	pvalue	padj	symbol	entrez	genename
ENSG0000	42569.34	-7.7639	0.27807	-27.9207	1.50E-171	3.71E-167	FOSB	2354	FosB proto-oncogene, AP-1 transcription factor subunit
ENSG0000	7639.531	-5.65231	0.303536	-18.6215	2.15E-77	2.67E-73	HES1	3280	hes family bHLH transcription factor 1
ENSG0000	27781.43	-2.8726	0.159069	-18.0589	6.72E-73	5.55E-69	ND4L	4539	NADH dehydrogenase subunit 4L
ENSG0000	886.8821	-3.78813	0.212231	-17.8491	2.94E-71	1.82E-67	NA	NA	NA
ENSG0000	200.9244	8.123774	0.458026	17.73647	2.19E-70	1.09E-66	ZFY	7544	zinc finger protein Y-linked
ENSG0000	241026.6	-2.76214	0.158198	-17.4601	2.88E-68	1.19E-64	ND4	4538	NADH dehydrogenase subunit 4
ENSG0000	45191.96	-2.80377	0.160701	-17.4471	3.62E-68	1.28E-64	MTATP6P	106480796	MT-ATP6 pseudogene 1
ENSG0000	1135.605	14.58525	0.87707	16.62951	4.26E-62	1.32E-58	RPS4Y1	6192	ribosomal protein S4 Y-linked 1
ENSG0000	113438.7	-2.62919	0.159284	-16.5063	3.30E-61	9.10E-58	ATP6	4508	ATP synthase F0 subunit 6
ENSG0000	35198.71	-4.34588	0.27335	-15.8986	6.48E-57	1.61E-53	ZFP36	7538	ZFP36 ring finger protein
ENSG0000	737.7384	1.880378	0.118758	15.83374	1.82E-56	4.10E-53	UTP25	27042	UTP25 small subunit processome component
ENSG0000	145474.6	-2.72256	0.1734	-15.7011	1.49E-55	3.07E-52	COX3	4514	cytochrome c oxidase subunit III
ENSG0000	16798.62	-2.69868	0.175677	-15.3616	2.96E-53	5.65E-50	MTCO3P1	107075270	MT-CO3 pseudogene 12
ENSG0000	548.3524	10.86476	0.711767	15.26449	1.32E-52	2.33E-49	DDX3Y	8653	DEAD-box helicase 3 Y-linked
ENSG0000	9590.732	-2.91322	0.198917	-14.6454	1.44E-48	2.38E-45	JUND	3727	JunD proto-oncogene, AP-1 transcription factor subunit
ENSG0000	111441.3	-2.89689	0.199778	-14.5005	1.20E-47	1.86E-44	ND2	4536	NADH dehydrogenase subunit 2
ENSG0000	234.8087	12.31139	0.884029	13.92645	4.38E-44	6.38E-41	KDM5D	8284	lysine demethylase 5D
ENSG0000	2573.298	-1.57177	0.114289	-13.7526	4.91E-43	6.77E-40	UBL5	59286	ubiquitin like 5
ENSG0000	1989.133	-1.59746	0.119947	-13.318	1.82E-40	2.37E-37	UQCRQ	27089	ubiquinol-cytochrome c reductase complex III subunit VII
ENSG0000	1621.231	1.599975	0.121543	13.16383	1.42E-39	1.76E-36	OTUD4	54726	OTU deubiquitinase 4
ENSG0000	158.9077	11.7479	0.902959	13.01044	1.07E-38	1.26E-35	NA	NA	NA
ENSG0000	3060.633	-3.91124	0.301263	-12.9828	1.53E-38	1.73E-35	NA	NA	NA
ENSG0000	13948.45	-2.58024	0.199973	-12.9029	4.33E-38	4.67E-35	NA	NA	NA
ENSG0000	4952.073	-2.34633	0.182492	-12.8572	7.84E-38	8.10E-35	MTCO2P1	107075310	MT-CO2 pseudogene 12
ENSG0000	3163.315	-1.59058	0.125387	-12.6854	7.13E-37	7.07E-34	COX6B1	1340	cytochrome c oxidase subunit 6B1
ENSG0000	692.2106	1.680571	0.134411	12.5032	7.17E-36	6.84E-33	RO60	6738	Ro60, Y RNA binding protein
ENSG0000	1495.343	1.509476	0.123384	12.23398	2.05E-34	1.88E-31	ACAP2	23527	ArfGAP with coiled-coil, ankyrin repeat and PH domains 2
ENSG0000	142693.6	-2.92424	0.241552	-12.106	9.81E-34	8.69E-31	CYTB	4519	cytochrome b
ENSG0000	723.2157	1.539076	0.129954	11.84328	2.33E-32	1.99E-29	NAA15	80155	N-alpha-acetyltransferase 15, NatA auxiliary subunit
ENSG0000	531.1468	1.711242	0.144793	11.81855	3.13E-32	2.51E-29	NAA30	122830	N-alpha-acetyltransferase 30, NatC catalytic subunit
ENSG0000	5713.321	-4.60086	0.389304	-11.8182	3.14E-32	2.51E-29	SNAI1	6615	snail family transcriptional repressor 1
ENSG0000	1406.287	-1.80574	0.15353	-11.7615	6.16E-32	4.63E-29	BCL3	602	BCL3 transcription coactivator
ENSG0000	127.7078	3.202471	0.272722	11.74261	7.71E-32	5.62E-29	NPTX1	4884	neuronal pentraxin 1
ENSG0000	1117.224	-9.59184	0.818986	-11.7118	1.11E-31	7.85E-29	HOXC10	3226	homeobox C10
ENSG0000	943.8171	2.781861	0.240943	11.54572	7.76E-31	5.34E-28	ZNF281	23528	zinc finger protein 281
ENSG0000	4731.955	-3.42369	0.297794	-11.4968	1.37E-30	9.17E-28	PIM1	5292	Pim-1 proto-oncogene, serine/threonine kinase
ENSG0000	10140.7	-2.49451	0.218086	-11.4382	2.69E-30	1.76E-27	KLF10	7071	KLF transcription factor 10
ENSG0000	1460.826	-3.03682	0.265748	-11.4275	3.05E-30	1.89E-27	NA	NA	NA
ENSG0000	1589.296	-2.32168	0.203263	-11.4221	3.24E-30	1.96E-27	NA	NA	NA
ENSG0000	136420	-2.45231	0.214811	-11.4161	3.47E-30	2.05E-27	COX2	4513	cytochrome c oxidase subunit II
ENSG0000	2216.288	2.00458	0.176327	11.36851	6.00E-30	3.46E-27	CEP170	9859	centrosomal protein 170
ENSG0000	510187.8	-2.6722	0.23553	-11.3455	7.81E-30	4.40E-27	COX1	4512	cytochrome c oxidase subunit I
ENSG0000	103.0381	10.1574	0.899598	11.29105	1.45E-29	8.00E-27	EIF1AY	9086	eukaryotic translation initiation factor 1A Y-linked
ENSG0000	5508.262	-3.21768	0.285826	-11.2575	2.13E-29	1.15E-26	CSRNP1	64651	cysteine and serine rich nuclear protein 1
ENSG0000	8849.998	-2.48533	0.22101	-11.2453	2.44E-29	1.29E-26	MIDN	90007	midnolin
ENSG0000	1072.409	1.809921	0.161426	11.21207	3.56E-29	1.84E-26	KAT6A	7994	lysine acetyltransferase 6A
ENSG0000	101.7878	10.6225	0.949877	11.18303	4.94E-29	2.50E-26	UTY	7404	ubiquitously transcribed tetratricopeptide repeat containing, Y-linked
ENSG0000	899.0424	1.658674	0.14841	11.17633	5.32E-29	2.64E-26	QSER1	79832	glutamine and serine rich 1
ENSG0000	51957.04	-3.19354	0.286589	-11.1433	7.72E-29	3.68E-26	MTND2P2	100652939	MT-ND2 pseudogene 28
ENSG0000	1080.429	1.774589	0.160908	11.02863	2.78E-28	1.28E-25	MKLN1	4289	muskelin 1
ENSG0000	865.9303	2.049372	0.186697	10.97698	4.93E-28	2.21E-25	ASXL2	55252	ASXL transcriptional regulator 2
ENSG0000	5173.153	-4.17654	0.380515	-10.976	4.98E-28	2.21E-25	PER1	5187	period circadian regulator 1
ENSG0000	151.1404	8.342625	0.763524	10.92648	8.61E-28	3.75E-25	PRKY	5616	protein kinase Y-linked (pseudogene)
ENSG0000	1536.854	1.645435	0.151745	10.84341	2.14E-27	9.01E-25	PRKD3	23683	protein kinase D3
ENSG0000	4526.074	-3.96342	0.366506	-10.8141	2.95E-27	1.22E-24	OSR2	116039	odd-skipped related transciption factor 2
ENSG0000	23080.74	-2.62282	0.242807	-10.8021	3.37E-27	1.37E-24	JUNB	3726	JunB proto-oncogene, AP-1 transcription factor subunit
ENSG0000	346.8047	1.655766	0.153819	10.76438	5.07E-27	2.03E-24	THAP5	168451	THAP domain containing 5
ENSG0000	6592.042	-3.02651	0.281536	-10.75	5.93E-27	2.33E-24	SOCS3	9021	suppressor of cytokine signaling 3
ENSG0000	169780.4	-2.57238	0.240825	-10.6815	1.24E-26	4.74E-24	MTCO1P1	107075141	MT-CO1 pseudogene 12
ENSG0000	9394.227	-2.15364	0.202945	-10.612	2.62E-26	9.56E-24	ATP8	4509	ATP synthase F0 subunit 8
ENSG0000	755.3817	1.653034	0.156204	10.58253	3.59E-26	1.29E-23	TBL1X	6907	transducin beta like 1 X-linked
ENSG0000	4352.045	-1.80782	0.172375	-10.4877	9.84E-26	3.48E-23	POLR2L	5441	RNA polymerase II, I and III subunit L
ENSG0000	2026.408	-1.74173	0.166719	-10.4471	1.51E-25	5.27E-23	NEU1	4758	neuraminidase 1
ENSG0000	275.5861	2.39791	0.230006	10.42542	1.90E-25	6.45E-23	NA	NA	NA
ENSG0000	115136.2	-2.52051	0.242693	-10.3856	2.88E-25	9.66E-23	ND5	4540	NADH dehydrogenase subunit 5
ENSG0000	1006.634	1.563197	0.151115	10.34445	4.43E-25	1.45E-22	STK24	8428	serine/threonine kinase 24
ENSG0000	900.8513	1.547764	0.149706	10.33871	4.71E-25	1.52E-22	FBXO11	80204	F-box protein 11
ENSG0000	12458.39	-1.86508	0.181471	-10.2775	8.90E-25	2.79E-22	SERF2	10169	small EDRK-rich factor 2
ENSG0000	3070.963	2.689862	0.26266	10.24084	1.30E-24	4.03E-22	WARS1	7453	tryptophanyl-tRNA synthetase 1
ENSG0000	1343.912	1.760651	0.172601	10.20068	1.97E-24	6.03E-22	LRRC58	116064	leucine rich repeat containing 58
ENSG0000	748.4363	-1.57886	0.155636	-10.1445	3.50E-24	1.05E-21	CCS	9973	copper chaperone for superoxide dismutase
ENSG0000	686.1102	-4.10055	0.404759	-10.1308	4.03E-24	1.19E-21	CLIC3	9022	chloride intracellular channel 3
ENSG0000	93470.96	-2.65252	0.261949	-10.1261	4.23E-24	1.23E-21	ND1	4535	NADH dehydrogenase subunit 1
ENSG0000	920.7717	-1.66221	0.164653	-10.0952	5.80E-24	1.65E-21	ME3	10873	malic enzyme 3
ENSG0000	662.8334	1.774347	0.175865	10.08924	6.16E-24	1.74E-21	EHD4	30844	EH domain containing 4
ENSG0000	1868.256	2.426443	0.240704	10.08062	6.73E-24	1.87E-21	SYNJ2	8871	synaptojanin 2
ENSG0000	616.927	1.930152	0.192757	10.0134	1.33E-23	3.63E-21	ASAP2	8853	ArfGAP with SH3 domain, ankyrin repeat and PH domain 2
ENSG0000	84.21003	3.024311	0.302063	10.01219	1.35E-23	3.63E-21	GPM6B	2824	glycoprotein M6B
ENSG0000	1092.269	-1.56029	0.156121	-9.99407	1.62E-23	4.31E-21	NDUFB1	4707	NADH:ubiquinone oxidoreductase subunit B1
ENSG0000	270.4014	-2.56237	0.257087	-9.96693	2.13E-23	5.61E-21	CES4A	283848	carboxylesterase 4A
ENSG0000	886.4777	-1.72693	0.1736	-9.94775	2.58E-23	6.73E-21	TNFSF12	8742	TNF superfamily member 12
ENSG0000	2118.129	-1.64121	0.166908	-9.83302	8.12E-23	2.07E-20	PHPT1	29085	phosphohistidine phosphatase 1
ENSG0000	1728.303	-1.52014	0.154938	-9.81123	1.01E-22	2.54E-20	JMJD6	23210	jumonji domain containing 6, arginine demethylase and lysine hydroxylase
ENSG0000	210.2386	-2.3317	0.237675	-9.81045	1.02E-22	2.54E-20	TMEM256	254863	transmembrane protein 256
ENSG0000	15255.63	-4.57979	0.467232	-9.80195	1.10E-22	2.74E-20	NR4A1	3164	nuclear receptor subfamily 4 group A member 1
ENSG0000	16665.79	-4.27657	0.438067	-9.76237	1.63E-22	3.97E-20	BHLHE40	8553	basic helix-loop-helix family member e40

ENSG0000	744.8151	2.646944	0.272639	9.70862	2.77E-22	6.67E-20	NA	NA	NA
ENSG0000	2527.148	1.785204	0.184255	9.688744	3.37E-22	8.02E-20	TAOK1	57551	TAO kinase 1
ENSG0000	354.8679	1.580226	0.16313	9.686902	3.43E-22	8.09E-20	TGS1	96764	trimethylguanosine synthase 1
ENSG0000	882.7569	-1.76649	0.184842	-9.5568	1.21E-21	2.71E-19	NDUFA3	4696	NADH:ubiquinone oxidoreductase subunit A3
ENSG0000	790.9217	1.501481	0.158127	9.495413	2.19E-21	4.81E-19	JMY	133746	junction mediating and regulatory protein, p53 cofactor
ENSG0000	261.3183	-4.76578	0.504644	-9.44385	3.59E-21	7.75E-19	IDI2-AS1	55853	IDI2 antisense RNA 1
ENSG0000	388.1229	1.571462	0.167222	9.397445	5.59E-21	1.18E-18	APAF1	317	apoptotic peptidase activating factor 1
ENSG0000	57.64641	6.469493	0.693019	9.335235	1.01E-20	2.12E-18	GALNT14	79623	polypeptide N-acetylgalactosaminyltransferase 14
ENSG0000	1640.883	2.058214	0.220978	9.314096	1.23E-20	2.54E-18	SH3KBP1	30011	SH3 domain containing kinase binding protein 1
ENSG0000	24.25195	9.035691	0.970809	9.307382	1.31E-20	2.68E-18	TTTY14	83869	testis-specific transcript, Y-linked 14
ENSG0000	1165.108	-1.82892	0.197025	-9.28268	1.65E-20	3.36E-18	IFI27L2	83982	interferon alpha inducible protein 27 like 2
ENSG0000	12511.59	-2.36102	0.2551	-9.25527	2.14E-20	4.31E-18	MTND1P2	100887749	MT-ND1 pseudogene 23
ENSG0000	6562.526	1.668028	0.181005	9.215388	3.10E-20	6.10E-18	AXL	558	AXL receptor tyrosine kinase
ENSG0000	281.5735	2.261721	0.245575	9.20989	3.26E-20	6.37E-18	CDYL2	124359	chromodomain Y like 2
ENSG0000	553.2377	-1.54052	0.167334	-9.20628	3.38E-20	6.54E-18	NDUFA11	126328	NADH:ubiquinone oxidoreductase subunit A11
ENSG0000	387.6861	1.95829	0.213207	9.184918	4.12E-20	7.91E-18	NBEAL1	65065	neurobeachin like 1
ENSG0000	95.84279	2.505626	0.272843	9.183394	4.18E-20	7.97E-18	PM20D2	135293	peptidase M20 domain containing 2
ENSG0000	584.9485	-1.80013	0.196609	-9.15587	5.39E-20	1.02E-17	LINC01503	100506119	long intergenic non-protein coding RNA 1503
ENSG0000	1802.796	-4.47134	0.490978	-9.10701	8.47E-20	1.58E-17	ATF3	467	activating transcription factor 3
ENSG0000	3162.75	1.761101	0.193419	9.105125	8.62E-20	1.59E-17	ARHGAP35	2909	Rho GTPase activating protein 35
ENSG0000	1238.011	-1.51281	0.166239	-9.10019	9.02E-20	1.66E-17	LAMTOR5	10542	late endosomal/lysosomal adaptor, MAPK and MTOR activat or 5
ENSG0000	1193.78	1.797239	0.197563	9.097053	9.28E-20	1.69E-17	SPTLC2	9517	serine palmitoyltransferase long chain base subunit 2
ENSG0000	952.5745	-6.17252	0.678854	-9.09256	9.67E-20	1.75E-17	HEY1	23462	hes related family bHLH transcription factor with YRPW motif 1
ENSG0000	288.1785	1.782216	0.196273	9.08027	1.08E-19	1.93E-17	NDC1	55706	NDC1 transmembrane nucleoporin
ENSG0000	140.3093	2.427165	0.267952	9.058221	1.33E-19	2.35E-17	E2F7	144455	E2F transcription factor 7
ENSG0000	152.7582	-1.96478	0.217284	-9.04246	1.53E-19	2.69E-17	ZSCAN16-	100129195	ZSCAN16 antisense RNA 1
ENSG0000	477.8323	-5.12071	0.56799	-9.01549	1.96E-19	3.42E-17	TBX1	6899	T-box transcription factor 1
ENSG0000	595.4248	-1.65043	0.183327	-9.00262	2.20E-19	3.79E-17	DYNLRB1	83658	dynein light chain roadblock-type 1
ENSG0000	551.1471	-2.70268	0.300479	-8.99457	2.37E-19	4.05E-17	LOC10050	100507053	uncharacterized LOC100507053
ENSG0000	317.9368	1.817747	0.202871	8.960117	3.24E-19	5.36E-17	PLAGL2	5326	PLAG1 like zinc finger 2
ENSG0000	479.1136	-1.76568	0.197193	-8.95406	3.43E-19	5.63E-17	BLOC1S1	2647	biogenesis of lysosomal organelles complex 1 subunit 1
ENSG0000	795.4548	1.826571	0.204683	8.923922	4.50E-19	7.29E-17	GTF3C4	9329	general transcription factor IIIC subunit 4
ENSG0000	1018.318	1.877267	0.210594	8.914151	4.92E-19	7.86E-17	XIAP	331	X-linked inhibitor of apoptosis
ENSG0000	2822.808	1.899479	0.213479	8.897742	5.70E-19	9.06E-17	ZBTB38	253461	zinc finger and BTB domain containing 38
ENSG0000	289.9603	-5.1993	0.584525	-8.89491	5.85E-19	9.23E-17	HOXB7	3217	homeobox B7
ENSG0000	133.1694	-4.91344	0.557896	-8.80708	1.28E-18	2.00E-16	NA	NA	NA
ENSG0000	66.23613	5.978278	0.678884	8.806032	1.30E-18	2.01E-16	HOXD10	3236	homeobox D10
ENSG0000	118.3514	-5.00746	0.568804	-8.80349	1.33E-18	2.04E-16	NA	NA	NA
ENSG0000	33.60359	3.509511	0.400294	8.767334	1.83E-18	2.77E-16	CDCA7	83879	cell division cycle associated 7
ENSG0000	11557.3	-3.25623	0.371673	-8.76101	1.94E-18	2.91E-16	ID3	3399	inhibitor of DNA binding 3
ENSG0000	1514.83	-2.98554	0.342689	-8.7121	2.98E-18	4.35E-16	MAP3K8	1326	mitogen-activated protein kinase kinase kinase 8
ENSG0000	6182.552	2.554244	0.293427	8.70487	3.18E-18	4.61E-16	CD109	135228	CD109 molecule
ENSG0000	27442.15	-1.56767	0.180145	-8.70227	3.25E-18	4.69E-16	CDKN1A	1026	cyclin dependent kinase inhibitor 1A
ENSG0000	826.0215	-4.77212	0.548902	-8.69393	3.50E-18	4.99E-16	ABCA10	10349	ATP binding cassette subfamily A member 10
ENSG0000	119.2825	-4.63107	0.534989	-8.65638	4.87E-18	6.82E-16	NA	NA	NA
ENSG0000	837.9095	-1.63261	0.18869	-8.65231	5.05E-18	7.03E-16	ATP5ME	521	ATP synthase membrane subunit e
ENSG0000	2365.155	-1.54089	0.178216	-8.64621	5.32E-18	7.37E-16	YPEL3	83719	yippee like 3
ENSG0000	1383.264	2.425339	0.281338	8.620738	6.65E-18	9.06E-16	ABHD2	11057	abhydrolase domain containing 2, acylglycerol lipase
ENSG0000	245.4669	-3.70913	0.431696	-8.592	8.55E-18	1.13E-15	CACNB2	783	calcium voltage-gated channel auxiliary subunit beta 2
ENSG0000	191.9649	1.925377	0.224317	8.583279	9.22E-18	1.20E-15	AGPAT5	55326	1-acylglycerol-3-phosphate O-acyltransferase 5
ENSG0000	823.6506	1.890029	0.221016	8.551531	1.21E-17	1.57E-15	SOCS6	9306	suppressor of cytokine signaling 6
ENSG0000	1761.531	-3.05056	0.357231	-8.53946	1.35E-17	1.73E-15	RNA5-8SN	109910381	RNA, 5.8S ribosomal N3
ENSG0000	3401.561	1.99767	0.234141	8.531896	1.44E-17	1.83E-15	SLFN5	162394	schlafen family member 5
ENSG0000	1760.062	-3.04931	0.357682	-8.5252	1.53E-17	1.92E-15	RNA5-8SN	106632260	RNA, 5.8S ribosomal N1
ENSG0000	1760.618	-3.04978	0.357758	-8.52472	1.53E-17	1.92E-15	RNA5-8SN	109864281	RNA, 5.8S ribosomal N2
ENSG0000	120.0115	-4.88681	0.573261	-8.52458	1.53E-17	1.92E-15	NA	NA	NA
ENSG0000	1759.15	-3.04853	0.358208	-8.51049	1.73E-17	2.12E-15	LOC12490	124900510	5.8S ribosomal RNA
ENSG0000	1759.15	-3.04853	0.358208	-8.51049	1.73E-17	2.12E-15	LOC12490	124900510	5.8S ribosomal RNA
ENSG0000	1759.15	-3.04853	0.358208	-8.51049	1.73E-17	2.12E-15	RNA5-8SP	109910384	RNA, 5.8S ribosomal pseudogene 10
ENSG0000	1758.746	-3.04819	0.358383	-8.50539	1.81E-17	2.19E-15	RNA5-8SN	100008587	RNA, 5.8S ribosomal N5
ENSG0000	1758.746	-3.04819	0.358383	-8.50539	1.81E-17	2.19E-15	RNA5-8SN	109864274	RNA, 5.8S ribosomal N4
ENSG0000	1728.475	1.949412	0.229326	8.5006	1.89E-17	2.27E-15	NFAT5	10725	nuclear factor of activated T cells 5
ENSG0000	783.8837	1.515704	0.178575	8.487765	2.11E-17	2.52E-15	ATP11C	286410	ATPase phospholipid transporting 11C
ENSG0000	785.663	1.659061	0.195679	8.478483	2.28E-17	2.72E-15	ETV5	2119	ETS variant transcription factor 5
ENSG0000	254.6769	-7.87073	0.930642	-8.45731	2.74E-17	3.21E-15	LEP	3952	leptin
ENSG0000	434.7011	-2.70376	0.319768	-8.45537	2.78E-17	3.25E-15	HSD17B14	51171	hydroxysteroid 17-beta dehydrogenase 14
ENSG0000	232.5945	1.599664	0.189375	8.447085	2.99E-17	3.48E-15	SEC63P1	100420341	SEC63 homolog, protein translocation regulator pseudogene 1
ENSG0000	791.7443	1.972778	0.234223	8.422632	3.68E-17	4.23E-15	NEDD1	121441	NEDD1 gamma-tubulin ring complex targeting factor
ENSG0000	1736.837	-12.2186	1.451321	-8.41893	3.80E-17	4.32E-15	XIST	7503	X inactive specific transcript
ENSG0000	1370.121	1.926448	0.229001	8.412408	4.02E-17	4.55E-15	ITPRIPL2	162073	ITPRIP like 2
ENSG0000	876.5494	-3.54463	0.422034	-8.39894	4.51E-17	5.03E-15	RASD1	51655	ras related dexamethasone induced 1
ENSG0000	301.7987	1.764982	0.211184	8.357555	6.40E-17	6.96E-15	MRTFB	57496	myocardin related transcription factor B
ENSG0000	1214.238	-2.25174	0.269495	-8.3554	6.52E-17	7.06E-15	TMSB4XP4	7118	TMSB4X pseudogene 4
ENSG0000	581.2861	1.724352	0.207886	8.29472	1.09E-16	1.16E-14	NRIP1	8204	nuclear receptor interacting protein 1
ENSG0000	479.371	-1.81317	0.21871	-8.29026	1.13E-16	1.20E-14	MATCAP1	653319	microtubule associated tyrosine carboxypeptidase 1
ENSG0000	1475.804	2.027863	0.244759	8.285134	1.18E-16	1.24E-14	HSPA13	6782	heat shock protein family A (Hsp70) member 13
ENSG0000	538.763	2.145508	0.259002	8.283746	1.19E-16	1.25E-14	NA	NA	NA
ENSG0000	92.67046	-2.62851	0.317605	-8.27603	1.27E-16	1.33E-14	RPL36AP4	441727	ribosomal protein L36a pseudogene 45
ENSG0000	94.18352	-5.6889	0.687503	-8.27472	1.29E-16	1.34E-14	HOXC-AS1	100874363	HOXC cluster antisense RNA 1
ENSG0000	11445.74	-3.82633	0.46251	-8.27298	1.31E-16	1.35E-14	ID1	3397	inhibitor of DNA binding 1
ENSG0000	2402.182	-4.00104	0.484156	-8.26394	1.41E-16	1.44E-14	TRIB1	10221	tribbles pseudokinase 1
ENSG0000	29690.26	-2.21302	0.268492	-8.24242	1.69E-16	1.72E-14	ND6	4541	NADH dehydrogenase subunit 6
ENSG0000	843.6818	-2.79561	0.339189	-8.24204	1.69E-16	1.72E-14	NA	NA	NA
ENSG0000	213.3514	2.43977	0.296328	8.233333	1.82E-16	1.84E-14	LRRC8C	84230	leucine rich repeat containing 8 VRAC subunit C
ENSG0000	140.9099	2.392717	0.290713	8.230516	1.86E-16	1.88E-14	BRCA2	675	BRCA2 DNA repair associated
ENSG0000	859.7062	-2.10841	0.256309	-8.22603	1.94E-16	1.93E-14	NR2F1	7025	nuclear receptor subfamily 2 group F member 1
ENSG0000	552.1955	1.675027	0.203946	8.213095	2.16E-16	2.15E-14	AFAP1	60312	actin filament associated protein 1

ENSG0000	124.9972	-3.34374	0.407745	-8.20057	2.39E-16	2.35E-14	LINC02615	100507487	long intergenic non-protein coding RNA 2615
ENSG0000	421.3938	3.333808	0.407002	8.191126	2.59E-16	2.53E-14	INSYN2B	100131897	inhibitory synaptic factor family member 2B
ENSG0000	381.777	-2.35735	0.287854	-8.1894	2.63E-16	2.54E-14	NA	NA	NA
ENSG0000	2536.062	1.532345	0.18739	8.177315	2.90E-16	2.79E-14	GOLGA4	2803	golgin A4
ENSG0000	497.931	3.091634	0.378095	8.176873	2.91E-16	2.79E-14	ZNF770	54989	zinc finger protein 770
ENSG0000	828.6896	2.52389	0.308733	8.174982	2.96E-16	2.82E-14	NA	NA	NA
ENSG0000	1617.83	1.525879	0.18791	8.12025	4.65E-16	4.34E-14	CNOT1	23019	CCR4-NOT transcription complex subunit 1
ENSG0000	1525.242	1.644605	0.202942	8.103828	5.33E-16	4.89E-14	AKAP11	11215	A-kinase anchoring protein 11
ENSG0000	99197.88	-1.82957	0.226103	-8.09173	5.88E-16	5.36E-14	NA	NA	NA
ENSG0000	532.0844	1.591591	0.197	8.079134	6.52E-16	5.88E-14	RPS27AP1	643358	RPS27A pseudogene 16
ENSG0000	89.34327	1.888525	0.234423	8.056071	7.88E-16	7.03E-14	HNRNPKP	644063	heterogeneous nuclear ribonucleoprotein K pseudogene 4
ENSG0000	560.0926	1.956532	0.243745	8.026951	9.99E-16	8.82E-14	PI4K2B	55300	phosphatidylinositol 4-kinase type 2 beta
ENSG0000	187.0027	-1.92135	0.239451	-8.02395	1.02E-15	8.94E-14	NA	NA	NA
ENSG0000	2001.595	-1.85116	0.230705	-8.02392	1.02E-15	8.94E-14	NA	NA	NA
ENSG0000	104.4869	1.531246	0.190857	8.023003	1.03E-15	8.98E-14	SUV39H2	79723	SUV39H2 histone lysine methyltransferase
ENSG0000	75.55588	2.495096	0.31282	7.976136	1.51E-15	1.30E-13	NA	NA	NA
ENSG0000	253.2461	-2.16813	0.273112	-7.9386	2.04E-15	1.72E-13	NA	NA	NA
ENSG0000	866.6064	1.778529	0.224117	7.935728	2.09E-15	1.75E-13	ZFHX3	463	zinc finger homeobox 3
ENSG0000	555.7485	-2.35685	0.297219	-7.92966	2.20E-15	1.82E-13	SLC4A3	6508	solute carrier family 4 member 3
ENSG0000	755.0404	1.601232	0.201945	7.929066	2.21E-15	1.82E-13	DENND4C	55667	DENN domain containing 4C
ENSG0000	643.3359	-1.821	0.229702	-7.92767	2.23E-15	1.84E-13	PSENEN	55851	presenilin enhancer, gamma-secretase subunit
ENSG0000	2501.932	-1.64545	0.207642	-7.92446	2.29E-15	1.88E-13	NRBP2	340371	nuclear receptor binding protein 2
ENSG0000	143.4934	-5.46472	0.690601	-7.91299	2.51E-15	2.05E-13	HOXB5	3215	homeobox B5
ENSG0000	186.7282	-1.58467	0.200473	-7.90462	2.69E-15	2.18E-13	AAMDC	28971	adipogenesis associated Mth938 domain containing
ENSG0000	675.9294	1.76748	0.223678	7.90188	2.75E-15	2.22E-13	TRAF3	7187	TNF receptor associated factor 3
ENSG0000	14030.62	-2.77823	0.352791	-7.87499	3.41E-15	2.73E-13	DUSP1	1843	dual specificity phosphatase 1
ENSG0000	630.0246	1.903581	0.242192	7.859814	3.85E-15	3.07E-13	PPARA	5465	peroxisome proliferator activated receptor alpha

Supp Table 4 WNT4siRNA vs DDC comparison

ENSG	baseMean	log2FoldC	lfcSE	stat	pvalue	padj	symbol	entrez	genename
ENSG0000	588.6115	2.919799	0.275605	10.59415	3.17E-26	6.11E-22	JAM2	58494	junctional adhesion molecule 2
ENSG0000	544.9799	-3.16295	0.394728	-8.01299	1.12E-15	1.08E-11	NA	NA	NA
ENSG0000	335.1462	-4.22091	0.593949	-7.10653	1.19E-12	7.65E-09	DIRAS3	9077	DIRAS family GTPase 3
ENSG0000	37121.62	2.761846	0.419628	6.58166	4.65E-11	2.24E-07	COL8A1	1295	collagen type VIII alpha 1 chain
ENSG0000	1867.677	3.189123	0.49651	6.423083	1.34E-10	5.15E-07	SERPINB7	8710	serpin family B member 7
ENSG0000	60.90818	5.935052	0.945701	6.275826	3.48E-10	1.12E-06	PRR15	222171	proline rich 15
ENSG0000	1338.936	-2.131	0.343118	-6.2107	5.27E-10	1.45E-06	NA	NA	NA
ENSG0000	53.25197	4.780003	0.787529	6.069621	1.28E-09	3.09E-06	GALNT13	114805	polypeptide N-acetylgalactosaminyltransferase 13
ENSG0000	392.7971	6.63731	1.162268	5.710654	1.13E-08	2.41E-05	CMPK2	129607	cytidine/uridine monophosphate kinase 2
ENSG0000	4106.952	6.678376	1.215098	5.496162	3.88E-08	7.48E-05	IFIT2	3433	interferon induced protein with tetratricopeptide repeats 2
ENSG0000	627.161	-2.05412	0.376547	-5.45515	4.89E-08	8.57E-05	NA	NA	NA
ENSG0000	428.8363	5.279972	0.973038	5.426276	5.75E-08	9.24E-05	PRLR	5618	prolactin receptor
ENSG0000	3448.299	3.300067	0.613122	5.382395	7.35E-08	0.000109	COL11A1	1301	collagen type XI alpha 1 chain
ENSG0000	336.3491	-2.24051	0.433854	-5.1642	2.41E-07	0.000332	NA	NA	NA
ENSG0000	387.8715	6.896905	1.36555	5.050642	4.40E-07	0.000501	RSAD2	91543	radical S-adenosyl methionine domain containing 2
ENSG0000	120.1702	-3.74475	0.742655	-5.04238	4.60E-07	0.000501	TRABD2A	129293	TraB domain containing 2A
ENSG0000	96.44115	-2.76047	0.551535	-5.00506	5.58E-07	0.000567	SOX8	30812	SRY-box transcription factor 8
ENSG0000	3095.927	6.207052	1.254257	4.948789	7.47E-07	0.00072	MX1	4599	MX dynamin like GTPase 1
ENSG0000	49.63955	-5.16511	1.046269	-4.93669	7.95E-07	0.000729	ASS1P11	340274	argininosuccinate synthetase 1 pseudogene 11
ENSG0000	1490.055	6.597764	1.348187	4.893805	9.89E-07	0.000815	IFI44L	10964	interferon induced protein 44 like
ENSG0000	157.0153	4.025698	0.824446	4.882916	1.05E-06	0.000815	ISG20	3669	interferon stimulated exonuclease gene 20
ENSG0000	310.6004	-2.08021	0.42612	-4.88174	1.05E-06	0.000815	NA	NA	NA
ENSG0000	43.1963	2.920035	0.598979	4.875024	1.09E-06	0.000815	KCTD16	57528	potassium channel tetramerization domain containing 16
ENSG0000	1142.133	2.41888	0.496382	4.873025	1.10E-06	0.000815	FBXO32	114907	F-box protein 32
ENSG0000	82.07448	3.72257	0.772718	4.817503	1.45E-06	0.001038	ARFGEF3	57221	ARFGEF family member 3
ENSG0000	592.2542	-4.5623	0.951406	-4.79532	1.62E-06	0.001088	CLEC3B	7123	C-type lectin domain family 3 member B
ENSG0000	25.47056	2.788501	0.581693	4.793767	1.64E-06	0.001088	NA	NA	NA
ENSG0000	35.84607	4.056175	0.863736	4.696084	2.65E-06	0.001649	KLHDC7B	113730	kelch domain containing 7B
ENSG0000	1129.826	-1.7803	0.381103	-4.67145	2.99E-06	0.001758	PLEKHG4	25894	pleckstrin homology and RhoGEF domain containing G4
ENSG0000	164.8238	-2.25931	0.483777	-4.67016	3.01E-06	0.001758	NA	NA	NA
ENSG0000	7396.525	1.765559	0.379418	4.653335	3.27E-06	0.001809	SEMA3C	10512	semaphorin 3C
ENSG0000	116.7964	-1.59783	0.343463	-4.65212	3.29E-06	0.001809	EEF1DP1	126037	eukaryotic translation elongation factor 1 delta pseudogene 1
ENSG0000	2292.891	4.317264	0.937492	4.605122	4.12E-06	0.002191	RIGI	23586	RNA sensor RIG-I
ENSG0000	2254.642	-1.92803	0.419054	-4.60092	4.21E-06	0.002191	NA	NA	NA
ENSG0000	22.51705	2.640494	0.576118	4.583252	4.58E-06	0.00226	NA	NA	NA
ENSG0000	179.027	-5.36627	1.170885	-4.58309	4.58E-06	0.00226	NA	NA	NA
ENSG0000	673.9449	1.694551	0.370133	4.578216	4.69E-06	0.00226	CHAC1	79094	ChaC glutathione specific gamma-glutamylcyclotransferase 1
ENSG0000	1064.773	-1.92326	0.424233	-4.5335	5.80E-06	0.002634	NA	NA	NA
ENSG0000	138.5584	-2.67828	0.591124	-4.53083	5.88E-06	0.002634	RAMP1	10267	receptor activity modifying protein 1
ENSG0000	203.1265	1.608915	0.355811	4.521831	6.13E-06	0.002686	NA	NA	NA
ENSG0000	1604.534	-2.80678	0.625302	-4.48869	7.17E-06	0.002882	MATN2	4147	matrilin 2
ENSG0000	97.0892	4.805622	1.070793	4.487908	7.19E-06	0.002882	RPS17P5	442216	ribosomal protein S17 pseudogene 5
ENSG0000	90.19179	2.915865	0.650285	4.483977	7.33E-06	0.002882	ENPP4	22875	ectonucleotide pyrophosphatase/phosphodiesterase 4
ENSG0000	224.321	-3.02547	0.675299	-4.4802	7.46E-06	0.002882	NA	NA	NA
ENSG0000	173.6768	-2.11689	0.472554	-4.47968	7.48E-06	0.002882	NA	NA	NA
ENSG0000	164.8218	-1.75691	0.392197	-4.47966	7.48E-06	0.002882	MIR381HG	378881	MIR381 host gene
ENSG0000	438.7593	2.030657	0.455442	4.458655	8.25E-06	0.003067	ADGRL4	64123	adhesion G protein-coupled receptor L4
ENSG0000	844.3987	-1.94389	0.436115	-4.45727	8.30E-06	0.003067	RPS26P31	645979	ribosomal protein S26 pseudogene 31
ENSG0000	214.1982	-1.92354	0.431885	-4.45384	8.43E-06	0.003067	NA	NA	NA
ENSG0000	1606.407	5.398277	1.216997	4.435737	9.18E-06	0.003275	SERPINB2	5055	serpin family B member 2
ENSG0000	425.5491	-5.22689	1.180282	-4.42851	9.49E-06	0.003325	SLC14A1	6563	solute carrier family 14 member 1 (Kidd blood group)
ENSG0000	904.8967	-1.91083	0.431987	-4.42336	9.72E-06	0.003343	NA	NA	NA
ENSG0000	2379.451	-1.87185	0.423528	-4.41966	9.89E-06	0.003343	NA	NA	NA
ENSG0000	3918.594	4.940953	1.119837	4.412207	1.02E-05	0.0034	IFIT1	3434	interferon induced protein with tetratricopeptide repeats 1
ENSG0000	489.5228	2.818642	0.642329	4.388158	1.14E-05	0.003686	DUSP4	1846	dual specificity phosphatase 4
ENSG0000	264.7415	4.095594	0.933509	4.387312	1.15E-05	0.003686	LRRN3	54674	leucine rich repeat neuronal 3
ENSG0000	8.505744	6.37649	1.455636	4.380554	1.18E-05	0.003719	SAA1	6288	serum amyloid A1
ENSG0000	65.39616	1.92243	0.439084	4.378277	1.20E-05	0.003719	ZSCAN31	64288	zinc finger and SCAN domain containing 31
ENSG0000	4507.437	4.954956	1.134701	4.366751	1.26E-05	0.003858	IFIT3	3437	interferon induced protein with tetratricopeptide repeats 3
ENSG0000	1362.254	-1.77141	0.407438	-4.34767	1.38E-05	0.004125	PCOLCE2	26577	procollagen C-endopeptidase enhancer 2
ENSG0000	97.22831	-1.60764	0.369976	-4.34526	1.39E-05	0.004125	NA	NA	NA
ENSG0000	562.9538	-3.29323	0.758912	-4.33941	1.43E-05	0.004158	PRUNE2	158471	prune homolog 2 with BCH domain
ENSG0000	61.14294	-3.97846	0.91873	-4.33039	1.49E-05	0.004158	ESPNL	339768	espin like
ENSG0000	2453.547	1.735478	0.401807	4.319185	1.57E-05	0.004298	COL8A2	1296	collagen type VIII alpha 2 chain
ENSG0000	79.58316	-1.95023	0.451778	-4.31678	1.58E-05	0.004298	RPS2P8	440589	ribosomal protein S2 pseudogene 8
ENSG0000	92.55862	3.402565	0.791203	4.300495	1.70E-05	0.004562	KCNJ6	3763	potassium inwardly rectifying channel subfamily J member 6
ENSG0000	915.0482	-1.90254	0.444561	-4.2796	1.87E-05	0.004943	NA	NA	NA
ENSG0000	2817.787	4.848604	1.135033	4.271773	1.94E-05	0.005051	ISG15	9636	ISG15 ubiquitin like modifier
ENSG0000	116.1363	2.565758	0.606374	4.231313	2.32E-05	0.005892	RNF144B	255488	ring finger protein 144B
ENSG0000	264.6389	1.534453	0.363176	4.225089	2.39E-05	0.005979	BCL7A	605	BAF chromatin remodeling complex subunit BCL7A
ENSG0000	1858.938	-2.10094	0.49776	-4.22079	2.43E-05	0.005983	RPSAP61	644464	ribosomal protein SA pseudogene 61
ENSG0000	49.67347	2.353174	0.557736	4.219155	2.45E-05	0.005983	STX11	8676	syntaxin 11
ENSG0000	29.02857	2.843177	0.675737	4.20752	2.58E-05	0.006189	NA	NA	NA
ENSG0000	134.4477	-7.64249	1.817101	-4.20587	2.60E-05	0.006189	LMX1B	4010	LIM homeobox transcription factor 1 beta
ENSG0000	1626.78	-1.82358	0.435915	-4.18335	2.87E-05	0.00667	NA	NA	NA
ENSG0000	28.92304	-5.412	1.294622	-4.18037	2.91E-05	0.006678	NA	NA	NA

ENSG0000	89.78745	-2.88757	0.6912	-4.17762	2.95E-05	0.006679	CEND1	51286	cell cycle exit and neuronal differentiation 1
ENSG0000	2084.587	-2.00358	0.480117	-4.17311	3.00E-05	0.006734	EMILIN2	84034	elastin microfibril interfacer 2
ENSG0000	163.4372	-5.16716	1.242363	-4.15914	3.19E-05	0.007077	PPP2R2B	5521	protein phosphatase 2 regulatory subunit Bbeta
ENSG0000	1187.545	-1.81148	0.436227	-4.1526	3.29E-05	0.0072	WASH5P	375690	WASP family homolog 5, pseudogene
ENSG0000	17.21602	4.214509	1.018348	4.138575	3.49E-05	0.007484	CXCL5	6374	C-X-C motif chemokine ligand 5
ENSG0000	225.3796	3.253603	0.788817	4.124661	3.71E-05	0.007864	PARP8	79668	poly(ADP-ribose) polymerase family member 8
ENSG0000	520.2897	3.814356	0.931728	4.09385	4.24E-05	0.008888	TNFSF4	7292	TNF superfamily member 4
ENSG0000	2589.674	5.167372	1.264417	4.086764	4.37E-05	0.009066	OAS2	4939	2'-5'-oligoadenylate synthetase 2
ENSG0000	353.717	2.250372	0.551911	4.077416	4.55E-05	0.009337	SLC38A4	55089	solute carrier family 38 member 4
ENSG0000	57.97265	-2.20493	0.542931	-4.06116	4.88E-05	0.009907	CTSH	1512	cathepsin H

Supp Table 5 Scrambled siRNA vs DDC comparison

ENSG	baseMean	log2FoldC	lfcSE	stat	pvalue	padj	symbol	entrez	genename
ENSG0000	1168.029	-10.5372	0.610108	-17.271	7.77E-67	1.51E-62	CSF3	1440	colony stimulating factor 3
ENSG0000	1005.208	-5.70716	0.332953	-17.141	7.34E-66	7.11E-62	MMP10	4319	matrix metallopeptidase 10
ENSG0000	1507.56	-10.8871	0.888332	-12.2556	1.57E-34	1.01E-30	CXCL5	6374	C-X-C motif chemokine ligand 5
ENSG0000	415.6995	-6.79103	0.562622	-12.0703	1.52E-33	7.23E-30	CMPK2	129607	cytidine/uridine monophosphate kinase 2
ENSG0000	672.155	-7.34696	0.609542	-12.0533	1.86E-33	7.23E-30	IL33	90865	interleukin 33
ENSG0000	4206.696	-2.6799	0.281417	-9.52287	1.68E-21	5.45E-18	ABL2	27	ABL proto-oncogene 2, non-receptor tyrosine kinase
ENSG0000	51591.71	-10.3191	1.087965	-9.48479	2.43E-21	6.73E-18	CXCL8	3576	C-X-C motif chemokine ligand 8
ENSG0000	14433.35	-8.69368	0.922506	-9.42398	4.34E-21	1.05E-17	SERPINB2	5055	serpin family B member 2
ENSG0000	509.2872	3.830383	0.411197	9.315203	1.22E-20	2.62E-17	NA	NA	NA
ENSG0000	136302.8	-6.59235	0.715008	-9.21996	2.97E-20	5.76E-17	MMP3	4314	matrix metallopeptidase 3
ENSG0000	592.8045	-7.5861	0.841372	-9.01635	1.94E-19	3.43E-16	RSAD2	91543	radical S-adenosyl methionine domain containing 2
ENSG0000	1877.048	-7.57834	0.873074	-8.68006	3.96E-18	6.39E-15	ESM1	11082	endothelial cell specific molecule 1
ENSG0000	1054.869	-1.99607	0.234956	-8.49551	1.97E-17	2.94E-14	CLDN12	9069	claudin 12
ENSG0000	235764	-6.47046	0.781984	-8.27442	1.29E-16	1.79E-13	MMP1	4312	matrix metallopeptidase 1
ENSG0000	521.7681	3.080998	0.378504	8.13993	3.96E-16	5.11E-13	AMOT	154796	angiomotin
ENSG0000	515.7476	6.116866	0.755936	8.091778	5.88E-16	7.13E-13	PRUNE2	158471	prune homolog 2 with BCH domain
ENSG0000	6330.471	1.829488	0.226703	8.069959	7.03E-16	8.02E-13	CREB3L1	90993	cAMP responsive element binding protein 3 like 1
ENSG0000	320.8763	4.440759	0.553981	8.016083	1.09E-15	1.18E-12	NA	NA	NA
ENSG0000	2600.503	-2.99547	0.377222	-7.94089	2.01E-15	1.97E-12	NAMPTP1	646309	nicotinamide phosphoribosyltransferase pseudogene 1
ENSG0000	1448.243	4.808625	0.605661	7.939467	2.03E-15	1.97E-12	MATN2	4147	matrilin 2
ENSG0000	65.33425	-4.17268	0.527294	-7.9134	2.50E-15	2.31E-12	MCTP1	79772	multiple C2 and transmembrane domain containing 1
ENSG0000	1418.688	3.732417	0.478301	7.803492	6.02E-15	5.31E-12	LDB2	9079	LIM domain binding 2
ENSG0000	1004.075	-1.91846	0.248068	-7.73358	1.05E-14	8.82E-12	BTG3	10950	BTG anti-proliferation factor 3
ENSG0000	6589.704	-2.88631	0.373499	-7.72775	1.09E-14	8.85E-12	NAMPT	10135	nicotinamide phosphoribosyltransferase
ENSG0000	100.9507	-4.33354	0.561957	-7.7121	1.24E-14	9.60E-12	DNER	92737	delta/notch like EGF repeat containing
ENSG0000	6450.94	1.95955	0.255957	7.655772	1.92E-14	1.43E-11	TMEM119	338773	transmembrane protein 119
ENSG0000	13246.78	2.004448	0.26498	7.564516	3.89E-14	2.80E-11	THY1	7070	Thy-1 cell surface antigen
ENSG0000	171.2377	4.749533	0.634128	7.489867	6.89E-14	4.78E-11	CPA4	51200	carboxypeptidase A4
ENSG0000	2623.8	-9.06505	1.217801	-7.44378	9.78E-14	6.54E-11	IL24	11009	interleukin 24
ENSG0000	134.3159	5.344963	0.719434	7.429403	1.09E-13	6.72E-11	HSPB3	8988	heat shock protein family B (small) member 3
ENSG0000	123.1106	6.0589	0.815666	7.428163	1.10E-13	6.72E-11	RAMP1	10267	receptor activity modifying protein 1
ENSG0000	703.7069	2.078207	0.279811	7.42718	1.11E-13	6.72E-11	GCNT1	2650	glucosaminyl (N-acetyl) transferase 1
ENSG0000	5408.923	-4.26951	0.579757	-7.36431	1.78E-13	1.05E-10	PTGS2	5743	prostaglandin-endoperoxide synthase 2
ENSG0000	6187.989	1.99282	0.272043	7.325398	2.38E-13	1.36E-10	ADGRA2	25960	adhesion G protein-coupled receptor A2
ENSG0000	586.5834	2.324923	0.320075	7.263688	3.77E-13	2.09E-10	NA	NA	NA
ENSG0000	620.8999	-2.13434	0.296836	-7.19031	6.46E-13	3.48E-10	SLC41A2	84102	solute carrier family 41 member 2
ENSG0000	4180.94	-8.22828	1.146791	-7.17505	7.23E-13	3.79E-10	IL1B	3553	interleukin 1 beta
ENSG0000	11231.64	-4.67565	0.656416	-7.12299	1.06E-12	5.39E-10	TFPI2	7980	tissue factor pathway inhibitor 2
ENSG0000	7591.908	-4.016	0.569008	-7.0579	1.69E-12	8.41E-10	ITGA2	3673	integrin subunit alpha 2
ENSG0000	153.0336	4.345876	0.617373	7.039303	1.93E-12	9.37E-10	ZNF423	23090	zinc finger protein 423
ENSG0000	1426.128	-6.65393	0.949208	-7.00998	2.38E-12	1.13E-09	CXCL3	2921	C-X-C motif chemokine ligand 3
ENSG0000	28.78937	-4.70407	0.67274	-6.99241	2.70E-12	1.25E-09	RCSD1	92241	RCSD domain containing 1
ENSG0000	1550.904	-2.35151	0.33712	-6.97528	3.05E-12	1.35E-09	NAB1	4664	NGFI-A binding protein 1
ENSG0000	907.6342	-4.47506	0.641647	-6.97433	3.07E-12	1.35E-09	IFIT2	3433	interferon induced protein with tetratricopeptide repeats 2
ENSG0000	349.827	-2.10351	0.304402	-6.91031	4.84E-12	2.08E-09	NOTCH2N	1.01E+08	notch 2 N-terminal like C
ENSG0000	94.44857	3.622982	0.527575	6.867235	6.55E-12	2.76E-09	NA	NA	NA
ENSG0000	7492.032	1.817056	0.265111	6.853939	7.18E-12	2.94E-09	VGLL3	389136	vestigial like family member 3
ENSG0000	1277.097	-2.91254	0.425596	-6.84342	7.73E-12	3.06E-09	ZC3H12C	85463	zinc finger CCCH-type containing 12C
ENSG0000	965.8331	7.179144	1.054235	6.809816	9.77E-12	3.79E-09	AQP1	358	aquaporin 1 (Colton blood group)
ENSG0000	813.8304	3.196494	0.470921	6.787754	1.14E-11	4.33E-09	NA	NA	NA
ENSG0000	2022.749	-5.64576	0.834261	-6.76738	1.31E-11	4.89E-09	MX1	4599	MX dynamin like GTPase 1
ENSG0000	111405.3	3.116195	0.466277	6.683144	2.34E-11	8.56E-09	COL3A1	1281	collagen type III alpha 1 chain
ENSG0000	2247.311	-1.61976	0.243324	-6.65679	2.80E-11	1.01E-08	JMJD1C	221037	jumonji domain containing 1C
ENSG0000	768.6769	2.030174	0.307762	6.596579	4.21E-11	1.47E-08	PEX6	5190	peroxisomal biogenesis factor 6
ENSG0000	888.7781	-1.72018	0.260836	-6.59488	4.26E-11	1.47E-08	PIK3CD	5293	phosphatidylinositol-4,5-bisphosphate 3-kinase catalytic sub unit delta
ENSG0000	1260.684	2.311246	0.351251	6.580036	4.70E-11	1.60E-08	NA	NA	NA
ENSG0000	503.6104	2.633321	0.401113	6.565029	5.20E-11	1.74E-08	IRX5	10265	iroquois homeobox 5
ENSG0000	208.5104	5.994375	0.921696	6.503637	7.84E-11	2.53E-08	CMKLR1	1240	chemerin chemokine-like receptor 1
ENSG0000	1065.522	-2.38012	0.367791	-6.47139	9.71E-11	3.09E-08	FAM167A	83648	family with sequence similarity 167 member A
ENSG0000	6026.42	1.940108	0.301207	6.441117	1.19E-10	3.71E-08	MTCO3P1	1.07E+08	MT-CO3 pseudogene 12
ENSG0000	1614.308	-1.52709	0.237621	-6.42658	1.31E-10	4.02E-08	CDK14	5218	cyclin dependent kinase 14
ENSG0000	7626.214	-2.4811	0.387606	-6.40109	1.54E-10	4.68E-08	SLC39A14	23516	solute carrier family 39 member 14
ENSG0000	523.0037	2.846645	0.447549	6.360517	2.01E-10	5.97E-08	RAB7B	338382	RAB7B, member RAS oncogene family
ENSG0000	362693.7	2.979077	0.468495	6.358823	2.03E-10	5.97E-08	COL1A1	1277	collagen type I alpha 1 chain
ENSG0000	6313.679	-1.87998	0.296918	-6.33164	2.43E-10	7.02E-08	ELK3	2004	ETS transcription factor ELK3
ENSG0000	586.106	-3.81362	0.602971	-6.32471	2.54E-10	7.24E-08	IRAK2	3656	interleukin 1 receptor associated kinase 2
ENSG0000	411.6157	-2.6976	0.427927	-6.30389	2.90E-10	8.16E-08	SLC22A4	6583	solute carrier family 22 member 4
ENSG0000	1965.025	-2.13094	0.338918	-6.28746	3.23E-10	8.94E-08	STEAP1	26872	STEAP family member 1
ENSG0000	1216.328	-1.69657	0.270039	-6.28269	3.33E-10	9.09E-08	RASA2	5922	RAS p21 protein activator 2
ENSG0000	4650.984	-2.23845	0.357476	-6.26182	3.81E-10	1.03E-07	RASSF8	11228	Ras association domain family member 8
ENSG0000	2627.732	1.552992	0.249694	6.219574	4.99E-10	1.32E-07	TNS2	23371	tensin 2
ENSG0000	301.2517	2.985994	0.480403	6.215602	5.11E-10	1.34E-07	NA	NA	NA
ENSG0000	203.4271	-3.65231	0.590197	-6.18829	6.08E-10	1.57E-07	RNF144B	255488	ring finger protein 144B
ENSG0000	192.2146	3.335121	0.5393	6.184162	6.24E-10	1.59E-07	MIR1915H	399726	MIR1915 host gene
ENSG0000	1589.535	5.223121	0.848797	6.15356	7.58E-10	1.91E-07	RCAN2	10231	regulator of calcineurin 2
ENSG0000	127.564	2.722023	0.44257	6.150493	7.72E-10	1.92E-07	GUCY1B1	2983	guanylate cyclase 1 soluble subunit beta 1
ENSG0000	6913.84	3.121612	0.508163	6.142934	8.10E-10	1.99E-07	FHL1	2273	four and a half LIM domains 1
ENSG0000	816.1934	-1.58501	0.259592	-6.10577	1.02E-09	2.48E-07	NR1D1	9572	nuclear receptor subfamily 1 group D member 1
ENSG0000	324.4863	3.548112	0.58583	6.05656	1.39E-09	3.33E-07	DIRAS3	9077	DIRAS family GTPase 3
ENSG0000	265.4811	7.438376	1.23266	6.03441	1.60E-09	3.74E-07	VCAM1	7412	vascular cell adhesion molecule 1
ENSG0000	670.1037	-2.95277	0.489527	-6.03188	1.62E-09	3.74E-07	ADGRL4	64123	adhesion G protein-coupled receptor L4
ENSG0000	1361.864	1.564247	0.259333	6.031814	1.62E-09	3.74E-07	RPS2P7	86781	ribosomal protein S2 pseudogene 7
ENSG0000	171.2698	-2.85212	0.474637	-6.00907	1.87E-09	4.26E-07	NA	NA	NA
ENSG0000	149.0458	1.990744	0.332797	5.981849	2.21E-09	4.98E-07	ERV3-1	2086	endogenous retrovirus group 3 member 1, envelope
ENSG0000	3245.125	-4.52274	0.757678	-5.96921	2.38E-09	5.25E-07	IFIT3	3437	interferon induced protein with tetratricopeptide repeats 3
ENSG0000	1070.626	2.718251	0.457347	5.943518	2.79E-09	6.08E-07	SORCS2	57537	sortilin related VPS10 domain containing receptor 2
ENSG0000	1780.707	-2.49862	0.421049	-5.93428	2.95E-09	6.36E-07	FAM180A	389558	family with sequence similarity 180 member A
ENSG0000	87.19447	4.584505	0.774009	5.923064	3.16E-09	6.74E-07	SOX8	30812	SRY-box transcription factor 8
ENSG0000	7959.94	-3.7809	0.63868	-5.91986	3.22E-09	6.79E-07	HAS2	3037	hyaluronan synthase 2
ENSG0000	1320.852	6.874068	1.163252	5.909352	3.43E-09	7.16E-07	IRAG1	10335	inositol 1,4,5-triphosphate receptor associated 1
ENSG0000	2496.574	7.047558	1.193063	5.907112	3.48E-09	7.18E-07	CCN5	8839	cellular communication network factor 5
ENSG0000	2010.406	-1.76913	0.299655	-5.9039	3.55E-09	7.25E-07	PPIF	10105	peptidylprolyl isomerase F
ENSG0000	2750.921	-1.61096	0.273039	-5.9001	3.63E-09	7.27E-07	SRGAP1	57522	SLIT-ROBO Rho GTPase activating protein 1

ENSG0000	28.58619	-6.18392	1.048128	-5.89997	3.64E-09	7.27E-07	ACP7	390928	acid phosphatase 7, tartrate resistant (putative)
ENSG0000	78.27323	-4.07446	0.692292	-5.88548	3.97E-09	7.86E-07	NA	NA	NA
ENSG0000	1548.426	-1.62025	0.275621	-5.87855	4.14E-09	8.02E-07	USP12	219333	ubiquitin specific peptidase 12
ENSG0000	47.84153	-7.61246	1.295005	-5.87832	4.14E-09	8.02E-07	EPGN	255324	epithelial mitogen
ENSG0000	57.95545	-2.53909	0.432028	-5.87713	4.17E-09	8.02E-07	NA	NA	NA
ENSG0000	208.7023	3.42278	0.582925	5.871729	4.31E-09	8.20E-07	EEPD1	80820	endonuclease/exonuclease/phosphatase family domain con aining 1
ENSG0000	603.7238	1.664034	0.283682	5.865839	4.47E-09	8.41E-07	SMARCD3	6604	SWI/SNF related, matrix associated, actin dependent regula tor of chromatin, subfamily d, member 3
ENSG0000	162.0974	-4.58407	0.782196	-5.86052	4.61E-09	8.61E-07	WTAPP1	1E+08	Wilms tumor 1 associated protein pseudogene 1
ENSG0000	11236.48	2.252487	0.384581	5.856988	4.71E-09	8.71E-07	MXRA8	54587	matrix remodeling associated 8
ENSG0000	510.7038	2.612881	0.447631	5.837132	5.31E-09	9.72E-07	EPHB3	2049	EPH receptor B3
ENSG0000	2181.469	-2.51579	0.431652	-5.82828	5.60E-09	1.02E-06	STEAP2	261729	STEAP2 metalloreductase
ENSG0000	929.5504	-4.59562	0.78934	-5.8221	5.81E-09	1.04E-06	HERC6	55008	HECT and RLD domain containing E3 ubiquitin protein ligase family member 6
ENSG0000	2134.035	-3.83786	0.65971	-5.8175	5.97E-09	1.06E-06	TNFAIP3	7128	TNF alpha induced protein 3
ENSG0000	608.1858	-2.80961	0.48324	-5.81412	6.10E-09	1.07E-06	GK	2710	glycerol kinase
ENSG0000	7109.319	-6.13981	1.061102	-5.78626	7.20E-09	1.26E-06	CXCL1	2919	C-X-C motif chemokine ligand 1
ENSG0000	1497.039	-1.52322	0.263537	-5.77989	7.48E-09	1.29E-06	TUT7	79670	terminal uridylyl transferase 7
ENSG0000	471.4712	-1.60971	0.278858	-5.77251	7.81E-09	1.34E-06	STX3	6809	syntaxin 3
ENSG0000	457.4731	1.792259	0.310655	5.769285	7.96E-09	1.35E-06	SHISA4	149345	shisa family member 4
ENSG0000	153.0274	-4.70443	0.816291	-5.76318	8.25E-09	1.39E-06	KDR	3791	kinase insert domain receptor
ENSG0000	382.7588	-3.16454	0.550424	-5.74928	8.96E-09	1.50E-06	BDKRB1	623	bradykinin receptor B1
ENSG0000	133.6896	1.928669	0.335931	5.741269	9.40E-09	1.56E-06	PCDHGA2	56113	protocadherin gamma subfamily A, 2
ENSG0000	421.2459	-2.22578	0.388265	-5.73262	9.89E-09	1.61E-06	SYNE2	23224	spectrin repeat containing nuclear envelope protein 2
ENSG0000	554.6798	6.923275	1.207748	5.732383	9.90E-09	1.61E-06	CLEC3B	7123	C-type lectin domain family 3 member B
ENSG0000	43920.76	3.183188	0.556281	5.722267	1.05E-08	1.68E-06	IGFBP4	3487	insulin like growth factor binding protein 4
ENSG0000	281.5728	6.718055	1.174032	5.722206	1.05E-08	1.68E-06	COL21A1	81578	collagen type XXI alpha 1 chain
ENSG0000	11859.47	2.274125	0.397446	5.721842	1.05E-08	1.68E-06	PCDH18	54510	protocadherin 18
ENSG0000	380.1146	-4.66268	0.815139	-5.7201	1.06E-08	1.68E-06	IL1RN	3557	interleukin 1 receptor antagonist
ENSG0000	58.65115	-4.45649	0.781426	-5.70302	1.18E-08	1.84E-06	NA	NA	NA
ENSG0000	7080.654	-2.21775	0.389262	-5.69731	1.22E-08	1.89E-06	ATP13A3	79572	ATPase 13A3
ENSG0000	620.8264	-2.56674	0.450612	-5.69611	1.23E-08	1.89E-06	PKIA	5569	cAMP-dependent protein kinase inhibitor alpha
ENSG0000	314.5929	3.204325	0.56282	5.693334	1.25E-08	1.90E-06	PODNL1	79883	podocan like 1
ENSG0000	912.8341	-3.5966	0.631979	-5.69102	1.26E-08	1.91E-06	ICAM1	3383	intercellular adhesion molecule 1
ENSG0000	782.4684	-3.59428	0.632277	-5.68465	1.31E-08	1.97E-06	FZD8	8325	frizzled class receptor 8
ENSG0000	217.814	5.29464	0.93321	5.673581	1.40E-08	2.09E-06	MAP2K6	5608	mitogen-activated protein kinase kinase 6
ENSG0000	4939.82	1.697591	0.299297	5.671928	1.41E-08	2.09E-06	SDC3	9672	syndecan 3
ENSG0000	334.099	-2.16751	0.382384	-5.66842	1.44E-08	2.12E-06	BCL7A	605	BAF chromatin remodeling complex subunit BCL7A
ENSG0000	419.0386	3.32147	0.587423	5.654312	1.56E-08	2.28E-06	PLEKHA6	22874	pleckstrin homology domain containing A6
ENSG0000	552.5362	2.028557	0.359152	5.648184	1.62E-08	2.35E-06	SMAD6	4091	SMAD family member 6
ENSG0000	208.1331	3.884714	0.689047	5.637809	1.72E-08	2.47E-06	NA	NA	NA
ENSG0000	614.6565	1.819656	0.323828	5.619204	1.92E-08	2.74E-06	TMEM8B	51754	transmembrane protein 8B
ENSG0000	6262.492	4.706641	0.838369	5.614046	1.98E-08	2.80E-06	PTGIS	5740	prostaglandin I2 synthase
ENSG0000	846.5349	4.76009	0.849431	5.603859	2.10E-08	2.95E-06	GPRC5B	51704	G protein-coupled receptor class C group 5 member B
ENSG0000	1208.826	-1.81157	0.324367	-5.58495	2.34E-08	3.26E-06	OGFRL1	79627	opioid growth factor receptor like 1
ENSG0000	1892.63	-4.08789	0.733982	-5.56947	2.56E-08	3.54E-06	RIGI	23586	RNA sensor RIG-I
ENSG0000	335.1264	-2.0514	0.36894	-5.56024	2.69E-08	3.69E-06	TMEM38B	55151	transmembrane protein 38B
ENSG0000	1024.076	-1.60627	0.289484	-5.54873	2.88E-08	3.90E-06	OSTM1	28962	osteoclastogenesis associated transmembrane protein 1
ENSG0000	1962.231	-4.58504	0.82731	-5.54211	2.99E-08	4.03E-06	IL11	3589	interleukin 11
ENSG0000	1161.6	-2.44173	0.441866	-5.52595	3.28E-08	4.38E-06	PITPNC1	26207	phosphatidylinositol transfer protein cytoplasmic 1
ENSG0000	10893.84	1.989618	0.360133	5.524668	3.30E-08	4.38E-06	ISLR	3671	immunoglobulin superfamily containing leucine rich repeat
ENSG0000	36.85995	-6.53792	1.183584	-5.52383	3.32E-08	4.38E-06	NA	NA	NA
ENSG0000	827.9129	-2.27555	0.412174	-5.52085	3.37E-08	4.41E-06	SLC43A3	29015	solute carrier family 43 member 3
ENSG0000	20.91888	-8.49499	1.53893	-5.52006	3.39E-08	4.41E-06	SERPINB4	6318	serpin family B member 4
ENSG0000	1082.561	-3.29313	0.597199	-5.51429	3.50E-08	4.53E-06	AMPD3	272	adenosine monophosphate deaminase 3
ENSG0000	92.78829	-2.78203	0.508223	-5.47403	4.40E-08	5.65E-06	GPR3	2827	G protein-coupled receptor 3
ENSG0000	11486.88	1.845062	0.337169	5.472213	4.44E-08	5.67E-06	FBLN5	10516	fibulin 5
ENSG0000	488.5641	5.378301	0.983392	5.469132	4.52E-08	5.71E-06	ECM2	1842	extracellular matrix protein 2
ENSG0000	43.98496	3.126994	0.571813	5.468561	4.54E-08	5.71E-06	NA	NA	NA
ENSG0000	15338.22	1.782492	0.327007	5.450922	5.01E-08	6.27E-06	MTATP6P	1.06E+08	MT-ATP6 pseudogene 1
ENSG0000	147.1733	-4.97263	0.913495	-5.44352	5.22E-08	6.49E-06	IL1A	3552	interleukin 1 alpha
ENSG0000	237.2657	-1.61718	0.297185	-5.44167	5.28E-08	6.52E-06	CGAS	115004	cyclic GMP-AMP synthase
ENSG0000	706.2378	2.050467	0.377284	5.434814	5.49E-08	6.69E-06	TRIB2	28951	tribbles pseudokinase 2
ENSG0000	493.682	2.20487	0.406262	5.427209	5.72E-08	6.94E-06	RPS2P55	286444	ribosomal protein S2 pseudogene 55
ENSG0000	200.8441	-6.04468	1.114328	-5.42451	5.81E-08	7.00E-06	HERC5	51191	HECT and RLD domain containing E3 ubiquitin protein ligase 5
ENSG0000	179.7517	7.927756	1.465703	5.408843	6.34E-08	7.59E-06	TRIL	9865	TLR4 interactor with leucine rich repeats
ENSG0000	19.25483	-6.92244	1.282479	-5.39771	6.75E-08	7.97E-06	IVL	3713	involucrin
ENSG0000	966.0746	-2.9113	0.539406	-5.39724	6.77E-08	7.97E-06	PDCD1LG2	80380	programmed cell death 1 ligand 2
ENSG0000	74.43011	2.217427	0.410871	5.396898	6.78E-08	7.97E-06	RPS2P8	440589	ribosomal protein S2 pseudogene 8
ENSG0000	7669.592	-2.6519	0.491944	-5.39065	7.02E-08	8.20E-06	CEP170	9859	centrosomal protein 170
ENSG0000	197.0703	2.975688	0.55303	5.380701	7.42E-08	8.59E-06	IER5L	389792	immediate early response 5 like
ENSG0000	477.394	2.577582	0.479128	5.379739	7.46E-08	8.59E-06	MAB21L1	4081	mab-21 like 1
ENSG0000	51606.95	1.974446	0.367055	5.379162	7.48E-08	8.59E-06	COX3	4514	cytochrome c oxidase subunit III
ENSG0000	107.608	-2.02464	0.377574	-5.36223	8.22E-08	9.38E-06	MARCHF3	115123	membrane associated ring-CH-type finger 3
ENSG0000	127.4317	-6.19137	1.156604	-5.35306	8.65E-08	9.81E-06	C15orf48	84419	chromosome 15 open reading frame 48
ENSG0000	2499.744	-1.73753	0.324869	-5.3484	8.87E-08	1.00E-05	GSTO1	9446	glutathione S-transferase omega 1
ENSG0000	186.1033	-4.38623	0.82224	-5.33449	9.58E-08	1.07E-05	PPP4R4	57718	protein phosphatase 4 regulatory subunit 4
ENSG0000	155.2325	-5.05611	0.947837	-5.33437	9.59E-08	1.07E-05	ADTRP	84830	androgen dependent TFPI regulating protein
ENSG0000	813.834	-3.90813	0.733674	-5.3268	1.00E-07	1.11E-05	GDNF	2668	glial cell derived neurotrophic factor
ENSG0000	335.5006	-2.77171	0.52062	-5.32387	1.02E-07	1.12E-05	KCNJ15	3772	potassium inwardly rectifying channel subfamily J member 15
ENSG0000	1445.491	-1.82726	0.343983	-5.31206	1.08E-07	1.19E-05	DUSP10	11221	dual specificity phosphatase 10
ENSG0000	1468.83	6.821042	1.286211	5.303207	1.14E-07	1.24E-05	OLFML2A	169611	olfactomedin like 2A
ENSG0000	36.93723	-4.25442	0.802684	-5.30025	1.16E-07	1.25E-05	TMCC3	57458	transmembrane and coiled-coil domain family 3
ENSG0000	277.0177	4.028306	0.760463	5.297175	1.18E-07	1.27E-05	BMF	90427	Bcl2 modifying factor
ENSG0000	2438.323	-2.79465	0.527946	-5.29344	1.20E-07	1.28E-05	ITPRIP	85450	inositol 1,4,5-trisphosphate receptor interacting protein
ENSG0000	1049.346	-5.4755	1.034502	-5.29289	1.20E-07	1.28E-05	CXCL2	2920	C-X-C motif chemokine ligand 2
ENSG0000	2449.07	-4.70946	0.890289	-5.28981	1.22E-07	1.30E-05	ISG15	9636	ISG15 ubiquitin like modifier
ENSG0000	974.5155	-1.80436	0.341322	-5.28637	1.25E-07	1.32E-05	IRAK3	11213	interleukin 1 receptor associated kinase 3
ENSG0000	221.4948	-2.14082	0.406119	-5.27139	1.35E-07	1.41E-05	NOTCH2N	388677	notch 2 N-terminal like A
ENSG0000	203.2792	2.748772	0.521494	5.270952	1.36E-07	1.41E-05	PCDHGA7	56108	protocadherin gamma subfamily A, 7
ENSG0000	309.3064	2.175202	0.415404	5.236353	1.64E-07	1.68E-05	TSPOAP1	9256	TSPO associated protein 1
ENSG0000	213.4245	1.628126	0.311153	5.232553	1.67E-07	1.71E-05	OSBPL7	114881	oxysterol binding protein like 7
ENSG0000	14673.71	-3.66303	0.700458	-5.22947	1.70E-07	1.73E-05	STC1	6781	stanniocalcin 1
ENSG0000	243.9768	1.911898	0.365763	5.227144	1.72E-07	1.74E-05	CORO6	84940	coronin 6
ENSG0000	44.07743	4.304517	0.824104	5.223268	1.76E-07	1.77E-05	LINC00842	643650	long intergenic non-protein coding RNA 842
ENSG0000	1771.066	-4.66208	0.895384	-5.2068	1.92E-07	1.92E-05	OAS2	4939	2'-5'-oligoadenylate synthetase 2

ENSG0000	3183.818	2.108196	0.405083	5.204352	1.95E-07	1.94E-05	SSC5D	284297	scavenger receptor cysteine rich family member with 5 dom ains
ENSG0000	1053.543	-2.35148	0.452447	-5.19725	2.02E-07	2.00E-05	FBXO32	114907	F-box protein 32
ENSG0000	33.4723	-7.73193	1.490568	-5.18724	2.13E-07	2.10E-05	MUC13	56667	mucin 13, cell surface associated
ENSG0000	96.03064	-4.78508	0.923046	-5.18401	2.17E-07	2.13E-05	PTPN22	26191	protein tyrosine phosphatase non-receptor type 22
ENSG0000	881.8898	-5.89147	1.137604	-5.17884	2.23E-07	2.18E-05	IFI44L	10964	interferon induced protein 44 like
ENSG0000	355.5621	3.663582	0.708403	5.171607	2.32E-07	2.25E-05	NA	NA	NA
ENSG0000	2757.135	3.878189	0.751944	5.157548	2.50E-07	2.40E-05	PODN	127435	podocan
ENSG0000	2349.456	-5.95561	1.15476	-5.15745	2.50E-07	2.40E-05	CXCL6	6372	C-X-C motif chemokine ligand 6
ENSG0000	165.5569	2.775147	0.538247	5.1559	2.52E-07	2.41E-05	LOC72839	728392	uncharacterized LOC728392
ENSG0000	84.18152	-5.50176	1.067488	-5.15394	2.55E-07	2.43E-05	NA	NA	NA
ENSG0000	108.4969	-7.38842	1.43502	-5.14865	2.62E-07	2.48E-05	CCL5	6352	C-C motif chemokine ligand 5
ENSG0000	2037.784	1.927662	0.374452	5.147958	2.63E-07	2.48E-05	TSHZ1	10194	teashirt zinc finger homeobox 1
ENSG0000	207.9726	-4.54869	0.884094	-5.14503	2.67E-07	2.51E-05	ISG20	3669	interferon stimulated exonuclease gene 20

Supp Table 6 DDC and WNT4siRNA vs NS comparison

ENSG	baseMean	log2FoldC	lfcSE	stat	pvalue	padj	symbol	entrez	genename
ENSG0000	7092.185	-2.95219	0.153863	-19.1872	4.74E-82	1.26E-77	JUND	3727	JunD proto-oncogene, AP-1 transcription factor subunit
ENSG0000	3687.573	-4.03054	0.244854	-16.461	6.99E-61	9.33E-57	PER1	5187	period circadian regulator 1
ENSG0000	6660.578	-2.59509	0.16516	-15.7126	1.24E-55	1.10E-51	MIDN	90007	midnolin
ENSG0000	3929.167	-3.6428	0.246894	-14.7545	2.88E-49	1.92E-45	CSRNP1	64651	cysteine and serine rich nuclear protein 1
ENSG0000	164.4153	6.301101	0.448211	14.05834	6.85E-45	3.04E-41	HOXD10	3236	homeobox D10
ENSG0000	5379.871	-4.79807	0.357314	-13.4281	4.14E-41	1.58E-37	HES1	3280	hes family bHLH transcription factor 1
ENSG0000	7525.247	-2.74704	0.209049	-13.1406	1.93E-39	4.67E-36	KLF10	7071	KLF transcription factor 10
ENSG0000	2252.277	-3.32323	0.255814	-12.9908	1.38E-38	3.07E-35	NA	NA	NA
ENSG0000	2253.087	1.924225	0.151369	12.71213	5.06E-37	9.65E-34	SH3KBP1	30011	SH3 domain containing kinase binding protein 1
ENSG0000	462.3468	1.917948	0.152885	12.54504	4.23E-36	7.53E-33	MRTFB	57496	myocardin related transcription factor B
ENSG0000	253.6934	1.568032	0.125943	12.45037	1.39E-35	2.32E-32	GSPT2	23708	G1 to S phase transition 2
ENSG0000	11579.98	-4.92605	0.39709	-12.4054	2.44E-35	3.84E-32	BHLHE40	8553	basic helix-loop-helix family member e40
ENSG0000	46.20752	-4.97305	0.404546	-12.2929	9.89E-35	1.32E-31	HOXC-AS2	100874364	HOXC cluster antisense RNA 2
ENSG0000	85.40483	-4.0215	0.329397	-12.2087	2.79E-34	3.55E-31	NA	NA	NA
ENSG0000	67.24331	-3.36031	0.27655	-12.1508	5.68E-34	6.89E-31	NA	NA	NA
ENSG0000	85.01013	-4.01758	0.335618	-11.9707	5.06E-33	5.87E-30	NA	NA	NA
ENSG0000	859.9455	1.800857	0.152477	11.81065	3.44E-32	3.82E-29	PPARA	5465	peroxisome proliferator activated receptor alpha
ENSG0000	72.13668	5.694354	0.482513	11.80146	3.84E-32	4.09E-29	HOXD11	3237	homeobox D11
ENSG0000	8624.911	2.331578	0.198289	11.75846	6.39E-32	6.56E-29	CD109	135228	CD109 molecule
ENSG0000	97.97632	-10.1206	0.862289	-11.7369	8.25E-32	8.15E-29	HOXB8	3218	homeobox B8
ENSG0000	220.6859	-10.3286	0.914281	-11.2969	1.36E-29	1.25E-26	HOXB-AS3	404266	HOXB cluster antisense RNA 3
ENSG0000	324.6969	-2.76053	0.245187	-11.2589	2.09E-29	1.86E-26	HSD17B14	51171	hydroxysteroid 17-beta dehydrogenase 14
ENSG0000	83.61923	-5.7925	0.514721	-11.2537	2.22E-29	1.91E-26	NA	NA	NA
ENSG0000	247.6473	1.655776	0.148322	11.16341	6.16E-29	4.98E-26	KBTBD6	89890	kelch repeat and BTB domain containing 6
ENSG0000	1658.007	-1.66432	0.150788	-11.0375	2.52E-28	1.98E-25	NEU1	4758	neuraminidase 1
ENSG0000	3454.408	-3.31453	0.300697	-11.0229	2.97E-28	2.26E-25	PIM1	5292	Pim-1 proto-oncogene, serine/threonine kinase
ENSG0000	3596.02	1.868287	0.169727	11.00757	3.51E-28	2.60E-25	PTPN13	5783	protein tyrosine phosphatase non-receptor type 13
ENSG0000	87447.84	-2.33129	0.212542	-10.9686	5.41E-28	3.90E-25	ND2	4536	NADH dehydrogenase subunit 2
ENSG0000	1253.448	-4.93851	0.451127	-10.9471	6.86E-28	4.82E-25	ATF3	467	activating transcription factor 3
ENSG0000	189.2784	-10.5889	0.968528	-10.9329	8.02E-28	5.49E-25	HES5	388585	hes family bHLH transcription factor 5
ENSG0000	174.0571	-9.50512	0.878262	-10.8227	2.69E-27	1.75E-24	LEP	3952	leptin
ENSG0000	1110.343	-3.10203	0.288977	-10.7345	7.01E-27	4.45E-24	MAP3K8	1326	mitogen-activated protein kinase kinase kinase 8
ENSG0000	215.6158	1.723243	0.1643	10.48841	9.77E-26	5.43E-23	PDP2	57546	pyruvate dehydrogenase phosphatase catalytic subunit 2
ENSG0000	580.283	2.959255	0.283089	10.45346	1.41E-25	7.69E-23	INSYN2B	100131897	inhibitory synaptic factor family member 2B
ENSG0000	86.68672	-3.8656	0.369958	-10.4487	1.48E-25	7.83E-23	NA	NA	NA
ENSG0000	40066.09	-2.56735	0.246678	-10.4077	2.29E-25	1.17E-22	MTND2P2	100652939	MT-ND2 pseudogene 28
ENSG0000	285.2176	-6.58902	0.634827	-10.3792	3.08E-25	1.52E-22	NA	NA	NA
ENSG0000	96.37068	-3.82912	0.371764	-10.2999	7.06E-25	3.36E-22	NA	NA	NA
ENSG0000	807.581	-1.56595	0.153543	-10.1988	2.01E-24	9.23E-22	PTS	5805	6-pyruvoyltetrahydropterin synthase
ENSG0000	1726.975	-3.78825	0.373999	-10.129	4.11E-24	1.83E-21	TRIB1	10221	tribbles pseudokinase 1
ENSG0000	1340.141	-2.37635	0.236935	-10.0295	1.13E-23	4.79E-21	GDPD5	81544	glycerophosphodiester phosphodiesterase domain containing 5
ENSG0000	636.5039	-1.98502	0.1982	-10.0152	1.31E-23	5.45E-21	DHRS7B	25979	dehydrogenase/reductase 7B
ENSG0000	241.6855	-1.76426	0.176313	-10.0064	1.43E-23	5.86E-21	NA	NA	NA
ENSG0000	339.3983	-4.059	0.408942	-9.92562	3.22E-23	1.28E-20	TNFSF9	8744	TNF superfamily member 9
ENSG0000	1172.894	-4.29376	0.434961	-9.87161	5.53E-23	2.14E-20	SIK1	150094	salt inducible kinase 1
ENSG0000	2575.176	2.052588	0.208091	9.863874	5.97E-23	2.28E-20	ITPRID2	6744	ITPR interacting domain containing 2
ENSG0000	127548.3	1.891958	0.193015	9.802139	1.10E-22	4.08E-20	COL6A3	1293	collagen type VI alpha 3 chain
ENSG0000	1295.753	-4.31393	0.442368	-9.7519	1.81E-22	6.62E-20	LOC10272	102724428	salt inducible kinase 1B (putative)
ENSG0000	1408.029	-1.60236	0.164493	-9.74119	2.01E-22	7.16E-20	JMJD6	23210	jumonji domain containing 6, arginine demethylase and lysine hy roxylase
ENSG0000	355.0853	-5.10984	0.527317	-9.69027	3.32E-22	1.13E-19	USP2	9099	ubiquitin specific peptidase 2
ENSG0000	111591.1	-2.36731	0.244716	-9.67367	3.90E-22	1.32E-19	CYTB	4519	cytochrome b
ENSG0000	42.4667	-3.22266	0.333905	-9.65141	4.85E-22	1.62E-19	NA	NA	NA
ENSG0000	2446.155	-1.67187	0.174038	-9.60631	7.52E-22	2.47E-19	DDAH2	23564	dimethylarginine dimethylaminohydrolase 2
ENSG0000	1047.191	1.655974	0.174315	9.499873	2.10E-21	6.60E-19	TBL1X	6907	transducin beta like 1 X-linked
ENSG0000	554.3629	-1.62649	0.172326	-9.43846	3.78E-21	1.17E-18	ZNF408	79797	zinc finger protein 408
ENSG0000	71.81072	-5.34691	0.567053	-9.4293	4.13E-21	1.27E-18	NA	NA	NA
ENSG0000	126.9158	-1.62568	0.173687	-9.35984	7.99E-21	2.34E-18	ZNF433	163059	zinc finger protein 433
ENSG0000	666.0285	-5.23011	0.559046	-9.35542	8.33E-21	2.41E-18	HEY1	23462	hes related family bHLH transcription factor with YRPW motif 1
ENSG0000	2055.557	14.40432	1.541457	9.344614	9.22E-21	2.65E-18	RPS4Y1	6192	ribosomal protein S4 Y-linked 1
ENSG0000	225.8776	-1.60284	0.171632	-9.33883	9.74E-21	2.76E-18	FAM89B	23625	family with sequence similarity 89 member B
ENSG0000	6938.828	-1.96071	0.210065	-9.33382	1.02E-20	2.87E-18	PPP1R15A	23645	protein phosphatase 1 regulatory subunit 15A
ENSG0000	249.9822	7.396459	0.792883	9.328568	1.07E-20	2.98E-18	ZFY	7544	zinc finger protein Y-linked
ENSG0000	22442.19	-1.59892	0.171877	-9.30269	1.37E-20	3.73E-18	CDKN1A	1026	cyclin dependent kinase inhibitor 1A
ENSG0000	189.4332	-3.76859	0.407233	-9.25413	2.16E-20	5.76E-18	IDI2-AS1	55853	IDI2 antisense RNA 1
ENSG0000	25.62998	-3.78014	0.410549	-9.20753	3.34E-20	8.69E-18	NA	NA	NA
ENSG0000	393.6198	5.885268	0.639222	9.206922	3.36E-20	8.69E-18	HOXA13	3209	homeobox A13
ENSG0000	877.6898	-1.53523	0.166869	-9.20023	3.57E-20	9.08E-18	SLC25A25	114789	solute carrier family 25 member 25
ENSG0000	494.8073	1.680036	0.183485	9.156237	5.37E-20	1.34E-17	CRYBG3	131544	crystallin beta-gamma domain containing 3
ENSG0000	11850.54	-3.83091	0.419374	-9.13481	6.55E-20	1.62E-17	GADD45B	4616	growth arrest and DNA damage inducible beta
ENSG0000	22184.14	-2.17906	0.240104	-9.07547	1.13E-19	2.77E-17	ND4L	4539	NADH dehydrogenase subunit 4L
ENSG0000	979.0427	-1.93446	0.213735	-9.0507	1.42E-19	3.45E-17	TMSB4XP4	7118	TMSB4X pseudogene 4
ENSG0000	269.2674	8.144297	0.902521	9.023941	1.81E-19	4.36E-17	PRKY	5616	protein kinase Y-linked (pseudogene)
ENSG0000	1299.56	-3.03353	0.336351	-9.01894	1.90E-19	4.52E-17	RNA5-8SN	109910381	RNA, 5.8S ribosomal N3
ENSG0000	103.6433	5.765136	0.639362	9.017016	1.93E-19	4.56E-17	GALNT18	374378	polypeptide N-acetylgalactosaminyltransferase 18
ENSG0000	1298.936	-3.03275	0.336537	-9.01165	2.03E-19	4.75E-17	RNA5-8SN	109864281	RNA, 5.8S ribosomal N2
ENSG0000	1298.403	-3.03383	0.337242	-8.99601	2.34E-19	5.43E-17	RNA5-8SN	106632260	RNA, 5.8S ribosomal N1
ENSG0000	171.548	1.621782	0.180386	8.990602	2.46E-19	5.61E-17	ZBTB39	9880	zinc finger and BTB domain containing 39
ENSG0000	1297.78	-3.03305	0.337427	-8.98875	2.50E-19	5.61E-17	LOC12490	124900510	5.8S ribosomal RNA
ENSG0000	1297.78	-3.03305	0.337427	-8.98875	2.50E-19	5.61E-17	LOC12490	124900510	5.8S ribosomal RNA
ENSG0000	1297.78	-3.03305	0.337427	-8.98875	2.50E-19	5.61E-17	RNA5-8SP	109910384	RNA, 5.8S ribosomal pseudogene 10
ENSG0000	1297.504	-3.03271	0.337489	-8.98609	2.56E-19	5.65E-17	RNA5-8SN	100008587	RNA, 5.8S ribosomal N5
ENSG0000	1297.504	-3.03271	0.337489	-8.98609	2.56E-19	5.65E-17	RNA5-8SN	109864274	RNA, 5.8S ribosomal N4

ENSG0000	502.2939	-3.58579	0.39921	-8.98221	2.65E-19	5.80E-17	CHRD	8646	chordin
ENSG0000	955.0993	1.501076	0.168926	8.885997	6.34E-19	1.33E-16	GTF3C4	9329	general transcription factor IIIC subunit 4
ENSG0000	26.6834	-5.35569	0.604877	-8.85417	8.43E-19	1.74E-16	LOC10537	105370047	uncharacterized LOC105370047
ENSG0000	96.9312	-2.7406	0.309588	-8.85242	8.56E-19	1.76E-16	CD14	929	CD14 molecule
ENSG0000	1529.119	-2.69878	0.30514	-8.8444	9.20E-19	1.87E-16	CLCF1	23529	cardiotrophin like cytokine factor 1
ENSG0000	2444.189	2.117748	0.240581	8.802636	1.34E-18	2.70E-16	SYNJ2	8871	synaptojanin 2
ENSG0000	758.1371	1.622454	0.185227	8.759251	1.97E-18	3.88E-16	ASAP2	8853	ArfGAP with SH3 domain, ankyrin repeat and PH domain 2
ENSG0000	73171.24	-2.28385	0.261804	-8.72353	2.70E-18	5.29E-16	ND1	4535	NADH dehydrogenase subunit 1
ENSG0000	65.49598	5.63471	0.646954	8.709593	3.05E-18	5.85E-16	GALNT14	79623	polypeptide N-acetylgalactosaminyltransferase 14
ENSG0000	45.99147	1.892202	0.21751	8.699372	3.34E-18	6.32E-16	NA	NA	NA
ENSG0000	316.7845	4.809651	0.5535	8.68952	3.64E-18	6.84E-16	AFF2	2334	ALF transcription elongation factor 2
ENSG0000	482.1564	-3.93787	0.454853	-8.65745	4.82E-18	8.88E-16	RRAD	6236	RRAD, Ras related glycolysis inhibitor and calcium channel regulator
ENSG0000	26.13387	8.109467	0.937493	8.650162	5.14E-18	9.40E-16	HOXD13	3239	homeobox D13
ENSG0000	76.18241	-3.62983	0.421711	-8.60738	7.48E-18	1.33E-15	SEMA6A	57556	semaphorin 6A
ENSG0000	141.955	2.397848	0.278877	8.598222	8.10E-18	1.43E-15	PM20D2	135293	peptidase M20 domain containing 2
ENSG0000	9867.907	-2.13242	0.248227	-8.59058	8.65E-18	1.52E-15	MTND1P2	100887749	MT-ND1 pseudogene 23
ENSG0000	24.16245	-3.41638	0.398603	-8.57087	1.03E-17	1.78E-15	CCDC148	130940	coiled-coil domain containing 148
ENSG0000	1159.25	-2.26773	0.265175	-8.55182	1.21E-17	2.07E-15	NA	NA	NA
ENSG0000	1010.053	1.61258	0.188906	8.536428	1.38E-17	2.34E-15	ASXL2	55252	ASXL transcriptional regulator 2
ENSG0000	53.22622	4.181076	0.491348	8.509397	1.75E-17	2.90E-15	FRMD3	257019	FERM domain containing 3
ENSG0000	1096.429	-1.73534	0.204497	-8.4859	2.14E-17	3.46E-15	CASTOR2	729438	cytosolic arginine sensor for mTORC1 subunit 2
ENSG0000	37.33692	1.958221	0.230817	8.483854	2.18E-17	3.48E-15	RPL7L1P8	402152	ribosomal protein L7 like 1 pseudogene 8
ENSG0000	923.4151	2.354164	0.27834	8.457885	2.72E-17	4.28E-15	TBX5-AS1	255480	TBX5 antisense RNA 1
ENSG0000	29599.82	-5.7782	0.683176	-8.45784	2.72E-17	4.28E-15	FOSB	2354	FosB proto-oncogene, AP-1 transcription factor subunit
ENSG0000	65.76035	-5.26009	0.624112	-8.42812	3.51E-17	5.48E-15	HOXC-AS1	100874363	HOXC cluster antisense RNA 1
ENSG0000	79.50866	3.77575	0.448174	8.424745	3.62E-17	5.61E-15	ICAM5	7087	intercellular adhesion molecule 5
ENSG0000	1595.657	-3.21939	0.384193	-8.37961	5.31E-17	8.01E-15	PLAC9	219348	placenta associated 9
ENSG0000	698.2541	2.240252	0.267664	8.369642	5.78E-17	8.66E-15	NHSL2	340527	NHS like 2
ENSG0000	121.4218	-1.58494	0.189599	-8.35944	6.30E-17	9.39E-15	C4orf48	401115	chromosome 4 open reading frame 48
ENSG0000	5752.596	-2.60995	0.312699	-8.34651	7.03E-17	1.04E-14	INSIG1	3638	insulin induced gene 1
ENSG0000	293.5769	1.743462	0.208962	8.343448	7.22E-17	1.06E-14	PTER	9317	phosphotriesterase related
ENSG0000	459.965	3.673196	0.440509	8.338527	7.52E-17	1.10E-14	UBASH3B	84959	ubiquitin associated and SH3 domain containing B
ENSG0000	280.7702	6.379967	0.765482	8.33458	7.78E-17	1.13E-14	VAT1L	57687	vesicle amine transport 1 like
ENSG0000	391.9776	-2.3091	0.277797	-8.31218	9.40E-17	1.36E-14	NA	NA	NA
ENSG0000	205.7029	2.41698	0.291569	8.289569	1.14E-16	1.62E-14	RHOU	58480	ras homolog family member U
ENSG0000	341.4118	-4.16787	0.504594	-8.25984	1.46E-16	2.03E-14	TBX1	6899	T-box transcription factor 1
ENSG0000	1172.414	1.838384	0.222711	8.254581	1.52E-16	2.11E-14	PPP2R3A	5523	protein phosphatase 2 regulatory subunit B''alpha
ENSG0000	58.4698	2.415476	0.292845	8.248297	1.61E-16	2.18E-14	ZNF543	125919	zinc finger protein 543
ENSG0000	374.3433	2.615238	0.318483	8.211559	2.18E-16	2.91E-14	SIX4	51804	SIX homeobox 4
ENSG0000	75.50882	-2.17535	0.26517	-8.20361	2.33E-16	3.07E-14	GASK1B-A	285505	GASK1B antisense RNA 1
ENSG0000	488.4314	1.678629	0.205373	8.173546	2.99E-16	3.86E-14	GCC1	79571	GRIP and coiled-coil domain containing 1
ENSG0000	1076.812	1.500651	0.183659	8.170836	3.06E-16	3.93E-14	PTGR3	284273	prostaglandin reductase 3
ENSG0000	728.4688	-5.40283	0.662507	-8.15513	3.49E-16	4.43E-14	INMT	11185	indolethylamine N-methyltransferase
ENSG0000	66.55601	-2.59048	0.318479	-8.1339	4.16E-16	5.16E-14	NA	NA	NA
ENSG0000	76.82237	-9.28712	1.14574	-8.10578	5.24E-16	6.47E-14	CAPN6	827	calpain 6
ENSG0000	38.46636	4.21863	0.520827	8.099872	5.50E-16	6.76E-14	SORL1	6653	sortilin related receptor 1
ENSG0000	25745.23	-3.5284	0.436234	-8.08831	6.05E-16	7.37E-14	ZFP36	7538	ZFP36 ring finger protein
ENSG0000	233.5962	-3.68558	0.456741	-8.06929	7.07E-16	8.54E-14	LIMS3	96626	LIM zinc finger domain containing 3
ENSG0000	855.618	1.585005	0.196793	8.054179	8.00E-16	9.62E-14	UBE4A	9354	ubiquitination factor E4A
ENSG0000	195.4302	1.50464	0.186926	8.049372	8.32E-16	9.96E-14	ZNF780A	284323	zinc finger protein 780A
ENSG0000	50.068	-3.40878	0.42448	-8.03048	9.71E-16	1.15E-13	IL20RA	53832	interleukin 20 receptor subunit alpha
ENSG0000	36.93249	-4.71987	0.58826	-8.02343	1.03E-15	1.21E-13	ITIH3	3699	inter-alpha-trypsin inhibitor heavy chain 3
ENSG0000	3155.176	-2.58825	0.323525	-8.00016	1.24E-15	1.45E-13	IDI1	3422	isopentenyl-diphosphate delta isomerase 1
ENSG0000	452.9632	1.511538	0.189098	7.99343	1.31E-15	1.53E-13	HMG20A	10363	high mobility group 20A
ENSG0000	24.80366	-6.27338	0.786837	-7.97291	1.55E-15	1.77E-13	NA	NA	NA
ENSG0000	1495.734	3.284871	0.412781	7.957894	1.75E-15	1.98E-13	CA12	771	carbonic anhydrase 12
ENSG0000	1262.082	2.874779	0.361969	7.942061	1.99E-15	2.22E-13	EHD3	30845	EH domain containing 3
ENSG0000	92.19252	2.035511	0.256474	7.936506	2.08E-15	2.30E-13	ZNF501	115560	zinc finger protein 501
ENSG0000	38.7552	-5.32841	0.672099	-7.92802	2.23E-15	2.46E-13	NA	NA	NA
ENSG0000	158.6501	9.745303	1.230309	7.921019	2.36E-15	2.58E-13	EIF1AY	9086	eukaryotic translation initiation factor 1A Y-linked
ENSG0000	396.0662	-2.9069	0.367142	-7.91765	2.42E-15	2.64E-13	CDKN1C	1028	cyclin dependent kinase inhibitor 1C
ENSG0000	70.18285	-3.03033	0.383518	-7.9014	2.76E-15	2.96E-13	ATP6V1FN	100130705	ATP6V1F neighbor
ENSG0000	1662.764	-2.32073	0.294239	-7.88722	3.09E-15	3.30E-13	KCNE4	23704	potassium voltage-gated channel subfamily E regulatory subunit 4
ENSG0000	1387.29	-1.97461	0.250502	-7.88262	3.21E-15	3.41E-13	PLK3	1263	polo like kinase 3
ENSG0000	108.6193	2.009315	0.255325	7.869641	3.56E-15	3.77E-13	SUPT16HP	400011	SPT16 homolog, facilitates chromatin remodeling subunit pseudogene 1
ENSG0000	226.3886	-3.69663	0.469841	-7.86784	3.61E-15	3.81E-13	LIMS4	100288695	LIM zinc finger domain containing 4
ENSG0000	4338.137	-2.43188	0.309606	-7.85476	4.01E-15	4.17E-13	SAT1	6303	spermidine/spermine N1-acetyltransferase 1
ENSG0000	80.29728	3.980059	0.506772	7.853746	4.04E-15	4.19E-13	CDCA7	83879	cell division cycle associated 7
ENSG0000	25.72405	-5.73831	0.731238	-7.84739	4.25E-15	4.38E-13	NA	NA	NA
ENSG0000	120.4304	-1.80974	0.231031	-7.83331	4.75E-15	4.86E-13	ZNF682	91120	zinc finger protein 682
ENSG0000	1004.733	-1.52499	0.195109	-7.81612	5.45E-15	5.53E-13	ARL4D	379	ADP ribosylation factor like GTPase 4D
ENSG0000	362.4217	2.38233	0.305558	7.796661	6.36E-15	6.38E-13	FRMD4A	55691	FERM domain containing 4A
ENSG0000	270.048	1.669865	0.214203	7.795717	6.40E-15	6.40E-13	CSNK2A3	283106	casein kinase 2 alpha 3
ENSG0000	50.48811	-3.95574	0.508251	-7.78303	7.08E-15	7.00E-13	NA	NA	NA
ENSG0000	842.7186	10.43356	1.341132	7.779665	7.27E-15	7.16E-13	DDX3Y	8653	DEAD-box helicase 3 Y-linked
ENSG0000	34.04279	-3.83835	0.493865	-7.77206	7.72E-15	7.55E-13	NA	NA	NA
ENSG0000	33.59549	1.893819	0.243892	7.764976	8.17E-15	7.95E-13	RPL7L1P9	100128899	ribosomal protein L7 like 1 pseudogene 9
ENSG0000	350.8249	-3.71987	0.479689	-7.75476	8.85E-15	8.56E-13	EML6	400954	EMAP like 6
ENSG0000	113.8876	1.513447	0.195806	7.729303	1.08E-14	1.03E-12	SARM1	23098	sterile alpha and TIR motif containing 1
ENSG0000	138440.6	-1.94644	0.25215	-7.71935	1.17E-14	1.09E-12	MTCO1P1	107075141	MT-CO1 pseudogene 12
ENSG0000	71.83479	1.579016	0.204708	7.713505	1.22E-14	1.14E-12	LINC02987	101927151	long intergenic non-protein coding RNA 2987
ENSG0000	111.4915	-4.75875	0.617037	-7.71225	1.24E-14	1.15E-12	EXPH5	23086	exophilin 5
ENSG0000	583.0137	-4.50554	0.584917	-7.70287	1.33E-14	1.22E-12	ABCA10	10349	ATP binding cassette subfamily A member 10

ENSG0000	1021.71	-5.41592	0.704335	-7.68941	1.48E-14	1.36E-12	FABP3	2170	fatty acid binding protein 3
ENSG0000	2512.268	5.031316	0.656509	7.663744	1.81E-14	1.62E-12	ENPP1	5167	ectonucleotide pyrophosphatase/phosphodiesterase 1
ENSG0000	175.1274	2.328193	0.303895	7.661165	1.84E-14	1.65E-12	MBNL3	55796	muscleblind like splicing regulator 3
ENSG0000	196797.3	-1.985	0.259341	-7.65402	1.95E-14	1.73E-12	ND4	4538	NADH dehydrogenase subunit 4
ENSG0000	46.81836	-1.64439	0.21501	-7.64795	2.04E-14	1.80E-12	ATP6AP1-	158960	ATP6AP1 divergent transcript
ENSG0000	75.6988	-4.76642	0.62364	-7.6429	2.12E-14	1.85E-12	TMEM88	92162	transmembrane protein 88
ENSG0000	517.4082	2.660464	0.348417	7.635868	2.24E-14	1.94E-12	THSD4	79875	thrombospondin type 1 domain containing 4
ENSG0000	57.43588	4.468204	0.585473	7.63179	2.32E-14	1.99E-12	NA	NA	NA
ENSG0000	45.33787	-4.60448	0.603406	-7.63081	2.33E-14	2.00E-12	NA	NA	NA
ENSG0000	69.98122	-2.33145	0.305848	-7.62292	2.48E-14	2.12E-12	NA	NA	NA
ENSG0000	42.17259	-1.79207	0.236563	-7.57543	3.58E-14	3.01E-12	NA	NA	NA
ENSG0000	40.06613	2.333696	0.308159	7.573023	3.65E-14	3.06E-12	MAP10	54627	microtubule associated protein 10
ENSG0000	32.12284	-2.69676	0.356154	-7.57188	3.68E-14	3.07E-12	NA	NA	NA
ENSG0000	49.14957	9.01836	1.191069	7.571654	3.69E-14	3.07E-12	NA	NA	NA
ENSG0000	95.99523	8.543133	1.130256	7.558583	4.07E-14	3.38E-12	SHOX	6473	short stature homeobox
ENSG0000	235.5587	2.447341	0.324015	7.553168	4.25E-14	3.50E-12	CNIH3	149111	cornichon family AMPA receptor auxiliary protein 3
ENSG0000	53.93909	-7.80817	1.034709	-7.54625	4.48E-14	3.66E-12	NPNT	255743	nephronectin
ENSG0000	6411.784	-2.23815	0.29661	-7.54576	4.50E-14	3.66E-12	CCNL1	57018	cyclin L1
ENSG0000	23.86668	-3.47719	0.461035	-7.54213	4.62E-14	3.74E-12	FLJ12825	440101	uncharacterized LOC440101
ENSG0000	20.54272	-4.22768	0.560646	-7.54073	4.67E-14	3.76E-12	NA	NA	NA
ENSG0000	376.4658	-1.6855	0.22357	-7.53905	4.73E-14	3.79E-12	INTS6L	203522	integrator complex subunit 6 like
ENSG0000	70.35851	1.763044	0.234265	7.525847	5.24E-14	4.17E-12	NA	NA	NA
ENSG0000	417249	-1.95067	0.259456	-7.51831	5.55E-14	4.37E-12	COX1	4512	cytochrome c oxidase subunit I
ENSG0000	261.196	1.708592	0.227848	7.498828	6.44E-14	5.05E-12	ZBTB6	10773	zinc finger and BTB domain containing 6
ENSG0000	46.21098	1.947751	0.260064	7.489517	6.91E-14	5.39E-12	NA	NA	NA
ENSG0000	2768.068	-3.19402	0.42661	-7.48697	7.05E-14	5.48E-12	ZC3H12A	80149	zinc finger CCCH-type containing 12A
ENSG0000	23.09838	-3.31275	0.442513	-7.48622	7.09E-14	5.48E-12	SLC52A1	55065	solute carrier family 52 member 1
ENSG0000	670.1142	-2.74418	0.366604	-7.48541	7.13E-14	5.50E-12	RASD1	51655	ras related dexamethasone induced 1
ENSG0000	160.75	1.798934	0.241361	7.453289	9.10E-14	6.84E-12	NA	NA	NA
ENSG0000	1137.111	1.777016	0.23851	7.450502	9.30E-14	6.97E-12	SYT11	23208	synaptotagmin 11
ENSG0000	454.2461	-2.23233	0.299989	-7.44136	9.97E-14	7.45E-12	SLC19A2	10560	solute carrier family 19 member 2
ENSG0000	575.7942	2.457668	0.331007	7.424833	1.13E-13	8.37E-12	ZNF770	54989	zinc finger protein 770
ENSG0000	1346.564	2.552883	0.343988	7.421423	1.16E-13	8.54E-12	ZNF281	23528	zinc finger protein 281
ENSG0000	572.4333	10.36745	1.397806	7.416948	1.20E-13	8.81E-12	USP9Y	8287	ubiquitin specific peptidase 9 Y-linked

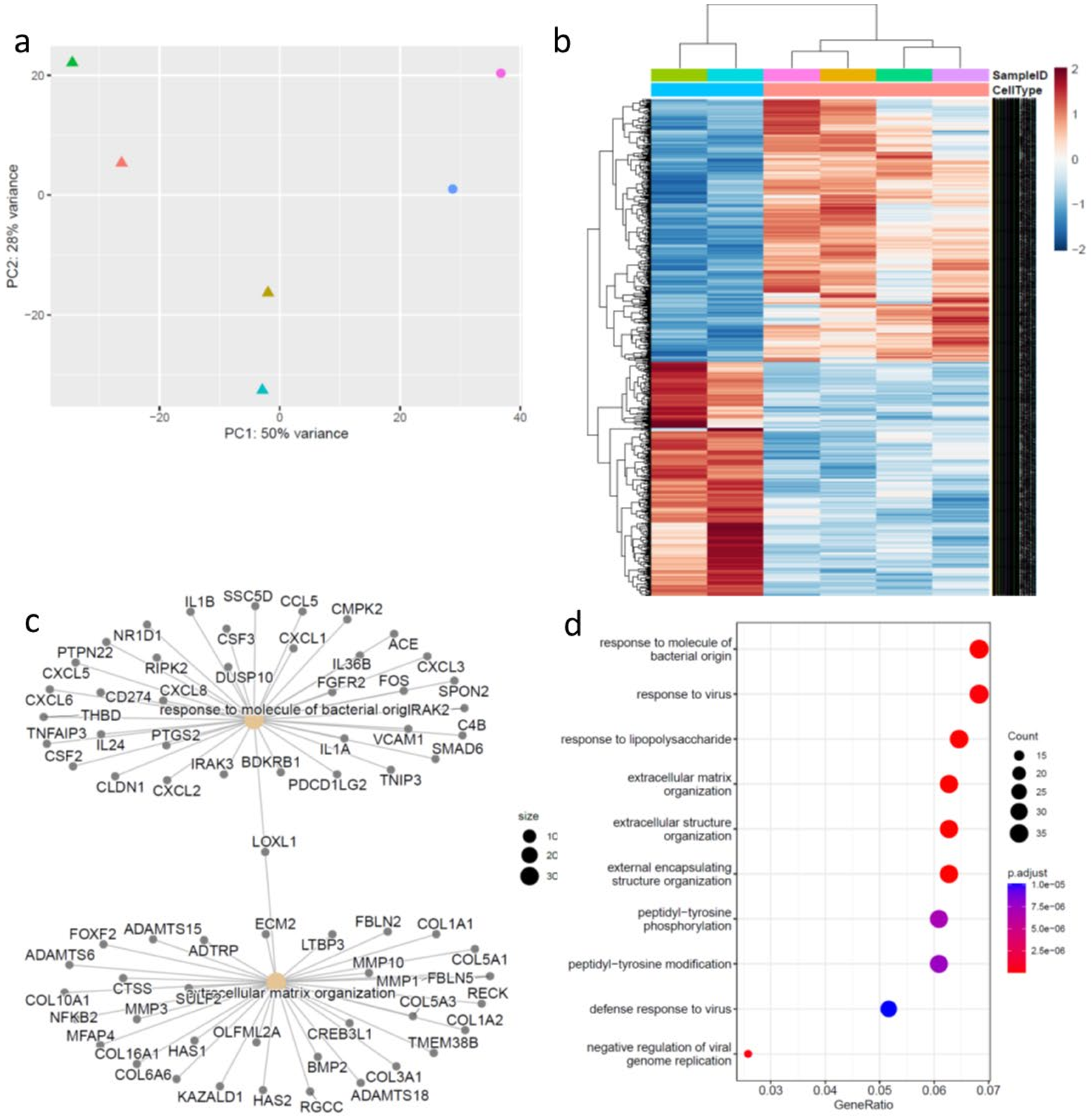

Supp Figure 1. **Differentially expressed genes identified in Dupuytren disease cord fibroblasts compared to WNT4scrambledsiRNA.** (A) PCA analysis of the DDC fibroblasts and ScramsiRNA (triangle is DDC, circle is ScramsiRNA). (B) Heatmap showing clustering of significantly differentially expressed genes in DDC (cream) vs ScramsiRNA (blue) fibroblasts. (C) Concept network plot representing the expression of genes from functional categories associated with extracellular matrix. The selected genes were identified based on GO analysis. (D) GO analysis identified over-representative GO terms for the DEGs in treated fibroblasts. The top 10 significantly enriched GO terms in the biological pathway are shown and include extracellular matrix and structure and organization.
